# Low-level laser treatment applied at auriculotherapy points to reduce postoperative pain in third molar surgery: A randomized, controlled, single-blinded study

**DOI:** 10.1371/journal.pone.0197989

**Published:** 2018-06-19

**Authors:** Hélio Sampaio-Filho, Sandra Kalil Bussadori, Marcela Leticia Leal Gonçalves, Daniela de Fátima Teixeira da Silva, Maria Cristina Borsatto, Isabel Peixoto Tortamano, Priscila Larcher Longo, Christiane Pavani, Kristianne Porta Santos Fernandes, Raquel Agnelli Mesquita-Ferrari, Anna Carolina Ratto Tempestini Horliana

**Affiliations:** 1 Postgraduate Program in Biophotonics Applied to Health Sciences, Universidade Nove de Julho, UNINOVE, São Paulo, SP, Brazil; 2 Postgraduate Program in Rehabilitation Sciences, Universidade Nove de Julho, UNINOVE, São Paulo, SP, Brazil; 3 Department of Odontopediatrics Clinic, São Paulo University, Ribeirão Preto, SP, Brazil; 4 Department of Estomatology, São Paulo University, São Paulo, SP, Brazil; 5 Department of Microbiology, São Paulo University, São Paulo, SP, Brazil; Stanford University School of Medicine, UNITED STATES

## Abstract

**Objective:**

Evaluate the effectiveness of LLL (Low level laser therapy) in auriculotherapy points for pain reduction following lower third molar extractions.

**Study design:**

Randomized, controlled, single-blinded study.

**Methods:**

Eighty-four bilateral, symmetrical third molar surgeries were performed in 42 healthy patients using a split-mouth design. In the immediate postoperative period, each side was randomly treated in a single-blind method with an LLL at the auriculotherapy points or simulation of its use (contralateral side) over a 21-day interval. This protocol was repeated 24 and 48 hours after surgery. All patients used the same analgesic (paracetamol) but only in case of pain. The primary variable was postoperative pain according to the visual analogue scale, and the secondary variables were mouth opening, edema, local temperature, dysphagia, and the presence of infection (systemic temperature, lymphadenopathy). These variables were evaluated at baseline and at 24 hours, 48 hours and seven days after surgery. Adverse effects were recorded and reported.

**Results:**

There was no difference between the groups in relation to any of the evaluated parameters (p>0.05).

**Conclusion:**

For this experimental model, application of a low-intensity laser at auriculotherapy points did not prevent postoperative pain following lower third molar surgery.

**Trial registration:**

This trial is registered at ClinicalTrials.gov; the registration number is NCT02657174 and the Unique Protocol ID number is 1.100.869. (https://register.clinicaltrials.gov/prs/app/template/EditRecord.vm?epmode=View&listmode=Edit&uid=U0002BEY&ts=11&sid=S0006026&cx=6g4wff).

## Introduction

The third molar extraction postoperative period is accompanied by pain and edema; the control of which are essential for both the patient and dental surgeon. The intense inflammatory reaction within the three first days of the postoperative period compromises the quality of life for patients [1,2,3,4]. The most intense pain occurs mainly on the first day, 3–5 hours after the anesthetic effect ceases [5,6]. The most common local complications include alveolar osteitis (dry alveolitis), edema, reduced mouth opening, abscess and pain; the most common systemic complications include fever and lymph node alteration [7,8]. Some of these complications, especially pain, mouth opening reduction and edema, can be minimized. Corticosteroids and non-steroidal anti-inflammatory drugs are frequently used. However, adverse reactions, such as gastrointestinal disorders (erosions, ulcers, dyspepsia) with serious hemorrhagic complications, cardiovascular disorders, renal failure and platelet abnormalities have been reported; thus, these drugs should be avoided in patients with certain conditions, mainly hypertension and diabetes [7–12].

Acupuncture has been used for many years in several areas of healthcare to control postoperative pain [13,14], and acupuncture has been the subject of recent studies [14–20]. Acupuncture consists of stimulation of certain points distributed along the body surface using needles, moxibustion, electricity, acupressure or a laser [21,22] with effective results.

In auriculotherapy practice, various modalities can be adopted including auricular acupuncture, auricular electroacupuncture, acupressure, moxibustion, injection, and auricular bloodletting therapy [23,10,14,16,18]. Other possibilities in this area include ‘moxa seeds’ from *Artemisia vulgaris*. This plant appears to have unique features.

Auriculotherapy can be defined as a system of diagnosis and treatment through stimulation of localized points on the ear [8,20,24] in which the therapeutic intervention treats various parts of the body [20,25]. Stimulation of these points involves neurological reflexes, neurotransmitters, cytokines, the immune system and inflammation [24]. To date, only one clinical study [26] has evaluated pain reduction through application of laser auriculotherapy; however, this study did not investigate the dental area (reduction of ipsilateral wrist pain was assessed). Studies are necessary to demonstrate the efficacy of auriculotherapy associated with a low-level laser to control pain, reduced mouth opening, systemic inflammation and edema after lower third molar extraction. Thus, the objective of this study was to evaluate the effectiveness of an LLL in auriculotherapy points for pain reduction following lower third molar extractions.

## Materials and methods

This is a randomized, controlled, single-blinded, split-mouth study. The study was approved by the Research Ethics Committee of Nove de Julho University (UNINOVE) under number 1.100.869 on June 10^th^, 2015 (S1–S4 Files). At that time, we were planning to start the study in August 2015. However, we needed to register the study and publish the protocol (S5 File), so the recruitment was delayed until July 2016. Again, we had planned to start the study (recruitment and surgeries) in June 2016 [27]. However, in June 2016, we only performed the research calibration and radiograph analysis. Patient recruitment and surgeries started in July 2016.

Thus, the dates of this study were as follows: the study was registered (ClinicalTrials.gov) on January 11^th^, 2016 (before patient recruitment). The protocol was submitted for publication in Trials [27] on January 16^th^, 2016. The study started (research calibration and radiograph analysis) in June 2016. Recruitment of the participants for this study started in July 2016, which was six months after the study was registered on clinicaltrials.gov. Primary completion of the study occurred in February 2017, and study completion (including follow up) occurred in April 2017. The authors confirm that all ongoing and related trials for this drug/intervention are registered. This study follows CONSORT statement (S6 File)

Surgeries were performed at the Dental Clinic of Nove de Julho University–UNINOVE and at the Military Police Dental Center of São Paulo State in the city of São Paulo, Brazil. In June 2015, when our protocol was approved by the Research Ethics Committee, the plan was to perform 120 surgeries on 60 patients based on the scenario with largest required sample size. At that time, we had not found any similar studies to compare the differences between the mean values in auriculotherapy field and pain. Subsequently, we found a study that evaluated pain after auriculotherapy treatment. Therefore, we decided to re-calculate the sample size for better accuracy. The mean values of the control and treated groups, as well as SD, were obtained from this study [28] and the type 2 error probability was set at 0.05, corresponding to a statistical power of 0.95. According to the calculation, a sample of 45 patients was necessary to detect differences in pain. When registering the study at clinicaltrials.gov and submitting this protocol to Trials, we used the new sample size calculation (45 patients). Lews’ study [28] was the most similar study compared with our study. These authors used auriculotherapy treatment, and the measurement of pain was the primary variable of the study. A sample size calculation was performed (G* Power software version 3.1.9.2) to provide power analysis of 95%. The sample size should comprise 45 patients in each group to detect differences in postoperative pain. We decided to finish the research ahead of time after realizing that only 3 patients were missing in the study and that there was no obvious trend towards a difference between the groups. We considered that the two groups behaved identically. Thus, forty-two patients had surgery, but we excluded 4 patients. One of the patients was excluded due to omission of systemic problems during anamnesis. Another patient had a postoperative infection, and two patients withdrew from the study during the postoperative period. Therefore, we included 38 patients and 76 surgeries in the statistical analysis.

Forty-two patients of both genders, aged between 18 and 28 years, underwent 84 surgical removals of bilateral and symmetric third molars. Two surgeries were performed in the same patient with a 21-day interval; thus, this was a split-mouth study. In general, the population is young healthy adults because these are the individuals who typically need to undergo third molar surgery.

The following patients were excluded from the study: pregnant or breastfeeding women, smokers, those who had undergone head and neck radiotherapy, those who were allergic to any drugs used in the research (such as paracetamol or 2% chlorhexidine), those with systemic or local infections (such as periodontal abscesses or pericoronitis), those who had used anti-inflammatory drugs in the last 3 months, those who used medications differently than the method in which they were prescribed, and those with lesions or radiolucent images associated with the teeth to be extracted. Patients who presented with complications during surgery (for example: operative difficulty, hemorrhages, or more than 90 minutes of surgery) were also excluded because these cases are not expected for third molar surgeries, and only in the aforementioned cases, a centrally acting analgesic was prescribed. These data were not part of the statistical analysis but were described and discussed including trans-surgical complications and possible adverse effects. Patients with teeth in position IIB [29] with indication for third molar extraction (recurrent infections, bad position, or orthodontic indication), written professional indication, healthy (negative medical history as per ASA I recommendation), systolic blood pressure lower than 140 mmHg, diastolic lower than 90 mmHg and heart rates of 70±20 beats/minute were included.

**Fig 1:**
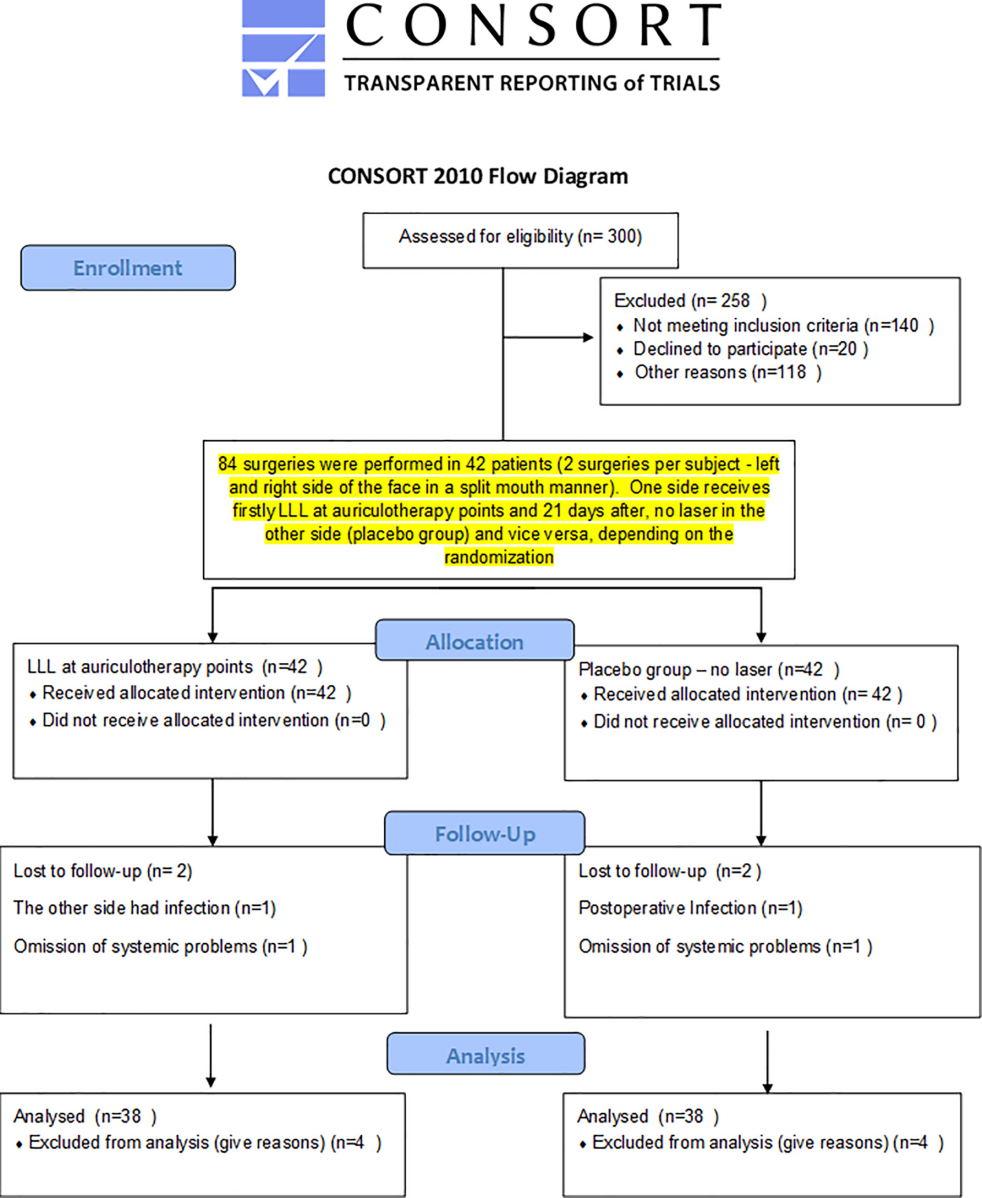
Activity flowchart.

An external researcher (I.P.T.) performed the randomization (Microsoft Excel, version 2013). The randomization was blocked in 45 pairs of numbers (1:1), i.e., 45 AB or BA blocks. Neither the patient nor the surgeon or those assessing the outcomes knew which treatment was applied in each of the two surgeries. The only individual who was aware of the treatment performed was the acupuncturist who applied the laser (single-blinded study). The drawn treatments (A or B) were placed inside opaque envelopes identified with sequential numbers. The envelopes were sealed and remained sealed in the same numerical order in a secure place until the surgery was performed. The study design is shown in Fig 1.

**Fig 2:**
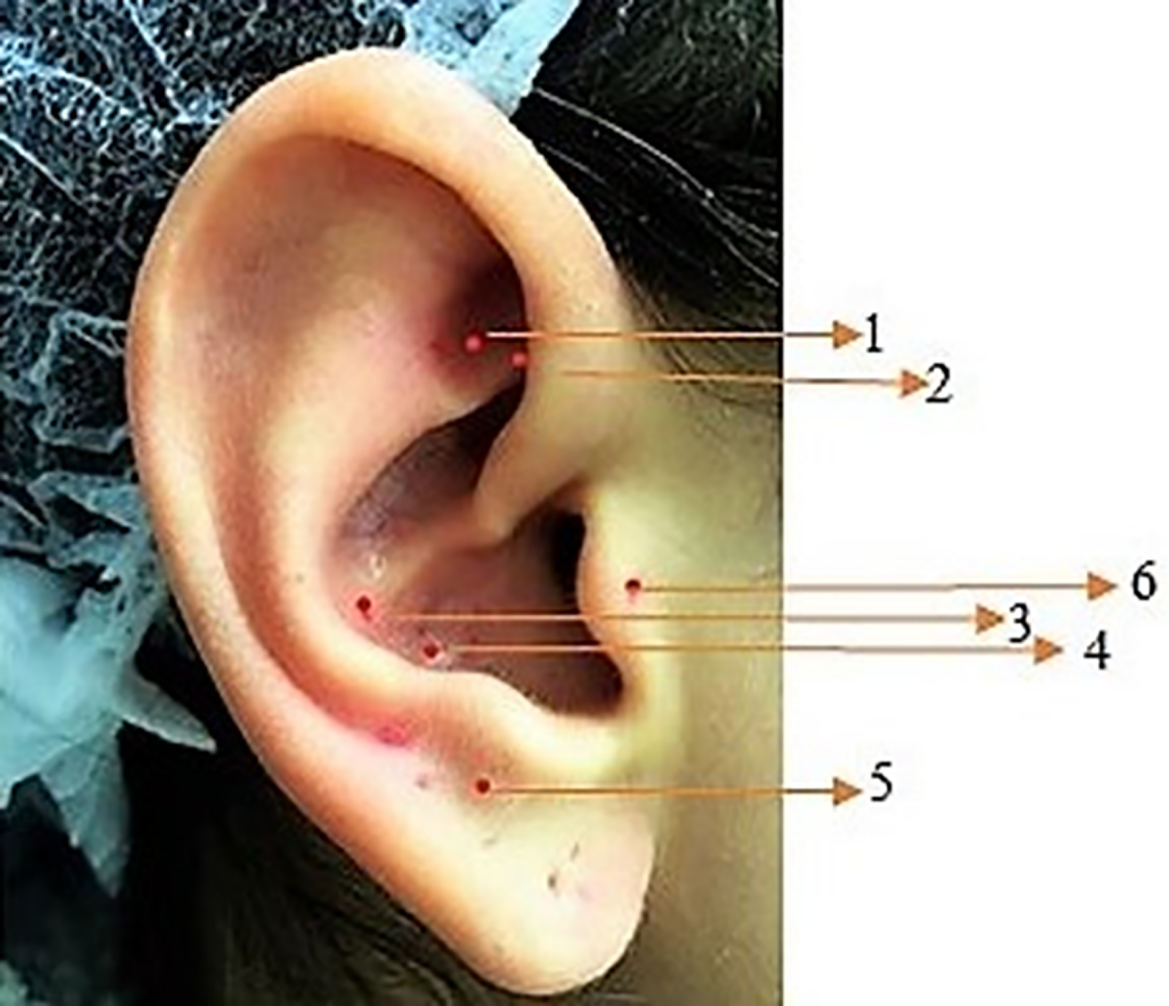
Auriculotherapy points. 1) Shen Men, 2) Sympathetic (SNV), 3) Stomach, 4) Toothache 3, 5) Jaw, 6) Adrenal.

Each patient served as his or her own control:

G1 - (experimental) (n = 42 surgeries)–surgery was performed in a conventional manner, and the patient received LLL treatment at auriculotherapy points to prevent pain and inflammation in the immediate postoperative period and within 24 and 48 hours after intervention (Fig 2).

G2 - (control) (n = 42 surgeries)–the procedures were performed in the same way as in Group 1 but with the laser turned off.

The use of a verum or placebo laser was randomized for the first surgery. For the second surgery, the opposite treatment was performed (i.e., if the first surgery was A (random), then the second surgery must be B and vice-versa). We did this to prevent the patient from receiving the same treatment twice.

**Fig 3:**
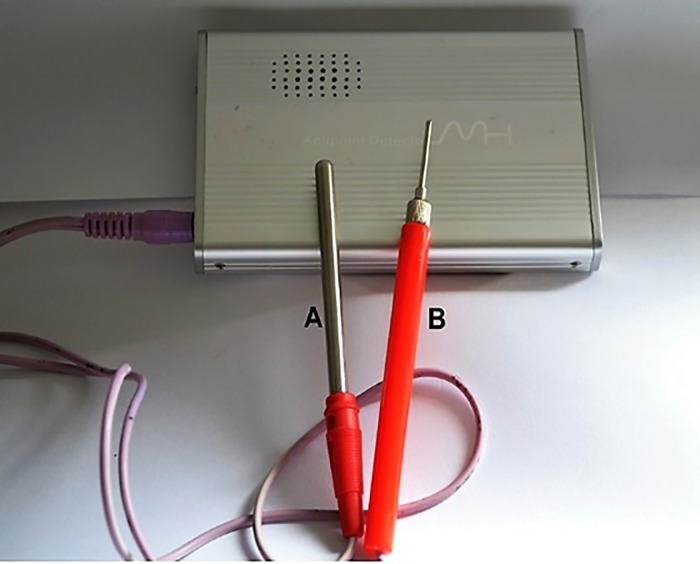
Acupoint detector MH-II^®^. (A) patient hand probe (B) hand-piece for detection.

One researcher assigned participants to their interventions (HSF). In both groups, postoperative doses were applied 24 h and 48 h after the intervention. For all patients, paracetamol Tylenol^®^ 500 mg every 8 hours was prescribed. All patients were instructed to use the medication only in case of pain. Additionally, in case of unbearable pain, a prescription of paracetamol with codeine phosphate 30 mg Tylex^®^ (Janssen-Cilag) was provided for ethical reasons. The medication data were described in the results analysis. The auriculotherapy points used in this study have been described previously [26]. A red diode laser (Therapy XT^®^) with a wavelength of 660 nm (±10 nm) and power of 100 mW was used. The optical fiber diameter was 600 μm, so the spot (area) was 0.002826 cm^2^. The energy delivered per point was 1 J over 10 seconds. Six points were irradiated, with a total energy of 6 J. The power density was 35.4 mW/cm^2^. For accuracy in determining the points, we used the ear locator for auriculopuncture (Acupoint detector MH-II^®^, Japan), which is based on the principles of electrical potential least resistance, allowing greater accuracy [30] (Fig 3). A single operator performed the LLL treatment and placebo. J.S.R. performed all of the LLL applications in the auriculotherapy points of the right ear for the first and second interventions. She was calibrated before patent recruitment started and was the only person who knew which treatment was performed (verum or placebo laser).

All variables were evaluated by the same operator (G.N.) at baseline and after 24 hours, 48 hours and 7 days. The primary variable of the study was postoperative pain, which was assessed by visual analogue scale (VAS). The secondary variables included mouth opening, edema, local temperature, dysphagia and presence of infection (systemic temperature or lymphadenopathy).

Postoperative pain was assessed by applying the visual analog scale (VAS) with a 100-mm line with two ends including "0" meaning no pain and "100" meaning unbearable pain; the VAS was always evaluated by the same operator. Each patient was instructed to mark with a vertical dash the point that best corresponded to his or her pain intensity at the time of evaluation [31]. Edema measurements were based on a previous study [32] and described in a protocol [27] in which 3 measures were performed: I) Eye Corner, up to the angle of the jaw; (II) Tragus to commissure lip; and (III) Tragus to Pogona’s. For mouth opening evaluation, the inter-incisal measurement in millimeters was measured with a digital caliper (Mitutoyo, model Digimatic Caliper, Japan). The distance between the incisal border of the right upper and lower central incisors was measured. Before beginning the surgery, the patient was asked to perform his/her maximum mouth opening [32]. Thus, this is a clinical variable that demonstrates the amount of exudate spread in the region adjacent to the 3^rd^ molar (including muscular tissue). Difficulty in mouth opening shows more exudate and higher levels of inflammation. The temperature was measured locally because of local inflammation and systemically to detect fever and verify the presence of an infection. Systemic temperature was measured 3 cm above the glabella using a digital thermometer (Safety 1st^®^, "No Touch Forehead" model, Columbus, USA). The local measurement was performed in the jaw angle region 2 cm above the lower border of the mandible and 3 cm paramesial from mandibular branch, both on the operated and opposite sides. Dysphagia was also evaluated to complement the detection of clinical features of inflammation/infection. Dysphagia assessment was performed using a numerical scale as follows: (0) total absence of dysphagia; (1) dysphagia to solid food and (2) dysphagia to any liquid or solid food.

We decided to exclude 4 patients. One of the patients was excluded due to omission of systemic problems during anamnesis. Another patient had a postoperative infection, and two patients withdrew from the study during the postoperative period.

The data were analyzed using STATA 15. We chose to use mixed effects models, including random effects at the individual patient level in this analysis, and treated the data as an unbalanced panel, given that not all measurements were recorded for all patients at each time point. This approach is consistent with that described by Wooldridge (2010). All models included the variables sex, use of medication and length of surgery as covariates as these can be considered potential confounding variables. The models were evaluated with regards to the relationship between the primary (or secondary) outcome variables and the variable Group, which was set up as a dummy equal to zero if the patient had received a placebo treatment and equal to one if the patient received LLL treatment. Our null hypothesis was that the proposed protocol for LLL in auriculotherapy points is not effective for pain reduction following lower third molar surgeries. Statistical significance was declared at the 0.05 level. F-tests of joint significance and t-tests of individual significance were conducted and are reported for all models. Additionally, we fitted the predicted regression results against residual plots to evaluate normality, symmetry and independence, although given the use of the mixed methods models, those plots only referred to the fixed effects component of the general modeling equation. The regression results of the modeling performed can be found in the supplementary materials (S7 File) also the secondary analysis—mixed effect models (S8 File). The resulting plots can be found in the supplementary material (S1–S11 Figs).

When evaluating the presence of lymph nodes as a dependent (secondary) outcome variable, due to lack of variation in the data, we also performed a random effects model analysis, as the fixed effects component resulted in problems of multicolinearity.

The demographic data are presented in the tables (Table 1). Scatter plots of primary and secondary outcome measures by group can be found in the supplementary material.

**Table 1:** Demographic data.

Demographic and clinical variables	
**Male/female**	13/29
**Age**	24.5 ± 4.3
**Height**	1.64 ± 8.5
**Weight (kg)**	65 ± 9.2
**Body mass index**	24.2 ± 2

Data are expressed as the mean and standard deviation ± SD, kg = kilograms.

As well as the inferential analysis, we have compared means and standard deviations at four timepoints: baseline, postoperative, 24 hours, 48 hours and 7 days. The presented graphs compare means and standard deviations of all variables for the two groups in the four timepoints. Furthermore, given that our outcome measures are all time sensitive, we have explored mixed effects models in which measured outcomes in time 12hours, 24hours and 7 days have been used to explain the variation in the outcome measured at baseline. We acknowledge that this further analysis serves a different purpose, i.e. does not explain the variation between groups at any given time point, but it provides some reassuarance about the data collected, as it is expected that a significant relationship between any outcome at baseline and its repeated later measurements.

## Results

Forty-two patients underwent surgery, but we have excluded some participants from analysis. One of the patients was excluded due to omission of systemic problems during anamnesis. Another patient had a postoperative infection, and two patients withdrew from the study during the postoperative period. Thus, 38 patients and 76 surgeries are included in the statistical analysis.

The regression results of the modeling performed can be found in the supplementary materials (S7 File) also the secondary analysis—mixed effect models (S8 File)

Although they can each be examined individually, the results all point to the same direction and indicate that we cannot reject the null hypothesis as there was no significant difference between the placebo and the LLL treatment groups. It is worth noting that only the secondary outcome variables Edema (I), (II) and (III) and mouth opening passed the F-test of joint significance at p = 0.05.

There was no difference between the groups regarding the mean number of anesthetic cartridges (p = 0.19). The mean duration of surgery was 31 min in the laser group and 33 min in the placebo group (p = 0.409). The number of tablets required for postoperative pain management was 1.47± 2.50 in the laser group and 1.57 ± 3.12 in the placebo group.

**Fig 4:**
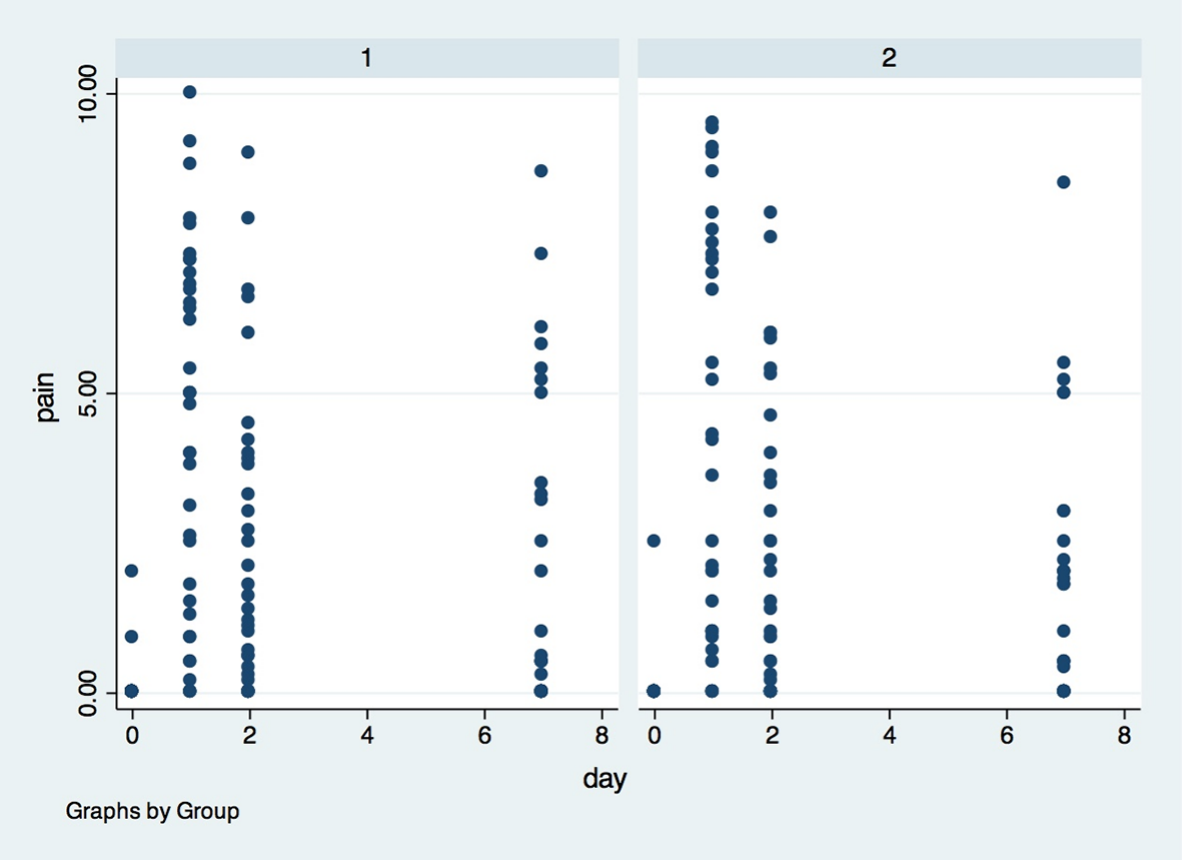
Postoperative pain data according to the visual analog scale (VAS) in both groups in all evaluation timepoints. X axis—Pain was measured in centimeters (1–10 cm) by Visual Analogue Scale (VAS), Y axis–measures were performed in baseline (0), 1, 2 and 7 days after surgery; (1)- placebo group, (2)- laser group.

There was no significant difference in postoperative pain between G1 and G2 at baseline (p>0.05), 24 hours (p>0.05) after surgery, 48 hours after surgery (p>0.05) or 7 days after surgery (p>0.05) (Fig 4). There was also no difference in average pain level between the groups.

**Fig 5:**
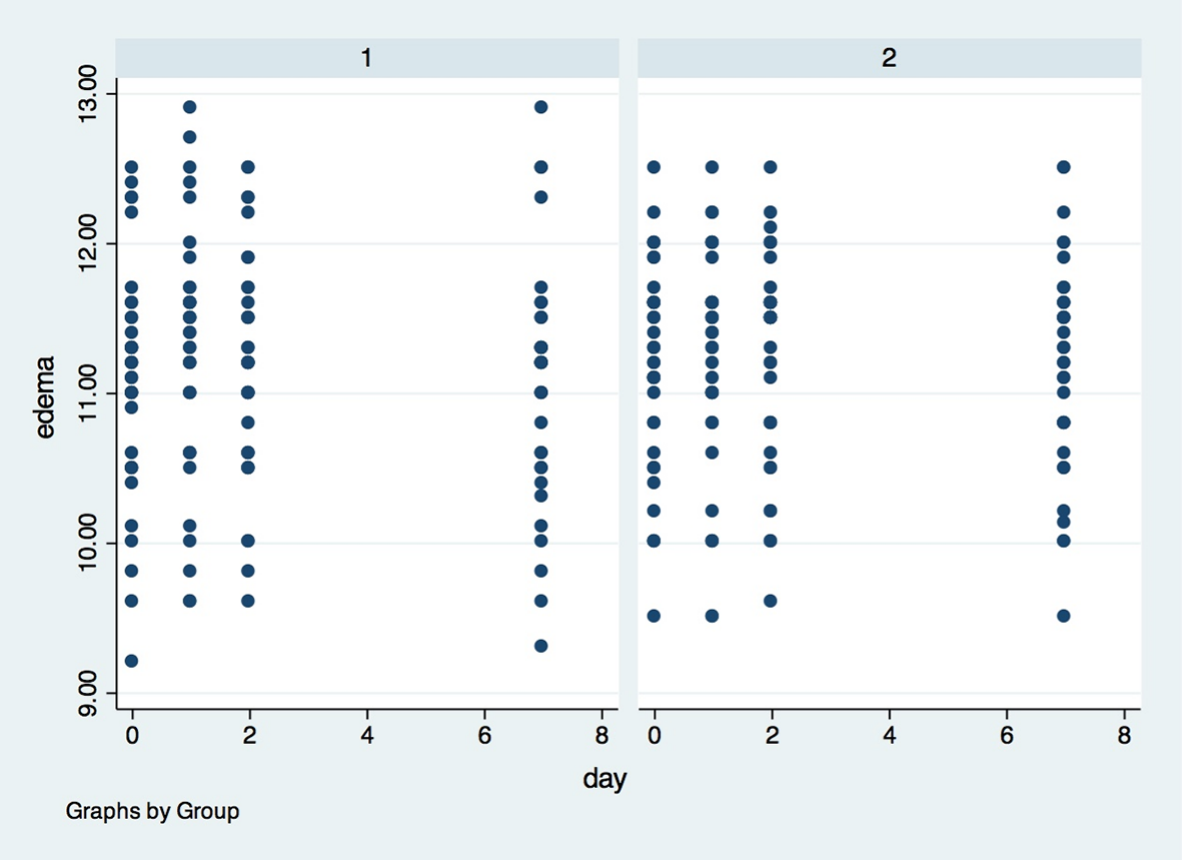
Presence of edema measured by the corner of the eye to angle of the jaw in the groups in all evaluation timepoints. X axis–Edema was measured in centimeters (1–10 cm) by the corner of the eye to angle of the jaw, Y axis–measures were performed in baseline (0), 1, 2 and 7 days after surgery; (1)- placebo group, (2)- laser group.

**Fig 6:**
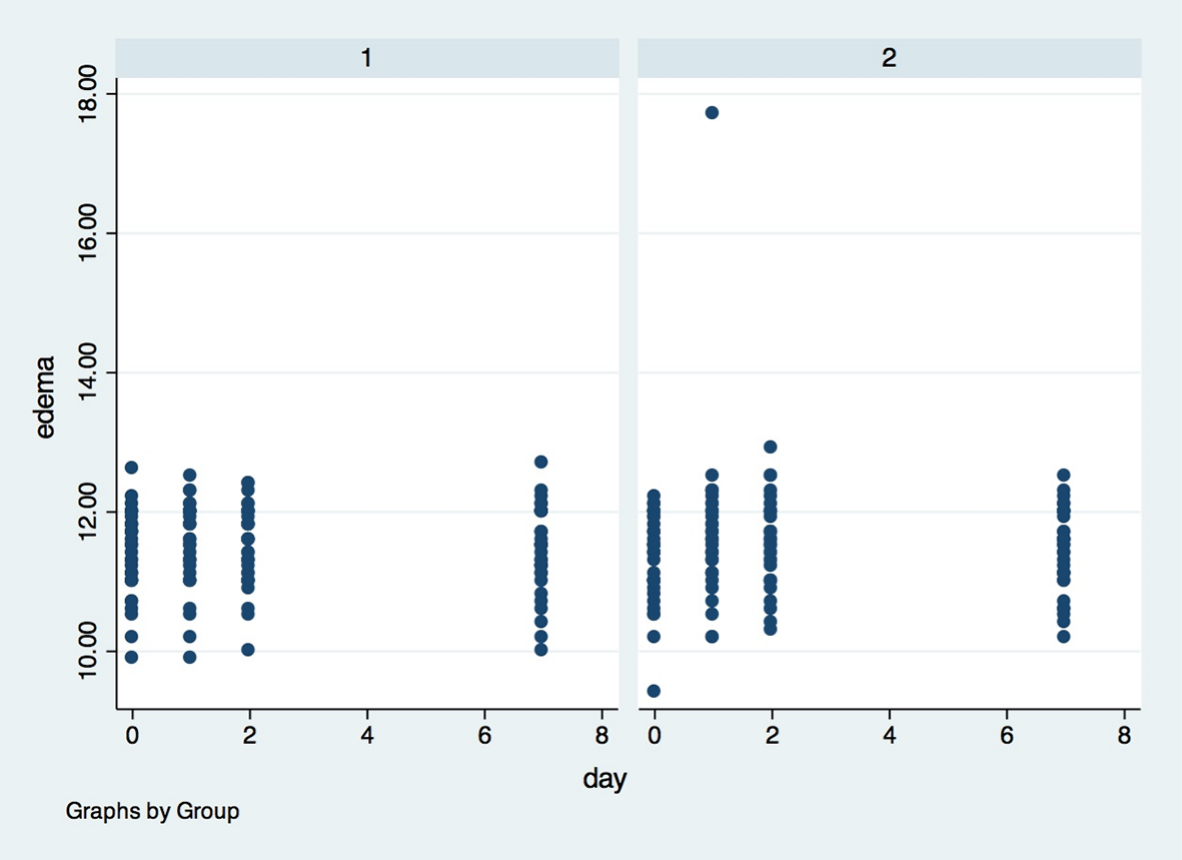
Presence of edema measured by the Tragus to the labial commissure in the groups in all evaluation timepoints. X axis–Edema was measured in centimeters (1–10 cm) by the (II) Tragus to the labial commissure, Y axis–measures were performed in baseline (0), 1, 2 and 7 days after surgery; (1)- placebo group, (2)- laser group.

**Fig 7:**
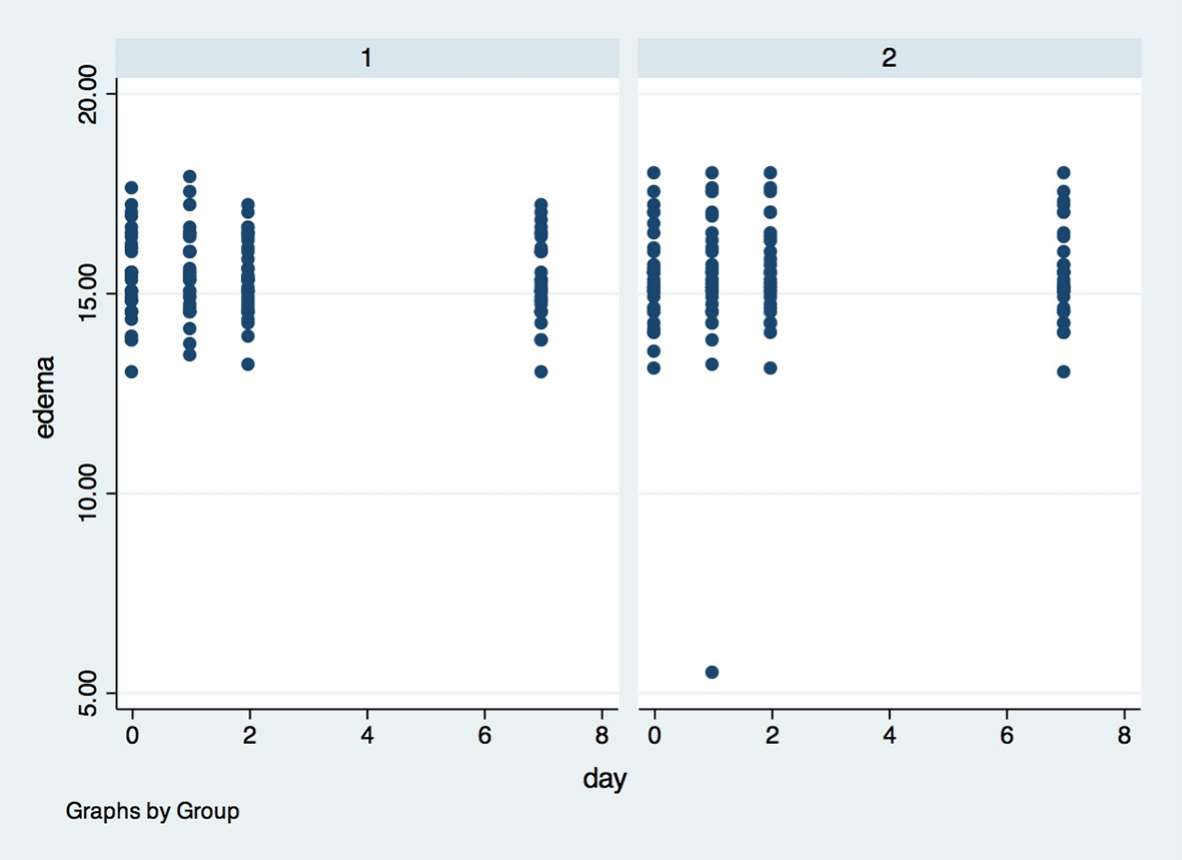
Presence of edema measured by the Tragus to pogonion in the groups in all evaluation timepoints. X axis–Edema was measured in centimeters (1–10 cm) by the (II) Tragus to the labial commissure, Y axis–measures were performed in baseline (0), 1, 2 and 7 days after surgery; (1)- placebo group, (2)- laser group.

For edema (Figs 5, 6 and 7), there was also no statistical difference between G1 and G2, in any of the evaluation timepoints (p>0.05). The determination of edema was based in 3 different measurements: (I) Corner of the eye to angle of the jaw (Fig 5); (II) Tragus to the labial commissure (Fig 6) and (III) Tragus to pogonion (Fig 7).

**Fig 8:**
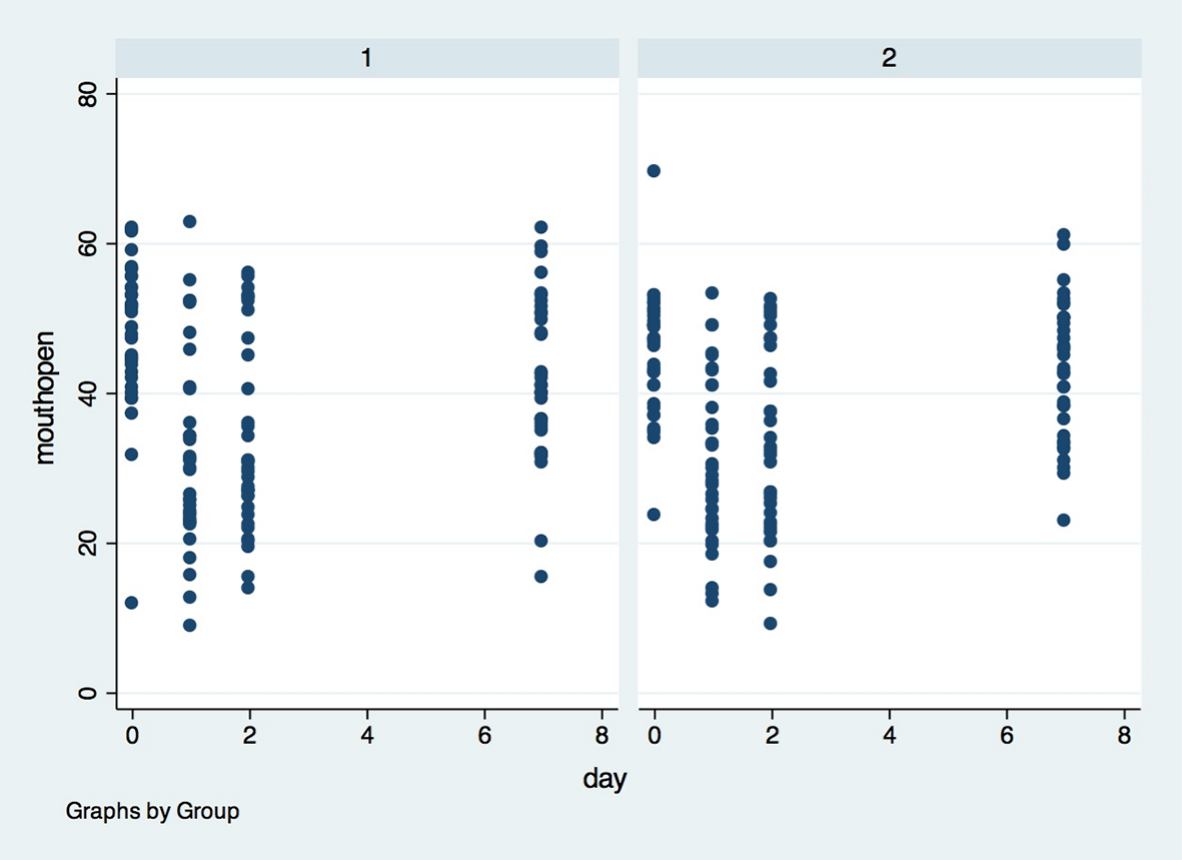
Mouth opening measurement in both groups in the four observed timepoints. X axis–Mouth opening was measured in centimeters (cm), Y axis–measures were performed in baseline (0), 1, 2 and 7 days after surgery; (1)- placebo group, (2)- laser group.

Regarding the variables, we performed the correlation test between them for each timepoint. The only correlation detected was in the 24-hour period between pain and mouth opening (Fig 8). The Pearson correlation coefficient was -0.257 and p = 0.036. The coefficient suggests that the stronger the pain, the smaller the mouth opening.

**Fig 9:**
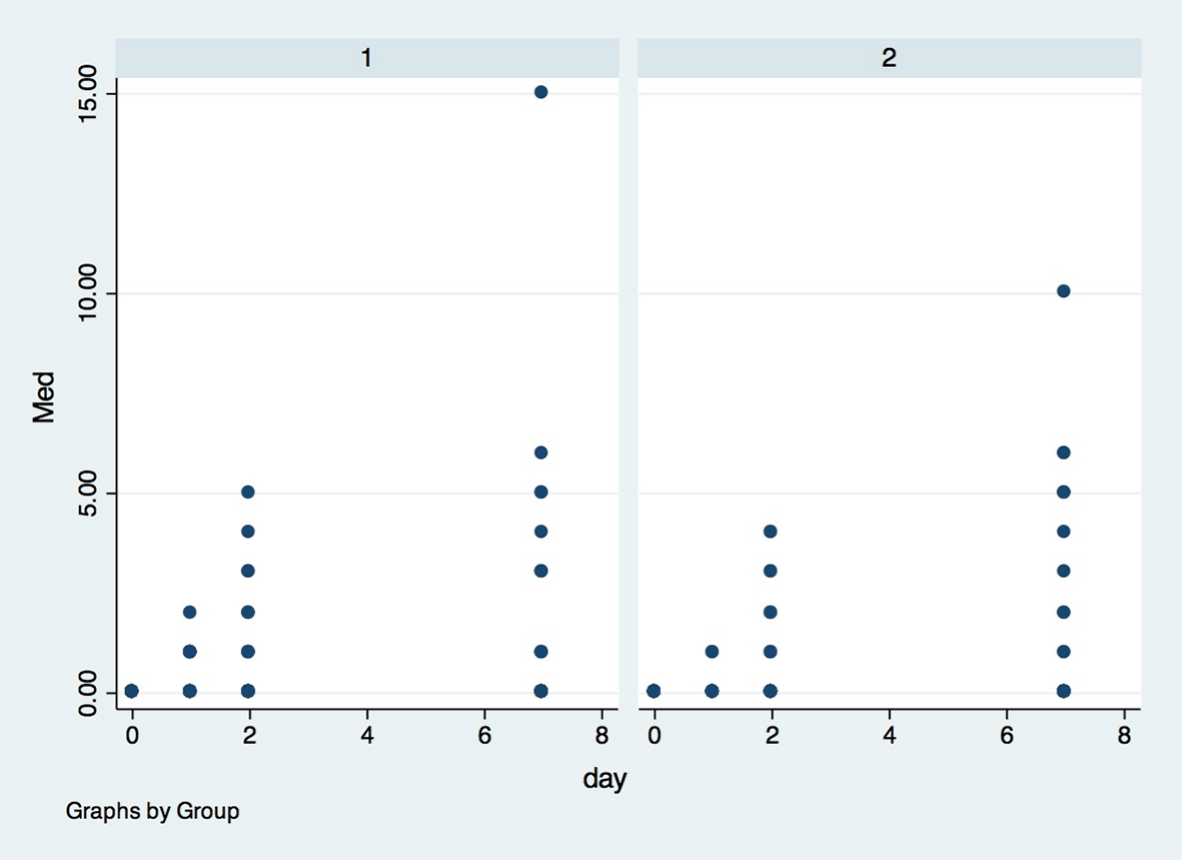
Intake of pain medication in both groups in the four observed timepoints. X axis–medicines ingested in absolute numbers, Y axis–measures were performed in baseline (0), 1, 2 and 7 days after surgery; (1)- placebo group, (2)- laser group.

The intake of pain medication was described in Table 2 for each treatment group and Fig 9. The placebo group and laser group had the same medication intake (p<0.420).

**Table 2:** Description of the mean ± SD pain medication intake for the laser and placebo groups.

Intake of pain medication			
	Mean	Standard deviation (±)	p-value
**Placebo Group**	1.57	3.1259022	0.420
**Laser Group**	1.47	2.5014938	

Data are expressed as the mean and standard deviation ± SD

**Fig 10:**
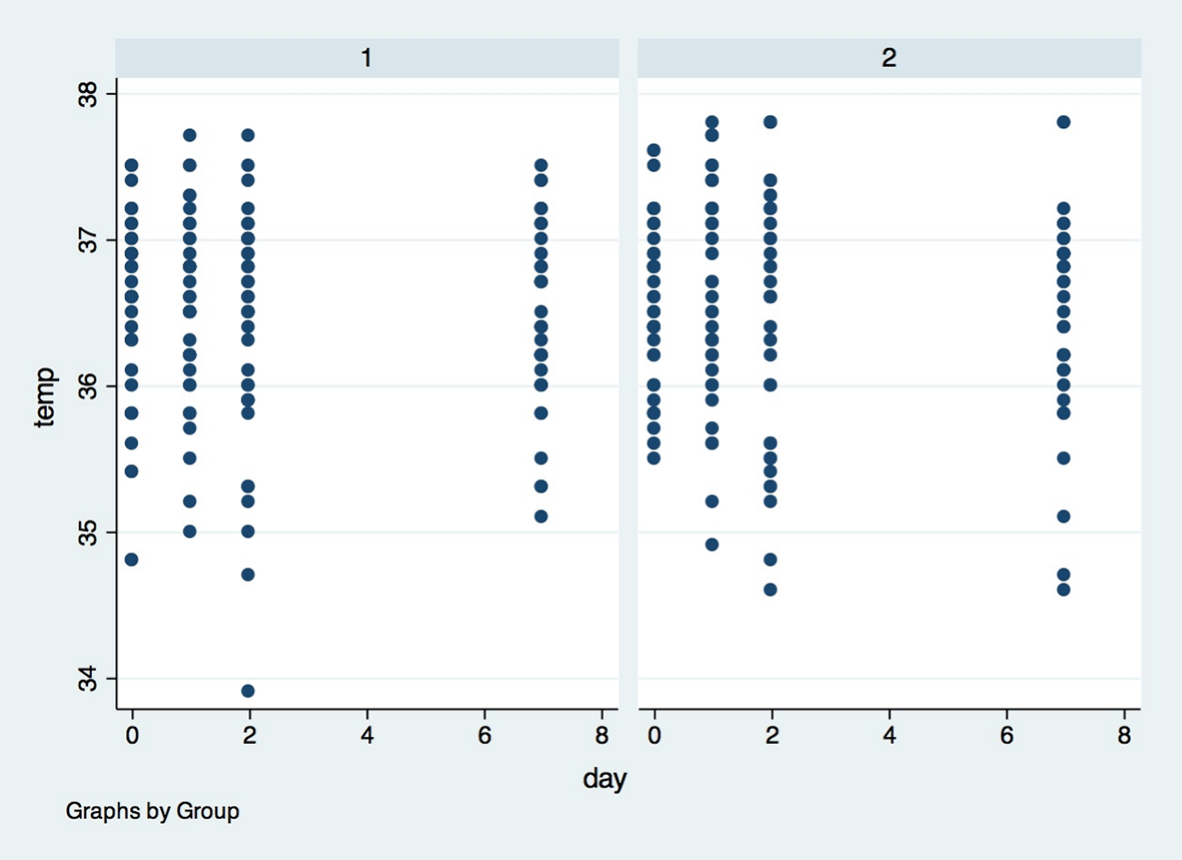
Temperature of operated side in both groups in the four observed timepoints. X axis–Temperature of operated side was measured in degrees Celsius (^o^C), Y axis–measures were performed in baseline (0), 1, 2 and 7 days after surgery; (1)- placebo group, (2)- laser group.

**Fig 11:**
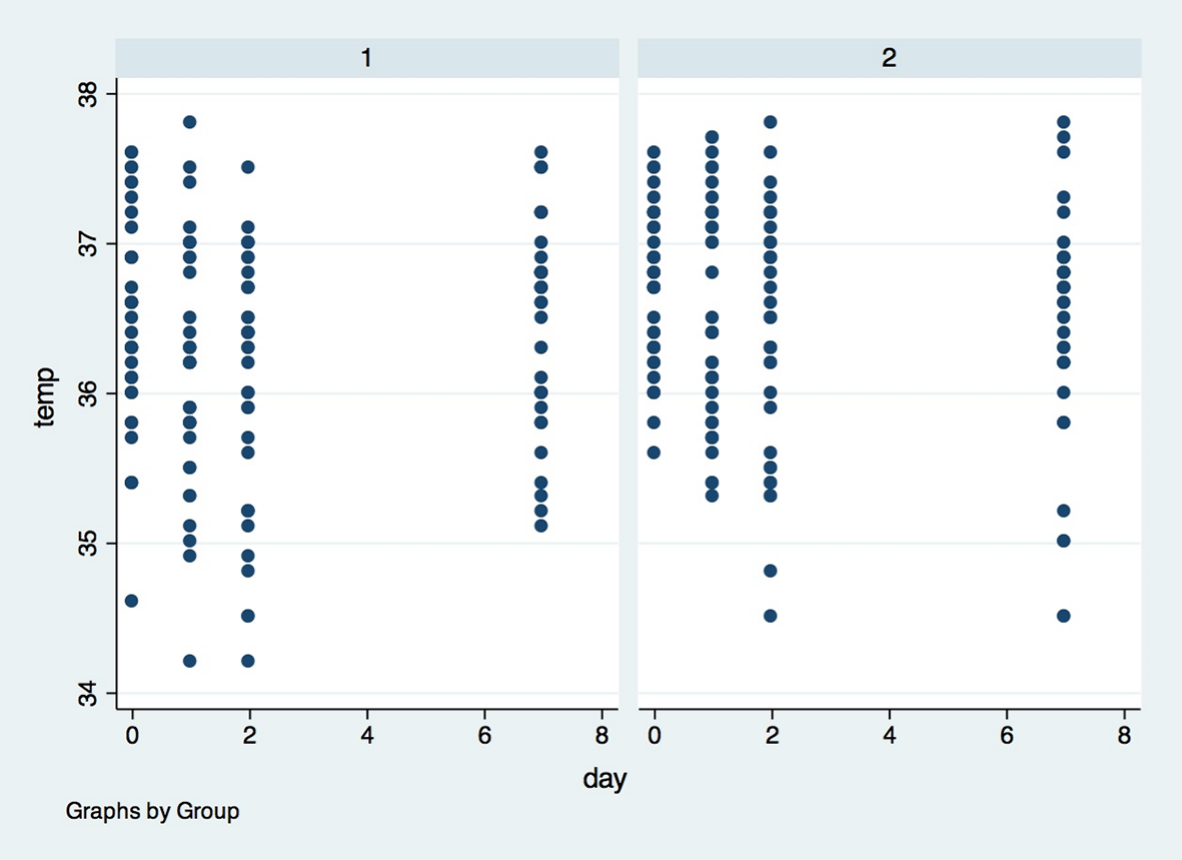
Temperature of opposite side in both groups in the four observed timepoints. X axis–Temperature of opposite side was measured in degrees Celsius (^o^C), Y axis–measures were performed in baseline (0), 1, 2 and 7 days after surgery; (1)- placebo group, (2)- laser group.

Regarding temperature, local temperature on the operated side (Fig 10) and opposite side (Fig 11) were not different (p>0.05) between the groups at baseline, 24 hours after surgery, 48 hours after surgery or 7 days after surgery.

**Fig 12:**
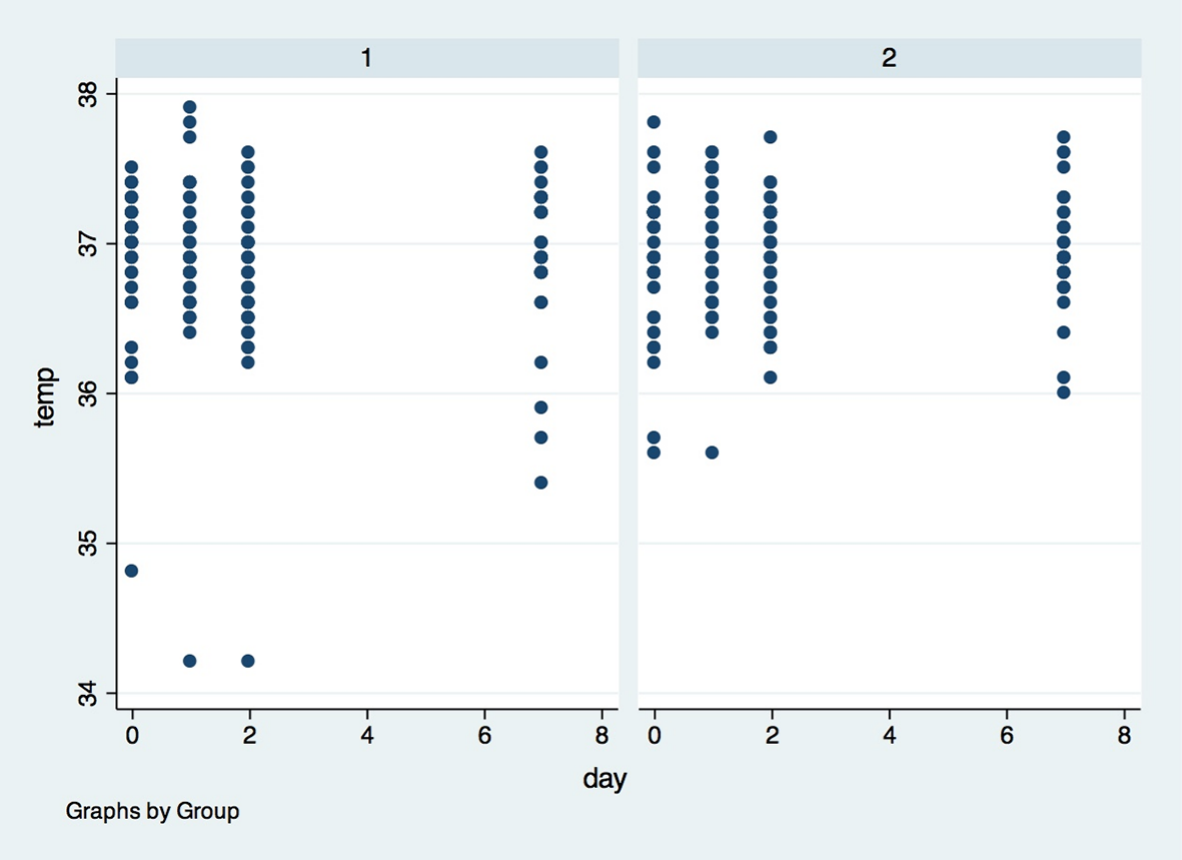
Systemic temperature in both groups in the four observed timepoints. X axis–Sistemic Temperature was measured in degrees Celsius (^o^C), Y axis–measures were performed in baseline (0), 1, 2 and 7 days after surgery; (1)- placebo group, (2)- laser group.

**Fig 13:**
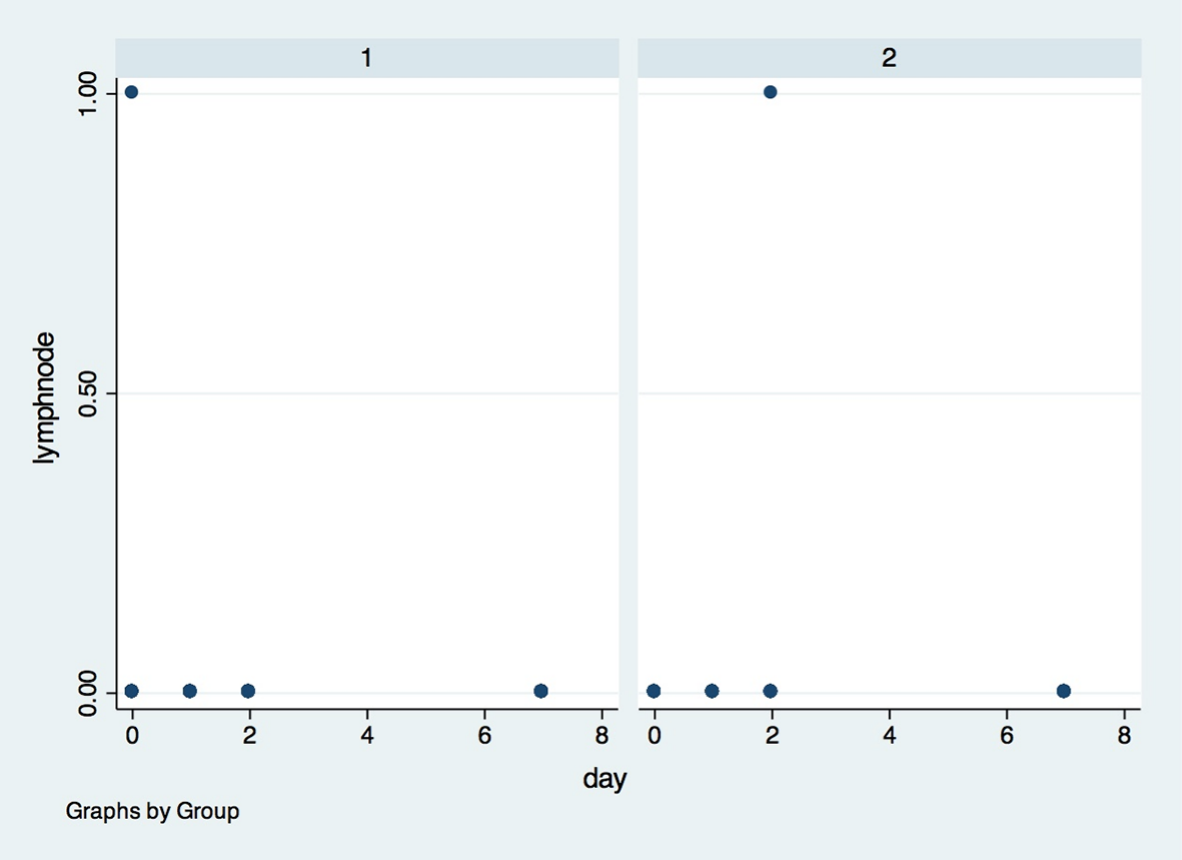
Inflamed lynphonode in both groups in the four observed timepoints. X axis–Number of inflamed lynphonodes, Y axis–measures were performed in baseline (0), 1, 2 and 7 days after surgery; (1)- placebo group, (2)- laser group.

There was also no difference between groups in systemic temperature (Fig 12) and lynphonodes (Fig 13) in the four timepoints (p>0.05).

There were some clinical complications during the study. One of the patients was excluded due to omission of systemic problems during anamnesis. Another patient had a postoperative infection and two others gave up in the postoperative period of the second surgery.

## Discussion

Considering the importance of studying postoperative pain after third molar surgery, this study evaluated a new, alternative treatment to minimize the use of analgesics and anti-inflammatory drugs because many adverse effects have been observed with their use [11,12]. In this study, we observed that LLL treatment applied at auriculotherapy points was neither able to improve postoperative pain nor reduce edema, local temperature or the amount of analgesics required or improve buccal opening. Regarding these variables, we performed a correlation test among the variables for each of the timepoints. The coefficient indicated that the stronger the pain, the smaller the mouth opening. This finding is expected because of exudate that spreads to the muscular tissue area. Third molar removal surgery is considered unpredictable in regard to the level of difficulty; many studies have tried to estimate the difficulty [33] and even experienced surgeons fail to predict the difficulty [34]. The main factors that can influence post-surgical trauma intensity are angulation, patient age, presence of lacerations or divergence, surgical technique, instruments and dental surgeon experience [33–37]. To control for such variability, a single experienced surgeon performed all surgeries using the same surgical technique. The bilateral symmetrical teeth had the same classification (2B of Pell and Gregory), and the patients were between 18 and 28 years of age. We standardized the surgeries to eliminate variables and improve internal validity. However, the average surgery duration was 30 minutes, which may have caused minimal postoperative trauma and minimal inflammation, thus likely making any differences between the two operated sides difficult to notice. Future studies should test the same protocol in longer third molar surgeries because this protocol did not cause difference between the groups in non-traumatic surgeries (mean of 30 minutes).

As an alternative to this protocol, various modalities of auriculotherapy could be tested including auricular acupuncture, auricular electroacupuncture, injection, acupressure, moxibustion, and auricular bloodletting therapy [23]. Auricular acupressure stimulates the auricular points with a noninvasive method that applies pressure using fingers, knuckles, or dull objects such as magnet beads or mustard or vaccaria seeds [37]. Lasers are one of the tools used to stimulate these points [37].

Classical acupuncture is another interesting option to test pain reduction after third molar surgery. Acupuncture can be used in local (e.g., ST6, ST7, and GB20) and distant body points (e.g., LR3). Currently, *Artemisia vulgaris* can be used for several treatments with moxibustion yielding impressive results [38]. *Artemisia vulgaris* extract has anti-inflammatory and antioxidant properties [39]. This plant appears to have unique features. Studies need to be conducted to provide alternatives to analgesics and anti-inflammatories after third molar surgeries.

Regarding the study methodology, rehabilitation of patients was accomplished with a red diode laser at a wavelength of 660 nm (± 10 nm). It has been shown that wavelengths of 650 to 950 nm can penetrate biological tissues up to 3 millimeters [18]. Other authors believe that the ideal wavelength is 633 to 670 nm [19]. Acupuncture requires a wavelength able to reach greater depths in the tissues, since acupuncture needles penetrate from 15 to 70 mm. In auriculotherapy, the stimulus does not reach great depths. In all auriculotherapy modalities, a depth of 1 to 2.5 mm is sufficient. Therefore, the red wavelength (λ = 660 nm) seemed sufficient. The choice of auricular points as well as the laser-related technical parameters, such as wavelength, output power, power density, dose and radiation exposition time, are important and fundamental factors of influence in auriculotherapy [18]. Due to the scarcity of studies on this subject, the choice of dosimetric parameters to perform this work was a challenge. It has been shown that the power density to achieve the same effect of an acupuncture needle should be greater than 1.3 mW/cm^2^ [40] using devices with a small spot. The diameter of the optical fiber of the apparatus used in this study was 600 μm, which corresponded to a spot (area) of 0.002826 cm^2^. Since the amount of energy required for this type of treatment is low, we chose to deliver 1 J at each auriculotherapy point with a power density of 35.4 mW/cm^2^. According to the precepts of Chinese medicine, each individual is a unique being, and an individualized protocol should be developed for each patient. However, to obtain scientific evidence, we had to standardize the interventions to be performed in each patient. Therefore, we sought comprehensive, classically recognized and simple points of application in the daily clinic. The ear points chosen were based on the Chinese school [18] as suggested by Olesson [25] and were simply located by the general practitioner. We chose 6 acupoints based on inflammation-related parameters in general, including pain and edema, and the choice of auricular points was based on studies by several authors [17,18, 26, 41–43]. In this study, we did not intend to perform photobiomodulation in the operated tissue but rather as a stimulus on auriculotherapy points as described by Litscher [16]. The ShenMen point is used in almost all protocols involving auriculotherapy [18,19,26,41–43] and is involved in reducing pain, tension, anxiety, and inflammation as well as supporting other reflex points. The Sympathetic Point regulates autonomic nervous system (ANS) activities, producing sympathetic and parasympathetic balance, and the stomach point has, among other functions, resolution of odontalgias and stress [19,43]. The mandible point is indicated to control pain of the lower teeth, and the adrenal point leads to the release of adrenal hormones, controlling stress and inflammation [25]. To date, few studies have evaluated the action of auriculotherapy in pain control, and the results are difficult to compare given the small number of studies with different approaches and methodologies [17,18,25].

To ensure better methodological quality, this study followed the recommendations of the Standards for Reporting Interventions in Clinical Trials of Acupuncture (STRICTA), which is a new protocol specific to acupuncture studies [44]. To ensure methodological rigor and greater precision in the choice of points, we used a locator that relies on electrical impedance principles. Postoperative pain was the primary endpoint of the study. Therefore, it was very important to measure this parameter correctly. All patients were instructed to use medication only in case of pain. Paracetamol has been prescribed because ethically we could not deprive the patient of the medication. The World Health Organization precept recommended choosing a weak medication first [45,46,47]. Patients were instructed to record the day, time, and intensity of pain at the time they decided to take the medication. In case of severe pain, they were instructed to contact the surgeon and start a codeine+paracetamol regimen. If one does not start with the weak analgesic, one could treat a weak pain with a potent analgesic, which would mask the effect of the proposed treatment and bias the data, as has been reported in some studies [31]. For pain measurement, the visual analogue scale, the main instrument to evaluate the patients’ pain complaint, was used. Graduated in millimeters, this scale allows the use of parametric statistical methods, which improves the accuracy of the data analysis. To blind the study, it was necessary to have the application of a "placebo" laser so that the patient would not know which treatment was effective. To circumvent this bias, the laser was "applied" in the off position. To prevent the patient from noticing any differences, the device activation sound (a beep) was recorded and played at the time of laser application. Thus, the study was blinded to the patient. Related to the treatment side of this intervention, we found no consensus in the literature in treating both sides or a unilateral ear. No controlled randomized clinical trials have compared the effectiveness of both treatments. To standardize the methodology, the application was performed on the right ear for all patients in all surgeries at all study timepoints (immediately after surgery and 24 and 48 h after surgery). Systematic reviews have discussed this topic superficially because there are no randomized controlled studies about this issue [13,37]. Future studies are necessary to test the effectiveness of unilateral or bilateral application.

Finally, although some of the residual plots presented (S1–S11 Figs) deviate from normality, the fitted line shows that there is no clear linear relationship between predicted outcome variables and the residuals. The deviation from normality and presence of outliers is most likely an attribute from the data, given that some variables are binary and other categorical, therefore discrete, not continuous.

A systematic review [10] of 17 randomized controlled trials suggested that auriculotherapy may be effective for the treatment of several types of pain, especially postoperative pain. Another review article [17] suggested that studies in this area have great rigor in the choice of points, type of stimulation, duration of treatment, placebo effect, and patient expectation regarding the treatment outcomes. Another study [48] used electro acupuncture at three acupoints (Shenmen, teeth and mouth) and did not obtain a satisfactory result regarding pain and analgesic consumption after removal of lower third molars using three comparison protocols: electroacupuncture, auriculotherapy and Sham needle (placebo). In our study, we also did not observe any differences between the groups for any variable. We decided to finish the study earlier than expected because we realized that even though only 2 patients were missing, there was no obvious trend towards a difference between the groups. We considered that the two groups behaved identically.

## Conclusion

For this experimental model, low-intensity laser treatment at auriculotherapy points did not prevent postoperative pain in lower third molar surgeries.

## Supporting information

S1 File Ethics Committee in research translated to English. (PDF)

S2 File Original Ethics Committee in research (Portuguese). (PDF)

S3 File Complete clinical protocol sent to the Ethics Committee in research (translated to English). (PDF)

S4 File Original complete clinical Protocol sent to the Ethics Committee in research (Portuguese). (PDF)

S5 File Protocol published in trials (Journal). (PDF)

S6 File CONSORT. (PDF)

S7 File Regression results of the modeling. (PDF)

S8 File Secondary analysis—Mixed effect models. (DOCX)

S1 Fig Postoperative pain—Residuals model. X axis—residuals, Y axis–predicted values.
(JPG)

S2 Fig Presence of edema measured by the corner of the eye to angle of the jaw—Residuals model. X axis—residuals, Y axis–predicted values.
(JPG)

S3 Fig Presence of edema measured by the Tragus to the labial commissure—Residuals model. X axis—residuals, Y axis–predicted values.
(JPG)

S4 Fig Presence of edema Tragus to pogonion—Residuals model. X axis—residuals, Y axis–predicted values.
(JPG)

S5 Fig Mouth opening measurement—Residuals model. X axis—residuals, Y axis–predicted values.
(JPG)

S6 Fig Temperature of operated side—Residuals model. X axis—residuals, Y axis–predicted values.
(JPG)

S7 Fig Temperature of opposite side—Residuals model. X axis—residuals, Y axis–predicted values.
(JPG)

S8 Fig Systemic temperature—Residuals model. X axis—residuals, Y axis–predicted values.
(JPG)

S9 Fig Pain—Fitted values versus residuals and normal probability plot. X axis—residuals, Y axis–Fitted values
(TIFF)

S10 Fig Mouth opened fitted values versus residuals and normal probability plot. X axis—residuals, Y axis–Fitted values.
(TIFF)

S11 Fig Edema fitted values versus residuals and normal probability plot. X axis—residuals, Y axis–Fitted values.
(TIFF)
