# Supplementary material for: Low-level laser treatment applied at auriculotherapy points to reduce postoperative pain in third molar surgery: A randomized, controlled, single-blinded study
Source: PLoS One. 2018 Jun 19;13(6):e0197989. doi: 10.1371/journal.pone.0197989 (PMC6007895; doi:10.1371/journal.pone.0197989)
Supplement: S1 File — (PDF) [file pone.0197989.s001.pdf]

Nove de Julho University - UNINOVE

Consubstantiated Judgement of the Ethics Committee in Research

RESEARCH PROJECT DATA

Title Research: Effectiveness of low-level laser at auriculoterapy points for reducing postoperative pain in lower third molar surgery

Researcher: Anna Carolina Ratto Tempestini Horliana

Subject Area:

Version: 1

CAAE: 45835215.5.0000.5511

Proposing Institution: Nove de Julho University

Main Sponsor: Self-financing.

REVIEW'S DATA

Number of Review: 1.100.869

Date of Review: 06/10/2015

Presentation of the Project:

The need of a comfortable postoperative period and a quick return to daily activities has increased the need to control post-operative inflammation, especially pain and swelling, and whenever possible, the use of anti-inflammatory and analgesic should be minimized. The association between low intensity laser and auricular acupuncture (auriculotherapy) have been shown to be a promising alternative, with low risk of side effects, low cost and indicated for allergic patients or with chronic gastritis.

Objective of Research:

Primary objective:

Evaluate the efficiency of low-intensity laser at auriculotherapy points in reducing postoperative pain in lower third molar surgeries.

Evaluation of Risks and Benefits:

Risks:

Volunteers will undergo only to risks associated to surgery (tooth extraction), and application of local anesthesia. The use of auricular acupuncture associated with low intensity laser presents no risk to your health. We will do our best to reduce these complications by surgical maneuvers least traumatic possible, with the use of medicines and postoperative recommendations, which will be passed to you in writing: mostly liquid or soft diet and cold preferably, rest for a few days and do not proceed mouthwash.

If by chance an infection occurs, immediately, we will delivery a rescue medication (amoxicillin or clindamycin) and if necessary the analgesic acetaminophen (Tylenol ® 500)

so, that is very important your returning on scheduled dates and contact the surgeon by phone if you experience any symptoms. The volunteer may feel some discomfort during the collection of blood (5ml) for the evaluation of systemic inflammatory markers (systemic inflammation), but this discomfort will not be different from that which may occur in any blood collection. If you never did a blood test, you will feel a burning sensation in the area where the needle is inserted. Patients will be clearly informed about the examination and the procedures to be performed.

Benefits:

About the benefits of this research, we may provide a postoperative period with less pain medications, minimizing the potential risks that it could provide to the patient, and a comfortable and painless postoperative period. Moreover, the surgeon will closely monitor you during the most likely period of pain.

Comments and Considerations for Research:

Relevant research, in order to relieve postoperative symptoms in the third molar surgery and may prove to be an important alternative in replacing the systemic medication used in the third molar surgery.

Considerations about Terms of Obligatory Presentation:

Terms are present, including informed consent, all in agreement and no restrictions

Recommendations:

No recommendation

Conclusions or Pendency and Inadequacies:

Appropriate by means of ethical evaluation of research project

No pending

Situation of review:

Approved.

Need Assessment of CONEP:

No

Concluding Remarks at the discretion of the CEP:

São Paulo, June 10, 2015

Assinado por:
Stella Regina Zamuner
(Coordinator)
