# Supplementary material for: Low-level laser treatment applied at auriculotherapy points to reduce postoperative pain in third molar surgery: A randomized, controlled, single-blinded study
Source: PLoS One. 2018 Jun 19;13(6):e0197989. doi: 10.1371/journal.pone.0197989 (PMC6007895; doi:10.1371/journal.pone.0197989)
Supplement: S2 File — (PDF) [file pone.0197989.s002.pdf]

PARECER CONSUBSTANCIADO DO CEP

DADOS DO PROJETO DE PESQUISA

Título da Pesquisa: Eficácia do uso do laser de baixa intensidade nos pontos de auriculoterapia, para redução da dor pós operatória em exodontias de terceiros molares inferiores

Pesquisador: Anna Carolina Ratto Tempestini Horliana

Área Temática:

Versão: 1

CAAE: 45835215.5.0000.5511

Instituição Proponente: ASSOCIACAO EDUCACIONAL NOVE DE JULHO

Patrocinador Principal: Financiamento Próprio

DADOS DO PARECER

Número do Parecer: 1.100.869

Data da Relatoria: 10/06/2015

Apresentação do Projeto:

A necessidade de um pós operatório confortável e rápido retorno às atividades diárias aumentou a necessidade de controlar a inflamação pós-operatória, especialmente a dor e o edema, além disso o uso de anti-inflamatório e analgésicos deve ser minimizado sempre que possível. A associação entre o laser de baixa intensidade e a acupuntura auricular (auriculoterapia) tem se mostrado uma alternativa promissora, com baixo risco de efeitos colaterais, baixo custo e bem indicada para pacientes alérgicos ou com gastrite crônica.

Objetivo da Pesquisa:

Objetivo Primário:

Avaliar a eficácia do laser de baixa intensidade nos pontos de auriculoterapia na redução da dor pós operatória nas exodontias de terceiros molares inferiores.

Avaliação dos Riscos e Benefícios:

Riscos:

Os voluntários estarão sujeitos somente aos riscos associados ao procedimento cirúrgico (extração do dente), com aplicação de anestesia local, já que o uso da acupuntura auricular associado com o laser em baixa intensidade não apresenta risco para a sua saúde. Nós tentaremos diminuir ao

máximo essas complicações através de manobras cirúrgicas o menos traumáticas possíveis, com o uso dos medicamentos e através das recomendações pós-operatórias que serão passadas a você por escrito, principalmente de dieta líquida ou pastosa e de preferência fria, além de repouso por alguns dias, e não fazer bochechos. Se por acaso uma infecção se instalar, imediatamente, iremos administrar um medicamento de
resgate (Amoxicilina ou Clindamicina) além do uso se necessário do analgésico Tylenol ® 500, por isso é muito importante o seu retorno nas datas marcadas, o contato com o cirurgião pelo telefone caso sinta esses sintomas. O voluntário poderá sentir algum desconforto durante a coleta de sangue (5ml) para avaliação dos marcadores inflamatórios sistêmicos (inflamação sistêmica), porém este desconforto não será diferente daquele
que pode ocorrer em qualquer coleta sanguínea. Se nunca foi feito o exame de sangue, sentirá um ardor na região em que a agulha for inserida. Os pacientes serão informados claramente do porquê da realização do exame e dos procedimentos a serem realizados.
Benefícios:
Quanto aos benefícios desta pesquisa, poderemos proporcionar um provável pós-operatório com menor quantidade de medicação analgésica
minimizando assim os potenciais riscos que este tipo de medicação pode proporcionar ao paciente, fazendo com que seu pós operatório seja mais confortável, com menos dor. Além disso você será acompanhado de perto pelo cirurgião que operou você durante o período com maior probabilidade de sentir dor.

Comentários e Considerações sobre a Pesquisa:

Pesquisa relevante afim de aliviar a sintomatologia do pos-operatório na exodontia de terceiros molares, e talvez venha a ser uma alternativa importante na substituição da medicação sistêmica empregada na exodontias de terceiros molares.

Considerações sobre os Termos de apresentação obrigatória:

Termos estão presentes, inclusive o TCLE, todos de acordo e sem restrições.

Recomendações:

Nenhuma recomendação.

Conclusões ou Pendências e Lista de Inadequações:

Projeto adequado segundo avaliação ética de pesquisa.
Sem pendências.

Situação do Parecer:

Aprovado

Necessita Apreciação da CONEP:

Não

Considerações Finais a critério do CEP:

SAO PAULO, 10 de Junho de 2015

Assinado por:
Stella Regina Zamuner
(Coordenador)

Endereço: VERGUEIRO nº 235/249
Bairro: LIBERDADE **CEP:** 01.504-001
UF: SP **Município:** SAO PAULO
Telefone: (11)3385-9197 **E-mail:** comitedeetica@uninove.br
