# Supplementary material for: Low-level laser treatment applied at auriculotherapy points to reduce postoperative pain in third molar surgery: A randomized, controlled, single-blinded study
Source: PLoS One. 2018 Jun 19;13(6):e0197989. doi: 10.1371/journal.pone.0197989 (PMC6007895; doi:10.1371/journal.pone.0197989)
Supplement: S3 File — (PDF) [file pone.0197989.s003.pdf]

**Title of project:** Efficacy of low-intensity laser in auriculo-therapy points, in reduction of postoperative pain in lower third molar surgery

**Master student:** Helio Sampaio Filho[4]

**Advisor:** Anna Carolina Ratto Tempestini Horliana[1]

**Researchers participating in the project:**

- Profa. Dra Kristianne Porta Santos Fernandes[1,2]
- Profa. Dra Raquel Agnelli Mesquita Ferrari[1,2]
- Profa. Dra Sandra Kalil Bussadori [1,2]
- Profa. Dra Daniela de Fátima Teixeira da Silva[1]
- Juliane Sotto Ramos [5]
- Danielly da Silva Lima[5]

[1]Professor, Postgraduate Program in Biophotonics applied to Health Sciences, Universidade Nove de Julho / UNINOVE

[2]Professor, Postgraduate Program in Rehabilitation, Universidade Nove de Julho / UNINOVE

[4]Master degree Student, Postgraduate Program in Biophotonics applied to Health Sciences, Universidade Nove de Julho / UNINOVE

[5] Undergraduate student, College of Nove de Julho University / UNINOVE

**Address of the Coordinating Center:** Universidade Nove de Julho (UNINOVE) Av. Vergueiro, 235/249 – Liberdade, São Paulo – SP

The phenomena related to the removal of third molars such as pain, edema and trismus are presented in practically all the patients that need surgical procedure. This model has been widely used to evaluate the therapeutic options, as the use of anti-Inflammatory and analgesics associated or not with the application of Low Level Laser Therapy (LLLT). The necessity of a comfortable postoperative recovery and rapid return to daily activities increased the neecessity of control post-operative inflammation, especially pain and edema. The use of anti-inflammatory and analgesics are not risk-free and should be minimized whenever possible. The association between low level laser and Auricular Acupuncture (Auriculotherapy) has been shown to be a promising alternative, with low risk of side effects, low cost and well indicated for patients allergic or with

chronic gastritis. The objective of this study will be to evaluate the efficacy of the low intensity laser at the auriculotherapy points on the postoperative pain reduction of in lower third molar surgeries . A sample of 40 healthy patients of both genders ages between 18 and 30 years with the neecessity to remove the bilateral included third third molars, classificated by Pell & Gregory and screened at the Dentistry Clinic of the Faculty of Dentistry of Nove de Julho University, UNINOVE, after approval by the Committee of Ethics in Research, who will be invited after signing the terms of consente

**Introdução e Justificativa do Estudo**

The postoperative period in third molar extractions is usually accompanied by pain and edema, and its control is essential for both the patient and the dental surgeon. There is usually an inflammatory reaction followed by pain, edema and trismus, thus decreasing the subjects' quality of life during the first three postoperative days (Pouchain et al.2015; Ferrante et al 2103; Markovic et al 2006)

According to Zuniga et al. (2004), about 63% of the patients feel intense pain during the first postoperative day, most intense 3 to 5 hours after the end of the anesthesia (Markovic, 2006).

Anti-inflammatory drugs are usually prescribed preemptively and also postoperatively, with the most indicated drugs being corticosteroids and non-steroidal anti-inflammatory drugs (Simone et al., 2013).

However, some studies have demonstrated adverse reactions to non-steroidal anti-inflammatory drugs such as gastrointestinal disorders (erosions, ulcers, dyspepsia) with serious hemorrhagic complications, increaseing cardiovascular complications, renal insufficiency and platelet abnormalities (Han 2014; Ramin , et al 2013, Wilcox 1997).

Among the main local complications of the exodontia we can mention alveolar osteitis (dry alveolitis), edema, trismus abscess and pain, and among systemic complications fever, and alteration of lymph nodes. The majority of studies (Poeschl et al 2004, Siddiqi, 2010, Kaczmarzyk T, 2009, Monaco, 2009)

that assessing the clinical efficacy of antibiotics use these infection parameters as study variables

The need for a comfortable postoperative recovery and a rapid return to daily activities increased the importance of controlling post-operative inflammation, especially with regard to pain and edema. Surgery for the removal of third molars is considered the gold standard in pain studies (Zuniga et al-2004;)

It has become very common because of its high local nociceptive sensitivity and the fact that it is usually indicated for young patients whse normally do not take medication regularly. In most cases, very similar procedures are performed on the same patient, one on each side of the jaw, so that the individual can have their own control (split mouth), elimination of possible individual differences in the inflammatory response and pain threshold .

Currently the control of pain and inflammation is performed with the association of a corticosteroid and a non-steroidal anti-inflammatory (Han JB et al 2014) Today, a new alternative has emerged with interesting results, the low intensity laser can be used to minimize these effects. (Markovic AB, Todorovic L 2006). Studies have shown promising possibilities for the modulation of phenomena related to inflammation, mainly pain, edema and trismus: auricular acupuncture and low intensity laser.

**Auriculopuncture or Auricular Acupuncture**

Acupuncture, an integral part of a system called Traditional Chinese Medicine (TCM) that consists on the needles application at certain points distributed throughout the body surface. It has been used for many years in the most diverse areas of Health, and more and more, it has been reason for new studies. (...).

One of the modalities of acupuncture is auriculotherapy, which, in addition to the traditional use of needles, nowadays has other types of stimuli, such as auricular electroacupuncture, laser auriculotherapy, among others (Hising, C et al., 2104, Round, R Et al 2013). Auricular acupuncture can be defined as a system of diagnosis and treatment based on the normalization of organ dysfunctions through the stimulation of localized points in the ear (Gori, Firenzuol, 2007), or a therapeutic intervention in which stimuli in the outer ear are used to alleviate health conditions in various parts of the body (Oleson, 2003)

There are two schools that developed the study and application of auriculotherapy that according to Round et al (2013) are divided into *old auricular acupuncture and modern auricular acupuncture*, the first one being based on the stimulation of auricular acupuncture points

that would be connected to the meridians and used basically for the resolution of pain and the second is Paul Nogier's school, which presuppose that a somatotropic organization of the body is represented in the human ear (Round, R et al 2013)

Another division of the terms cited above, according to the same authors, include the Chinese Atrial Acupuncture System and the European Atrial Acupuncture System in addition to the development for auriculotherapy and auricle medicine

There are several propositions of the theoretical bases of Auriculotherapy such as neurological theory, embryological theory, microsystems theory, the Chinese Traditional Medicine and Hormonal Basis energy theory to try to understand the mechanisms of action of the same (Oleson, 2003).

**Application of laser therapy in the postoperative period and its advantages**

Laser, acronym for Ligth Amplification by Stimulated Emission of Radiation) has been used and studied in the last 50 years in the most diverse areas, and has become an essential tool with applications to the most diverse areas, among them in environmental monitoring, precision metrology, biological decontamination, transmission and storage of data and in the treatments in Health.

Therefore Low Level Laser (LLL) is the application of electromagnetic radiation in a biological system that promotes tissue regeneration, reduction of inflammation and pain relief (Shirin F, 2014).

Several studies have shown the effectiveness of the low intensity laser in the modulation and control of postoperative pain in oral surgery in general and in third molar surgery (He et al - 2014, Markovic et al., 2006, Saber 2012).

The use of laser in postoperative periuod of impacted third molar surgery demonstrates a decreased pain, edema and trismus when associated with the use of anti-inflammatory drugs (Amarillas-Escobar 2010), although other studies demonstrates only an improvement in relation to the oral opening after surgery (Brignardello-Petersen et al-2012) as well as only for trismus and edema (Ferrante M et all - 2012) To date, there are no randomized controlled trials that demonstrate the efficacy of low intensity LASER for pain control, postoperative edema and inflammation.

**General objective**

To evaluate the efficacy of low level laser at the auriculotherapy points in the reduction of postoperative pain in lower third molars surgeries.

**Null hypothesis**

The low intensity laser used in auriculotherapy points is not able to reduce postoperative pain after lower third molar surgeries.

**Experimental hypothesis**

The low intensity laser used in auriculotherapy points is able to reduce postoperative pain after lower third molar surgeries.

## Methods

After verbal and written explanation of the study, the patients who agree to participate will sign the Informed Consent Term (ICT) after approval of the Research Ethics Committee of the University of Nove de Julho (UNINOVE). The study will be in accordance with the Declaration of Helsinki (revised in Fortaleza, 2013). The sample will consist of 60 healthy patients of both genders aged 18 to 28 years with surgical removal of lower third molars. The mollars need to be bilateral and symmetrical. Two surgeries will be performed in the same patient, that is, a split-mouth study. Surgeries will be performed at the Dental Clinic of the University of Nove de Julho - UNINOVE in the São Paulo city, Brazil, in the period of August 2015 to August 2016.

## Exclusion Criteria

Patients will be excluded:

- Allergic to any drug used in the research (amoxicillin, paracetamol, chlorhexidine 2%),
- Pregnant or breastfeeding women,

• Smokers,

• Subjected to radiotherapy in the head and neck region,

• Systemic or local infection (eg pericoronitis or periodontal abscess),

• Radiolucent lesions or images, associated with the teeth to be extracted,

• Who have used anti-inflammatory or antibiotic in the last 3 months,

• Patients who present any type of complication during surgery (eg hemorrhage, operative difficulty, time greater than 90 minutes of surgery), since these cases differ from the standard expected for third molar surgeries. In these cases the central analgesic will be prescribed and the data collected for this patient will not be included in the research.

• Who used the medications differently from the way they were prescribed

**Inclusion criteria**

Patients will be included in the study:

• With teeth in position II B, according to the classification of Pell and Gregory (Figure 1 and Table 1).

• With indication for extraction of the third molars (recurrent infections, bad position, orthodontic indication) or professional indication presented in writing.

• ASA I (negative medical history)

• Systolic blood pressure less than 140 mmHg and diastolic blood pressure less than 90 mmHg and heart rate with values 70 ± 20 beats / minute.

Table 1. Classification of Pell and Gregory (1933)

| Pell e Gregory classification | |
|---|---|
| Class I | There is sufficient space between the branch and the distal of the second molar for the accommodation of the distal mesio diameter of the third molar |
| Class II | The space between the second molar and the branch of the mandible is smaller than the distal mesio diameter of the third molar |
| Class III | The third molar is in the branch of the mandible |
| A | The highest portion of the third molar is above the occlusal plane. |
| B | The highest portion of the third molar lies between the occlusal plane and the cervical line of the second molar |
| C | The highest portion of the third molar is below the cervical line of the second molar |

Figura 1 - Radiografia panorâmica mostrando disposição anatômica semelhante bilateral e Classificação 2B.

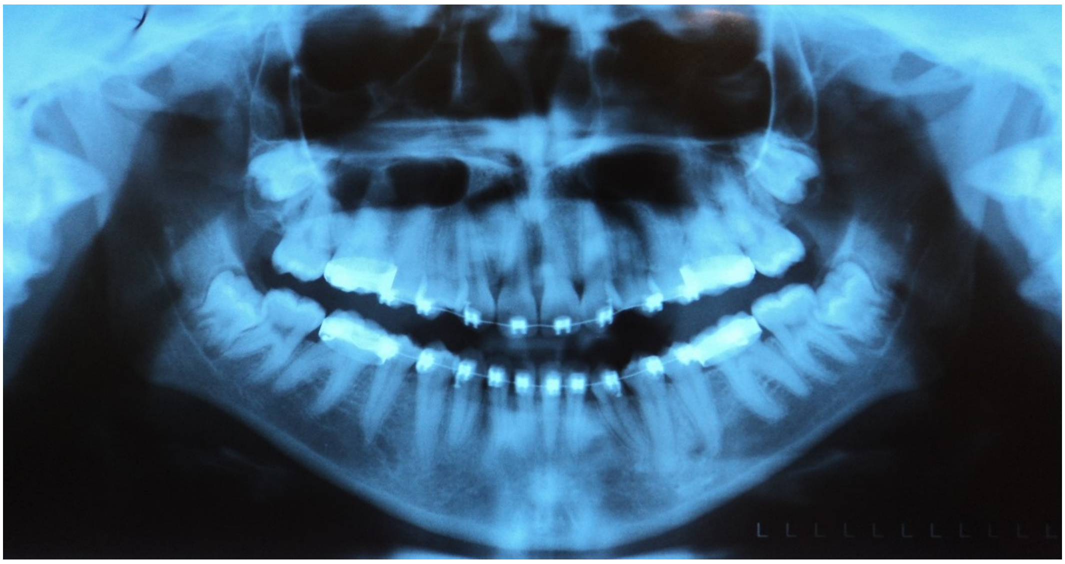

**Calculation of sample size**

The sample size will be 60 patients. This value was estimated based on preliminary studies and was calculated to provide 90% strength ($\alpha = 0.05$).

**Examiner Calibration Training and Evaluation**

Intra-examiner agrément

An examiner (gold standard) will perform the calibration exercise in order to achieve maximum reproducibility of the measurements performed. Ten patients who will not be included in the study will be evaluated. Evaluations will be made for calibration of measures of facial edema, mouth opening and visual analog scale. The intraclass correlation coefficient (ICC) will be calculated in order to evaluate the intra-examiner agreement $\geq$ 0.90 in relation to the clinical parameters cited (continuous measures). Digital caliper and flexible millimeter will be used. Patients will be submitted to periodontal clinical examination by a previously calibrated examiner, in order to realize the diagnosis. This evaluation will be done at the beginning of the study.

**Randomization, blinding and group composition**

To randomly distribute the order of the operated sides, a random with 40 pairs of numbers (1 = Experimental group and 2 = Control group) will be performed through the program Microsoft Excel, version 2013. As the numbers are drawn, they will be placed inside envelopes Identified with sequential numbers according to the order obtained in the draw. The envelopes will be sealed and will remain sealed in the same numerical order in a safe place until the surgery is performed. The drawing and preparation of the envelopes will be performed by a person not involved in the study.

Patients who seek for Dental Clinic of the University of Nove de Julho - UNINOVE in the city of São Paulo from August 2015 to August 2016 that fit the inclusion criteria will be invited to participate of this study. It is expected to screen approximately 200 patients. Immediately before the exodontia the surgeon (H.S.) will remove and open 1 envelope (without changing the numerical sequence of the other envelopes) and will perform the indicated procedure.

The blinding will be done in a way that neither the patient nor the surgeon will know which treatment will be performed (double-blind study). The only person who will know the treatment performed will be the researcher responsible for the application of LLL.

The experimental design will consist of 2 groups that will receive different treatments for split mouth:

**Group 1** (Experimental) (n = 60 surgeries) - Surgeries will be performed in conventional manner and patients will receive low level laser at specific auriculo-therapy points for pain prevention and modulation of postoperative inflammation in the immediate postoperative period.

**Group 2** (Control) (n = 60 surgeries) - Surgeries will be performed in a conventional way, and patients will receive low level laser off at specific auriculotherapy points for pain prevention and modulation of postoperative inflammation in the immediate postoperative period.

For all patients will be given paracetamol 750mg to use in case of pain. A prescription will be provided for Paracetamol with Codeine Phosphate 30 mg Tylex® Janssen-Cilag if the patient feels very strong pain. Data from these patients will not be included in the study, without prejudice to the patient's treatment.

**Anamnesis**

In the anamnesis, beyond the traditional questions related to the patient's general health, it will be collected demographic data (age, sex, marital status, occupation, educational level, living conditions, salary), medical history data (main complaint, current illness status, Medical history and medications) habits and addictions (smoking, alcoholism)

**Analysis of the oral health impact profile (OHIP-14 Questionnaire)**

The Oral Health Impact Profile (OHIP-14) is a simplified form of the original OHIP questionnaire, used to evaluate the impact of oral health on the quality of life of the research subjects (De Oliveira and Nadanovsky, 2005). This questionnaire is indicated to evaluate the quality of life during the postoperative period of patients submitted to third molar extraction (Negreiros, 2012). The items are distributed among the following subscales: functional limitation,

pain, psychological discomfort, physical disability, psychological deficiency, social incapacity and disability.
This evaluation will be performed on the day of the anamnesis (baseline), 1,2, 3 and 7 days always by the same evaluator calibrated at the beginning of the study to complete the questionnaire

**Verification of vital signs**

Vital signs such as heart rate, blood pressure, and body temperature will be measured at all study visits. Blood pressure will be measured using Omron-75 (HEM-759-E [EU]) under a seated position after 10 minutes of rest. For the calculation of the body mass index (BMI), height and weight will be assessed during the screening visit.

**Surgical technique**

All patients will be operated with the same surgical technique, since the degree of difficulty will be standardized by the classification of Pell and Gregory, 1933 and always by the same specialist operator in maxillofacial surgery (H.S.). There will be 21 days of whashout between the sides operated.
All patients will undergo the following surgical maneuvers:

- - Extra-oral antisepsis with Chlorhexidine 2%
- • - Intraoral oral antisepsis with Periogard®
- • - Placement of sterile surgical materials
- • - Blockade of Inferior alveolar nerve
- • - Blockade of the buccal nerve
- • Incision will be performedwith a periostal mucus flap extending from the mandibular ramus to the distal first molar where another incision will be performed
- • - Displacement of the surgical flap
- • Osteotomy in the vestibular and distal área arpond the inferior third molar with the same thickness as the drill and sufficient depth to reach the bone marrow
- • - Odontosection of the medial portion of the crown
-

- • - Dislocation and avulsion of teeth with elevators
- • - Irrigation with saline
- • - Suture

**Methodology for application of Auriculopuncture points –**

The operator will initially mark the points to be used in the external auricle of the patient to be operated with a red gel pen.

The following points will be chosen for the application of LLTT in the chosen auricle, based on OLESON, T (1996):

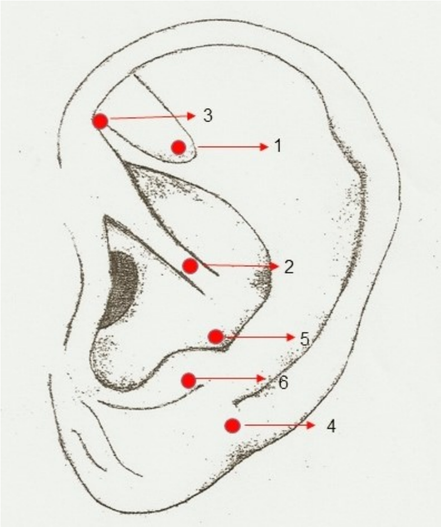


1) Shen Men
2) Zero Point
3) Autonomic (Sympathetic)
4) Lower jaw
5) Tootache 3
6) Sub córtex

**Justification for the choice of points of auriculo puntura**

The World Federation of Acupuncture-Moxabustion Societies (WFAS), due to the fact that there are numerous nomenclatures and maps of Auriculotherapy used in several countries, established some norms for the standardization of atrial acupuncture points (Wang L et al 2013). Promoted the so-called ISAAPs (International standart of auricular acupuncture points). The published nomenclature and location was based on the subdivision and models proposed by Oleson (OLESON, T - 1996).

Based on these data, the points to be used in this study will be those listed above whose functions are:

1) Shen Men -

Main function: relieve stress, pain, tension, anxiety, inflammatory diseases.

2) Zero Point

Main function - assists in the balance of homeostasis, serves as support for the maintenance of other points

3) Autonomic (Chinese Sympathetic point)

Main function - reduction of neuro-vegetative imbalance

4) Lower jaw -

Main Function - Pain Relief of Lower Teeth

5) Tootache 3

Main function - Relief of general tooth pain

6) Sub cortex

Main function - Reduction of chronic pain

After marking the standardized points, the application of the Laser will be performed by the assistant who will obey the random choice (if the patient belongs to Group 1 or 2) already described, without contact of the surgeon in this choice.

At the end of the surgery, the predicted immediate postoperative evaluation (TPO1) will be performed.

**Low-intensity Laser Specifications**

The red diode laser (Therapy XT®), wavelength 660 nm (± 10nm) will be used.

**Medications**

- - Amoxilicillin capsule 500mg Novocilin® Ache
- - Chlorhexidine Gluconate 0.12% Periogard® Colgate Spray
- - Paracetamol tablet 750 mg Paracetamol® Janssen-Cilag
- - Mepivacaine 2% with adrenaline 1: 100,000 Mepiadre® DFL

**Post-treatment assessments**

All patients will receive evaluations of postoperative pain, edema, mouth opening, lymphadenopathy, dysphagia, amount of analgesics ingested, fever, local presence of infection and quality of life. These study variables will be assessed at baseline (before starting the study), after 24 hours, after 36 hours and after 48 hours and then after 1 week when the patient comes to remove the suture. The second evaluation will be done in the same way and in the same order when the second surgery is performed.

Blood samples from all patients will be collected to evaluate inflammatory cytokines in serum at baseline and 24 hours after surgery (according to the item "Plasma levels of inflammatory markers").

**Clinical variables**

For the evaluation of postoperative pain, the clinical signs (study variables) will be analyzed in the time immediately after the end of the surgery (TPO1), after 48 hours (TPO2), on the seventh day after surgery in which the suture removal (TPO7). The second evaluation will be done in the same way and in the same order when the second surgery is performed. The main signs and symptoms that characterize the infectious condition will be analyzed: presence of trismus, edema, postoperative pain, dysphagia, fever, palpation of lymph nodes and evaluation of any infectious condition.

For the evaluation of the presence of trismus will be used the inter-incisal measurement with a digital caliper. During the clinical examination (performed one week before the experiment), the distance between the incisal edge of the upper and lower central incisor (Siddiqi, 2010) will be asked to perform the maxi- mum mouth opening, when measured in millimeters. Only patients with upper and lower central incisors will be admitted. This measure will be obtained preoperatively, considered as basal value (Basal) and in the postoperative periods already described

To evaluate edema, the measurement method described by Neupert will be used; Lee; Philput. (1992). Measurements will be made through linear distances between the angle of the mandible and the following points: lower tip of tragus, lower portion of wing of nose, outer corner of eye, commissure of lip and ment (midline). Measurements will be made using a flexible ruler and the markings of the points will be done with a

demographic pen. The angle of the jaw will be permanent from the preoperative period (baseline) so that the measurements can be standardized.

The pain will be assessed by applying a visual analog scale (VAS), which consists of a line of 100 mm, numbered every centimeter, and with both ends closed. One end is marked "0" and the other "100" which means without pain and unbearable pain respectively. The instructions of pain assessment will be given by the researcher. Each patient will be instructed to mark with a vertical trace the point that best corresponds to the intensity of pain at the time of evaluation (Mark, 1986). The pain will be evaluated on the same postoperative days as the other variables evaluated by the same dental professional (days scheduled for return) and by the patient at the following times: 6 hours after surgery, at night (between 22: 00- 23:00 hs) and for the following 6 days - in the morning (between 7:00 a.m. to 9:00 p.m.) and in the evening (between 22:00 a.m. to 23:00 p.m.)

I-----------------------------------------------------------------------------------------I

0 100mm

The evaluation of dysphagia will be performed through a numerical scale in which 0 points will indicate total absence of dysphagia; 1 point, dysphagia to solid foods only and 2 points, dysphagia to any liquid or solid food.

Evaluation of inflammatory markers

Plasma collections

Blood samples will be obtained by venipuncture performed by a technician trained in the baseline and 48 hours after the exodontia. Samples will be stored at -80 ° C until use. Leukocyte counting and determination of plasma levels of TNF-α, CRP, IL1-α and IL1-β, IL-6 and IL-8 factorial inflammatory markers by ELISA using commercial kits (Peprotech Inc., Rocky Hill, NJ, USA) according to the manufacturer's instructions. Samples with blood will be discarded and the material will be stored in a dry tube at -80oC until use.

## References


Adel Al-Asfour Postoperative Infection after Surgical Removal of Impacted Mandibular Third Molars:An Analysis of 110 Consecutive Procedures Med Princ Pract 2009;18:48–52

Amarillas-Escobar E D - Use of Therapeutic Laser After Surgical Removal of Impacted Lower Third Molars - American Association of Oral and Maxillofacial Surgeons J Oral Maxillofac Surg 68:319-324, 2010

Ataoglu H; Oz GY; Candirli C; Kiziloglu D.Routine antibiotic prophylaxis is not necessary during operations to remove third molars.British Journal of Oral and Maxillofacial Surg. 2008;46: 133–5.

Babatunde, O B et al Prospective, Randomized, Open-Label, Pilot
Bergdahl L. Hedstr¨om 2004 Metronidazole for the prevention of dry socket after removal of partially impacted mandibular third molar: a randomised controlled trial British Journal of Oral and Maxillofacial Surgery (2004) 42, 555—558

Cho S, Ki Y, Chu V, Chan J. Impaction of permanent Mandibular Second Molars in Ethnic Chinese schoolchildrenJCDA • www.cda-adc.ca/jcda • July/August 2008, Vol. 74, No. 6

Clauser B, Barone R, Briccoli L, Baleani A. Complications in surgical removal of mandibular third molars.Minerva Stomatol. 2009 Jul-Aug;58(7-8):359-66.

Clauser B, Barone R, Briccoli L, Baleani A.Complications in surgical removal of mandibular third molars. Minerva Stomatol. 2009 Jul-Aug;58(7-8):359-66.

Clinical Trial Comparing the Effects of Dexamethasone Coadministered with Diclofenac Potassium or Acetaminophen and Diclofenac Potassium Monotherapy After Third-Molar Extraction in Adults, currente Therapeutic Research, 2006 Jul/Aug 67(4)

Doeuk,C et all - Current indications for low level laser treatment inmaxillofacial surgery: a review- - British Journal of Oral and Maxillofacial Surgery 53 (2015) 309–315

Epstein JB, Chong S, Le ND. A survey of antibiotic use in dentistry. Journal of the American Dental Association 2000;131:1600-9.

Ferrante, M - Effect of low-level laser therapy after extraction of impacted lower third molars - Lasers Med Sci (2013) 28:845–849

Haas DA, Epstein JB, Eggert FM. Antimicrobial resistance: dentistry's role. Journal of the Canadian Dental Association 1998;64:496-502.

Han JB et a Postoperative gastrointestinal bleeding in orthognathic surgery patients: its estimated prevalence and possible association to known risk factors.J Oral Maxillofac Surg, 2014 Oct;72(10):2043-51. doi: 10.1016/j.joms.2014.02.039. Epub 2014 Mar 13.

He WL , Yu FY, Pan J, Zhuang R, Duam PJ - A systematic review and meta-analysis on the efficacy of low-level laser therapy in the management of complication after mandibular third molar surgery. 2014- Lasers Med Sci

Hersh EV. Adverse drug interactions in dental practice: interactions involving antibiotics. Journal of the American Dental Association 1999;130:236-51.

Huovinen P, Cars O Control of antimicrobial resistance: time for action BMJ 1998; 317 : 6131998

Jaafar N, Nor GM. The prevalence of post-extraction complications in an outpatient dental clinic in Kuala Lumpur Malaysia--a retrospective survey. Singapore Dent J. 2000 Feb;23(1):24-8.

Jensen M.P, Karoly P, Braver S. The measurement of clinical pain intensity: a comparison of six methods. *Pain.* 1986;27:117–126.

Kaczmarzyk T, Wichlinski J, Stypulkowska J , Zaleska M, Panas M, Woron J.Single-dose and multi-dose clindamycin therapy fails to demonstrate efficacy in preventing infectious and inflammatory complications in third molar surgery. Int. J.Oral Maxillofac. Surg. 2007; 36: 417–422.

Kim T W, Årtun J, Behbehani F, Artese F Prevalence of third molar impaction in orthodontic patients treated nonextraction and with extraction of 4 premolars American Journal of Orthodontics and Dentofacial Orthopedics Volume 123, Number 2

Lawler B, Sambrook PJ, Goss AN. Antibiotic prophylaxis for dentoalveolar surgery: is it indicated? Aust Dent J. 2005 Dec;50(4 Suppl 2):S54-9.

Lodi G, Sardella A, Bez C, Demarosi F, Carrassi A. Antibiotics to prevent complications following tooth extractions (Protocol for a Cochrane Review). In: The Cochrane Library, Issue 1, 2010.

Louise C. Sweeney1, Jayshree Dave1,2, Philip A. Chambers3 and John Heritage1* Antibiotic resistance in general dental practice—a cause for concern? Journal of Antimicrobial Chemotherapy (2004) 53, 567–576

Malamed SF. Handbook of local anesthesia. 5nd ed. St Louis: Mosby-Year Book; 2005

Mark P. Jensena, Paul Karoly, a and Sanford Bravera The measurement of clinical pain intensity: a comparison of six methods Pain Volume 27, Issue 1, October 1986, Pages 117-126

Markovic AB, Todorovic L , Postoperative analgesia after lower third molar surgery: contribution of the use of long-acting local anesthetics, low-power laser, and diclofenac. Oral Surg, Oral Med Oral Pathol Oral Radio Endod 2006 Nov;102(5):e4-8. Epub 2006 Aug 10.

Mehrabi M, Allen JM, Roser SM.Therapeutic agents in perioperative third molar surgical procedures. Oral Maxillofac Surg Clin North Am. 2007 Feb;19(1):69-84,

Monaco G, Tavernese L, Agostini R, Marchetti C. Evaluation of antibiotic prophylaxis in reducing postoperative infection after mandibular third molar extraction in young patients. J Oral Maxillofac Surg. 2009 Jul;67(7):1467-72.

Neupert EA, Lee JW, Philput CB. Evatuation of dexamethasone for reduction of postsurgical sequelae of third molar removal. J Oral Maxillofac Surg. 1992; 50:1177-83

Nogueira AS, Ponzoni D, Pasinato E, Ferrari LK, FariasRD. Principais Transtornos Ocasionados por Dentes Inclusos. Rev APCD. 1997; 51: 247-9.

Paschoal M A B et all - Therapeutic Effects of Low-Level Laser Therapy After Premolar Extraction in Adolescents: A Randomized Double-Blind Clinical Trial - Photomedicine and Laser Surgery Volume 30, Number 9, 2012

Paulo RSMF, Marta RP, Thiago SS, Silva LCF, Lélia BS Evaluation of prevalence of pathologic conditions in impacted wisdom Teeth Rev. Cir. Traumatol. Buco-Maxilo-fac., Camaragibe v.8, n.3, p. 41 - 48, jul./set. 2008

Pell GJ, Gregory BT. Impacted mandibular third molars: Classification and modified techniques for removal. Dent Digest 1933; 39:330-8.

Peterson LJ: Antibiotic prophylaxis against wound infections in oral and maxillofacial surgery. J Oral Maxillofac Surg. 1990; 48:617.

Peterson LJ: Princípios do tratamento de dentes impactados In: Peterson LJ; Ellis E; Hupp JR; Tucker M.R: Cirurgia oral e maxilofacial contemporânea. 4ª Ed. Rio de Janeiro: Mosby; 2005. p.197-226.

Peterson, L.J.; Ellis, E.; Hupp, J.R.; Tucker, M.R. - Cirurgia Oral e Maxilofacial

Poeschl PW, Eckel D, Poeschl E. Postoperative prophylactic antibiotic treatment in third molar surgery — a necessity? J Oral Maxillofac Surg 2004; 62:3–8.

Pouchain E.C., Comparative efficacy of nimesulide and ketoprofen on inflammatory events in third molar surgery: a Split-mouth, prospective, randomized, double-bilnd study. Int. J Oral maxillofac surg 2015 apr

Quek SL, Tay CK, Tay KH, Toh SL, Lim KC. Pattern of third molar impaction in a Singapore Chinese population: a retrospective radiographic survey. Int J Oral Maxillofac Surg. 2003 Oct;32(5):548-52.

Rabischong P, Terral C, MD Scientific Basis of Auriculotherapy: State of the Art – Medical Acupuncture Volume 26, Number 2, 2014

Ramin M, et al - Lower gastrointestinal adverse effects of NSAIDS: an extreme example of a common problem - BMJ Case Rep 2013. doi:10.1136/bcr-2012-008274

Saber K , Chiniforush L, Shahabi S - The effect of low level laser therapy on pain reduction after third molar surgery.; Minerva Stomatol 2012 Jul-Aug;61(7-8):319-22.

Sekhar C, Narayanan V, Baig M. Role of antimicrobials in third molar surgery: prospective, double blind, randomized, placebo-controlled clinical study. British Journal of Oral and Maxillofacial Surgery 2001;31:134–7

Shirin F et al - Biological Effects of Low Level Laser Therapy – 2014 – J Lasers Med Sci 5 ( 2 ) 58-62

SiddiqiA, Morkel JA, Zafar Z. Antibiotic prophylaxis in third molar surgery: A randomized double-blind placebo-controlled clinical trial using split-mouth techniqueA Int. J. Oral Maxillofac. Surg. 2010; 39: 107–114 .

Silveira HM, Ramos JR JWN, Pereira RA. Profilaxia antibiótica para remoção de terceiros molares. Rev Bras Odontolol . 2003;60(3):188-191.

**Sweeney LC, Dave J, Chambers PA, Heritage J** Antibiotic resistance in general dental practice—a cause for concern? Journal of Antimicrobial Chemotherapy (2004) 53, 567-576

Thomas DW, Hill CM. An audit of antibiotic prescribing in third molar surgery.Br J Oral Maxillofac Surg 1997; 35:126–128.

Wang L et all - Status and strategies analysis on international standardization of auricular acupuncture points- Tradit Chin Med 2013 June15;33(3):408-412

Wilcox CM, Alexander LN, Cotsonis GA, et al. Nonsteroidal anti-inflammatory drugs are associated with both upper and lower gastrointestinal bleeding. Dig Dis Sci 1997;42:990–7.

Zuniga Jr et all, Analgesic safety and efficacy of diclofenac sodium softgels onpostoperative third molar extraction pain – J Oral Maxillofac Surg 2004; Jul;62(7):806-15

Yamalik K, Bozkaya S. The predictivity of mandibular position as a risk indicatior for pericoronitis. Clin Oral Inv. 2007; in press.
