# Supplementary material for: Low-level laser treatment applied at auriculotherapy points to reduce postoperative pain in third molar surgery: A randomized, controlled, single-blinded study
Source: PLoS One. 2018 Jun 19;13(6):e0197989. doi: 10.1371/journal.pone.0197989 (PMC6007895; doi:10.1371/journal.pone.0197989)
Supplement: S4 File — (PDF) [file pone.0197989.s004.pdf]

Título do projeto: Eficácia do uso do laser de baixa intensidade nos pontos de Auriculoterapia, na redução da dor pós operatória em exodontias de terceiros molares inferiores

Mestrando: Helio Sampaio Filho[4]

Orientadora: Anna Carolina Ratto Tempestini Horliana[1]

Pesquisadores participantes do projeto:

- Profa. Dra Kristianne Porta Santos Fernandes[1,2]
- Profa. Dra Raquel Agnelli Mesquita Ferrari[1,2]
- Profa. Dra Sandra Kalil Bussadori [1,2]
- Profa. Dra Daniela de Fátima Teixeira da Silva[1]
- Juliane Sotto Ramos [5]
- Danielly da Silva Lima[5]

[1] Professor, Programa de pós graduação em Biofotônica aplicada às Ciências da Saúde, Universidade Nove de Julho/UNINOVE

[2]Professor, Programa de pós graduação em Reabilitação, Universidade Nove de Julho/UNINOVE

[4]Aluno de Mestrado em Programa de pós graduação em Biofotônica aplicada às Ciências da Saúde, Universidade Nove de Julho/UNINOVE

[5]Aluna de iniciação científica Universidade Nove de Julho/UNINOVE

Endereço do Centro Coordenador: Universidade Nove de Julho (UNINOVE) Av. Vergueiro, 235/249 – Liberdade, São Paulo – SP

Os fenômenos relacionados a remoção dos terceiros molares inclusos tais como dor, edema e trismo acompanham praticamente todos os pacientes que necessitam este tipo de procedimento cirúrgico e este modelo tem sido amplamente utilizado para avaliar as opções terapêuticas que hoje se apresentam, como o uso de anti-inflamatórios, analgésicos associados ou não a aplicação da terapia com laser de baixa intensidade (Low Level Laser Therapy – LLLT). A necessidade de recuperação pós-operatória confortável e rápido retorno às atividades diárias aumentou a necessidade de controlar a inflamação pós-operatória, especialmente a dor e o edema. O uso de anti-inflamatório e analgésicos não estão livres de riscos e devem ser minimizadas sempre que possível. A associação entre o laser de baixa intensidade e a Acupuntura Auricular (Auriculoterapia) tem se

mostrado uma alternativa promissora, com baixo risco para efeitos colaterais, baixo custo e bem indicada para pacientes alérgicos ou com gastrite cronica. O objetivo deste estudo será avaliar a eficácia do laser de baixa intensidade nos pontos de auriluloterapia na redução da dor pós operatória nas exodontias de terceiros molares inferiores avaliar a eficiência da do uso da Auriculoterapia com o uso do Laser de baixa intensidade como meio auxiliar na diminuição da dor pós-operatória nas extrações de terceiros molares. Será usada uma amostragem de 40 pacientes, saudáveis, de ambos os gêneros com idade entre 18 e 30 anos com necessidade de remoção dos terceiros molares inferiores inclusos, bilaterais dentros da classificação de Pell & Gregory, triados e selecionados no ambulatório da Faculdade de Odontologia da Universidade Nove de Julho, UNINOVE, após aprovação pelo Comitê de Ética em Pesquisa desta entidade, que serão convidados após assinarem o termos de consentimento.

Introdução e Justificativa do Estudo

O pós-operatório em exodontias dos terceiros molares normalmente é acompanhado de dor e edema, e seu controle é essencial tanto para o paciente como para o cirurgião dentista. Normalmente ocorre reação inflamatória seguida de dor, edema e trismo assim diminuição da qualidade de vida dos sujeitos durante os três primeiros dias de pós-operatório (Pouchain *et al.*2015; Ferrante *et all* 2103; Markovic *et al* 2006)

Segundo Zuniga et al. (2004), cerca de 63% dos pacientes sentem dor intensa durante o primeiro dia pós-operatório, com maior intensidade 3 a 5 horas após o término da anestesia (Markovic, 2006).

Os antinflamatórios costumam ser prescritos de maneira preemptiva e também no pós-operatório, sendo os medicamentos mais indicados, os corticosteroides e os anti-inflamatórios não esteroidais, (Simone et al. 2013).

Entretanto alguns estudos demonstraram reações adversas aos anti-inflamatórios não esteroidais como distúrbios gastrointestinais (erosões, úlceras, dispepsia) com sérias complicações hemorrágicas, aumento de complicações cardiovasculares, insuficiência renal e alterações plaquetárias (Han 2014; Ramin , et al 2013, Wilcox 1997).

Entre as principais complicações locais das exodontias podemos citar a osteíte alveolar (alveolite seca), edema, trismo abcesso e dor, já entre as complicações sistêmicas febre, e alteração dos linfonodos. A grande maioria dos trabalhos (Poeschl et al 2004, Siddiqi, 2010, Kaczmarzyk T, 2009, Monaco, 2009)

que avalia a eficácia clínica dos antibióticos utilizam estes parâmetros de infecção como variáveis do estudo.

A necessidade de uma recuperação pós-operatória confortável e um rápido retorno às atividades diárias aumentou a importância de controlar a inflamação pós-operatória, especialmente no que diz respeito a dor e edema. A cirurgia para a remoção dos terceiros molares é considerada o padrão-ouro em estudos da dor (Zuniga *et al* -2004;)

Tornou-se muito comum por causa de sua alta sensibilidade nociceptiva local e o fato de que ele é normalmente indicado para pacientes jovens que normalmente não fazem uso de remédios regularmente. Na maioria dos casos, procedimentos muito semelhantes são realizados no mesmo paciente, uma em cada lado da mandíbula, de maneira que o indivíduo pode ter o seu próprio controle (boca dividida), a eliminação de possíveis diferenças individuais na resposta inflamatória e limiar da dor.

Atualmente o controle da dor e da inflamação é realizada com a associação de de um corticosteroide e um anti-inflamatórios não esteroidal (Han JB et al 2014) Hoje, uma nova alternativa tem surgido com resultados interessantes, o laser de baixa intensidade pode ser utilizado para minimizar esses efeitos. (Markovic AB, Todorovic L 2006). Os estudos tem demonstrado possibilidades promissoras para a modulação dos fenômenos relacionados à inflamação principalmente dor, edema e trismo: acupuntura auricular e laser de baixa intensidade .

Auriculopuntura ou Acupuntura Auricular

A Acupuntura, parte integrante de um sistema denominado Medicina Tradicional Chinesa (MTC) que consiste na aplicação de agulhas em determinados pontos distribuídos pela superfície do corpo. Vem sendo usada há muitos anos nas mais diversas áreas da Saúde, e cada vez mais, tem sido motivo de novos estudos. (...).

Uma das modalidades da acupuntura é a auriculoterapia que além do tradicional uso de agulhas possui, hoje em dia, outros tipos de estímulos, como é o caso da auriculo eletroacupuntura, laser auriculoterapia entre outros (Hising, C *et all,* 2104, Round, R *et all* 2013).A acupuntura auricular pode ser definida como sendo um sistema de diagnóstico e tratamento baseado na normalização das disfunções do organismo através da estimulação de pontos localizados na orelha (Gori, Firenzuol, 2007) , ou uma intervenção terapêutica na qual, estímulos na orelha externa são utilizados para aliviar condições de saúde em diversas partes do corpo (Oleson, 2003)

Existem duas escolas que desenvolveram o estudo e aplicação da auriculoterapia que segundo Round *et al* (2013) dividem-se em *acupuntura auricular antiga* e a *moderna acupuntura auricular*, sendo a primeira baseada na estimulação de pontos de acupuntura

na aurícula que estariam conectados aos meridianos e usados basicamente para a resolução da dor e a segunda na escola de Paul Nogier que pressupõem que uma organização somatotrópica do corpo é representada na orelha humana (Round, R *et al* 2013)

Outra divisão dos termos acima citados, segundo os mesmos autores, incluem o Sistema chinês de acupuntura auricular e o Sistema Europeu de acupuntura auricular além do desenvolvimento para auriculoterapia e auriculo medicina

Existem diversas proposições das bases teóricas da Auriculoterapia tais como a teoria neurológica, a embriológica, teoria dos microssistemas, a teoria energética da Medicina Tradicional Chinesa e das Bases Hormonais para tentar compreender os mecanismos de ação da mesma (Oleson, 2003).

Aplicação da laserterapia no pós operatório e as vantagens da sua utilização

Laser, acrônimo de Ligth Amplification by Stimulated Emission of Radiation) tem sido utilizado e estudado nos últimos 50 anos dentro das mais diversas áreas, tendo se tornado uma ferramenta essencial com aplicações às mais diversas, entre elas no monitoramento ambiental, metrologia de precisão, descontaminação biológica, transmissão e armazenamento de dados e nos tratamentos para a área de saúde.

Portanto a terapia a laser de baixa intensidade (Low Level Laser Therapy – **LLLT)** é a aplicação da radiação eletromagnética em um sistema biológico que promove a regeneração tecidual, redução da inflamação alivio da dor (Shirin F ,2014).

Diversos estudos têm mostrado a efetividade da aplicação do Laser de baixa intensidade na modulação e controle da dor pós operatória em cirurgia oral de modo geral e nos casos de exodontias de terceiros molares (He et al – 2014; Markovic et al 2006; Saber 2012).

O uso do Laser em pós-operatório de exodontias de terceiros molares impactados demonstram diminuição da dor, edema e trismo quando associados ao uso de anti-inflamatórios (Amarillas- Escobar 2010), embora outros estudos demonstrem somente uma melhora em relação à abertura bucal pós cirúrgica (Brignardello-Petersen *et all* – 2012) assim como somente no tocante a trismo e edema (Ferrante M *et all* – 2012) Até o presente momento, não existem estudos clínicos randomizados e controlados, que demonstrem a eficácia do uso a Auriculoterapia associada ao LASER de baixa intensidade para o controle da dor, edema e inflamação pós operatória

Objetivo geral

Avaliar a eficácia do laser de baixa intensidade nos pontos de auriluloterapia na redução da dor pós operatória nas exodontias de terceiros molares inferiores

Hipótese nula

O laser de baixa intensidade utilizado em pontos de auriculoterapia não é capaz de reduzir a dor pós operatória após cirurgias de terceiros molares inferiores.

Hipótese experimental

O laser de baixa intensidade utilizado em pontos de auriculoterapia é capaz de reduzir a dor pós operatória após cirurgias de terceiros molares inferiores.

Método

Após explicação verbal e por escrito do estudo, os pacientes que aceitarem participar assinarão o Termo de Consentimento Livre e Esclarecido (TCLE) após aprovação do Comitê de Ética em Pesquisa da Universidade Nove de Julho (UNINOVE). O estudo estará em conformidade com a Declaração de Helsinki (revisada em Fortaleza, 2013). A amostra será composta por pacientes 60 pacientes saudáveis de ambos os gêneros com idade entre 18 a 28 anos com necessidade de remoção cirúrgica dos terceiros molares inferiores desde que sejam bilaterais simétricos. Serão feitas duas cirurgias no mesmo paciente, ou seja é um estudo boca-divida (split-mouth) As cirurgias serão realizadas na Clínica Odontológica da Universidade Nove de Julho – UNINOVE da cidade de São Paulo, Brasil, no período de agosto de 2015 a agosto de 2016.

Critérios de exclusão

Serão excluídos os pacientes

- Alérgicos a qualquer fármaco utilizado na pesquisa (amoxicilina, paracetamol, clorexidina 2%),
- Gestantes ou em fase de amamentação,

- Fumantes,
- Submetidos à radioterapia na região da cabeça e pescoço,
- Infecção sistêmica ou local (ex: pericoronarite ou abscesso periodontal),
- Lesões ou imagens radiolúcidas, associadas aos dentes a serem extraídos,
- Que tenham utilizado anti-inflamatório ou antibiótico nos últimos 3 meses,
- Pacientes que durante a cirurgia apresentem qualquer tipo de complicação (ex: hemorragias, dificuldade operatória, tempo maior que 90 minutos de cirurgia), pois estes casos se distanciam do padrão esperado para as cirurgias de terceiros molares. Nesses casos será prescrito analgésico de ação central e os dados coletados para esse paciente não serão incluídos na pesquisa.
- Que utilizaram as medicações de forma diferente da maneira como foi prescrita

Critérios de inclusão

Serão incluídos no estudo pacientes:

- Com dentes na posição II B, segundo a classificação de Pell e Gregory (Figura 1 e Tabela 1).
- Com indicação para extração dos terceiros molares (infecções recorrentes, má posição, indicação ortodôntica) ou indicação profissional apresentada por escrito.
- ASA I (história médica negativa)
- Pressão arterial sistólica menor que 140mmHg e diastólica menor que 90mmHg e frequência cardíaca com valores 70 ± 20 batimentos /minuto.

Tabela 1. Classificação de Pell e Gregory (1933)

**Classificação de Pell e Gregory.**	
Classe I	Existe espaço suficiente entre o ramo e a distal do segundo molar para a acomodação do diâmetro mesio distal do terceiro molar
Classe II	O espaço entre o segundo molar e o ramo da mandíbula é menor que o diâmetro mesio distal do terceiro molar
Classe III	O terceiro molar está no ramo da mandíbula
A	A porção mais alta do terceiro molar está acima do plano oclusal.
B	A porção mais alta do terceiro molar está entre o plano oclusal e a linha cervical do segundo molar
C	A mais alta porção do terceiro molar está abaixo da linha cervical do segundo molar

Figura 1 - Radiografia panorâmica mostrando disposição anatômica semelhante bilateral e Classificação 2B.

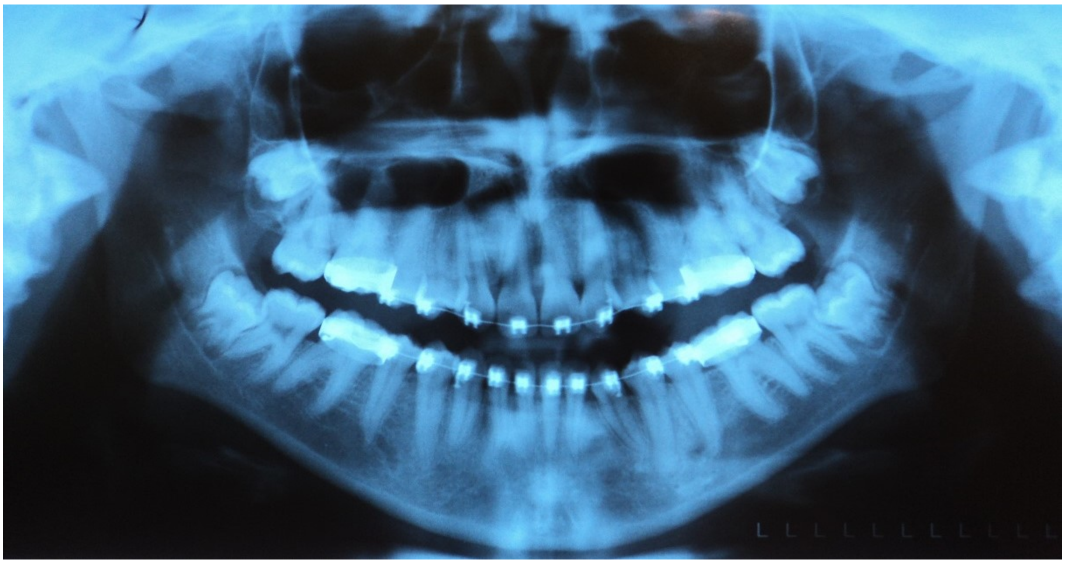

Calculo do tamanho da amostra

O tamanho da amostra será de 60 pacientes. Este valor foi estimado baseando-se em estudos preliminares e foi calculada para proporcionar força de 90% ($\alpha = 0,05$).

Treinamento e avaliação da calibração dos examinadores

Concordância intra-examinador

Um examinador (padrão ouro) fará o exercício de calibração com o objetivo de conseguir a máxima reprodutibilidade das medições realizadas. Serão avaliados 10 pacientes que não farão parte do estudo. Serão feitas avaliações para calibração das medidas de edema facial, abertura de boca e escala analógica visual. O coeficiente de correlação intraclasse (ICC) será calculado de forma a avaliar a concordância intra-examinador ≥ 0,90 em relação aos parâmetros clínicos citados (medidas continuas). Será utilizado paquímetro digital e régua milimetrada flexível. Os pacientes serão submetidos ao exame clinico periodontal por um examinador previamente calibrado, com o objetivo de realizar o diagnóstico. Essa avaliação será realizada no início do estudo.

Randomização, cegamento e composição dos grupos

Para distribuir aleatoriamente a ordem dos lados operados, será realizado um sorteio com 40 pares de números (1=Grupo experimental e 2= Grupo controle) através do programa Microsoft Excel, versão 2013. Conforme os números forem sorteados eles serão colocados no interior de envelopes opacos identificados com números sequenciais conforme a ordem obtida no sorteio. Os envelopes serão selados e permanecerão lacrados nessa mesma ordem numérica em um lugar seguro até o momento da realização das cirurgias. O sorteio e a preparação dos envelopes serão realizados por uma pessoa não envolvida no estudo.

Os pacientes que procurarem a Clínica Odontológica da Universidade Nove de Julho – UNINOVE da cidade de São Paulo no período de agosto de 2015 a agosto de 2016 que se encaixarem nos critérios de inclusão serão convidados a participar do estudo. Espera-se realizar triagem em aproximadamente 200 pacientes. Imediatamente antes da exodontia o cirurgião (H.S.) retirará e abrirá 1 envelope (sem alterar a sequência numérica dos demais envelopes) e realizará o procedimento indicado.

O cegamento será feito de forma que nem o paciente, e nem o cirurgião saberão qual o tratamento que estará sendo realizado (estudo duplo-cego). A única pessoa que saberá o tratamento realizado será o pesquisador responsável pela aplicação do LBI.

O desenho experimental consistirá em 2 grupos que receberão diferentes tratamentos para bocas divididas:

Grupo 1 (Experimental) (n=60 cirurgias) - Serão realizadas cirurgias de modo convencional e os pacientes receberão laser de baixa potência em pontos de auriculoterapia específicos para prevenção da dor e modulação da inflamação pós operatória, no período pós operatório imediato.

Grupo 2 (Controle) (n=60 cirurgias) – Serão realizadas cirurgias de modo convencional e os pacientes receberão laser de baixa potência desligados em pontos de auriculoterapia específicos para prevenção da dor e modulação da inflamação pós operatória, no período pos operatório imediato.

Para todos os pacientes será fornecido paracetamol 750mg para utilizarem em caso de dor. Será fornecida uma receita de Paracetamol com Fosfato de Codeína comprimido 30 mg Tylex® Janssen-Cilag caso o paciente sinta uma dor muito forte. Os dados desses pacientes não serão incluídos no estudo, sem prejuízo do tratamento do paciente.

Anamnese

Na anamnese, além das perguntas tradicionais relacionadas à saúde geral do paciente, serão coletados dados demográficos (idade, sexo, estado civil, ocupação, nível educacional, condições de vida, salário), dados da história médica (queixa principal, estado da doença atual, história médica e medicamentos) hábitos e vícios (tabagismo, etilismo)

Análise do perfil de impacto de saúde oral (Questionário OHIP-14)

O Perfil de Impacto de Saúde Oral (OHIP-14) é uma forma simplificada do questionário original OHIP, utilizado para a avaliação do impacto da saúde bucal na qualidade de vida dos sujeitos da pesquisa (De Oliveira; Nadanovsky, 2005). Esse questionário está indicado para avaliação da qualidade de vida durante o período pós operatório de pacientes submetidos a exodontias de terceiros molares inferiores (Negreiros, 2012). Os itens são distribuídos entre as seguintes sub-escalas: limitação

funcional, dor, desconforto psicológico, deficiência física, deficiência psicológica, incapacidade social e deficiência.
Essa avaliação será realizada no dia da anamnese (baseline), 1,2, 3 e 7 dias sempre pelo mesmo avaliador calibrado no início do estudo para o preenchimento do questionário

Verificação dos sinais vitais

Os sinais vitais como frequência cardíaca, pressão arterial, e temperatura corpórea, serão medidos em todas as visitas do estudo. Pressão arterial será medida usando-se Omron-75 (HEM-759-E [EU]) sob posição sentada após 10 minutos de repouso. Para o cálculo do índice de massa corpórea (IMC) serão avaliados altura e peso durante a visita de triagem.

Técnica cirúrgica

Todos os pacientes serão operados com a mesma técnica cirúrgica, uma vez que o grau de dificuldade será padronizado pela classificação de Pell e Gregory, 1933 e sempre pelo mesmo operador especialista em cirurgia buco maxilo-facial (H.S.). Haverá 21 dias de período de whashout entre os lados operados.
Todos os pacientes serão submetidos às seguintes manobras cirúrgicas:

- - Antissepsia extra-oral com Clorexidina 2%
- - Antissepsia intra-oral com Periogard®
- - Colocação de campos cirúrgicos estéreis
- - Anestesia do nervo alveolar inferior
- - Anestesia infiltrativa terminal do nervo bucal
- - Incisão para realização de retalho muco periostal se estendendo do ramo mandibular até a distal do primeiro molar onde será realizada uma incisão relaxante
- - Descolamento do retalho
- - Osteotomia na tábua óssea vestibular e distal do Terceiro Molar inferior com espessura igual a da broca e profundidade suficiente para alcançar a medular óssea
- - Odontossecção da porção medial da coroa

- - Luxação e avulsão do dentes com elevadores
- - Irrigação com Soro Fisiológico
- - Sutura

Metodologia para aplicação dos pontos de Auriculopuntura –

O operador fará inicialmente a marcação de pontos a serem utilizados, na auricula externa do paciente a ser operado com caneta tipo gel vermelha.

Serão escolhidos os seguintes pontos para aplicação de LLTT na auricula escolhida, baseados em OLESON, T (1996) :

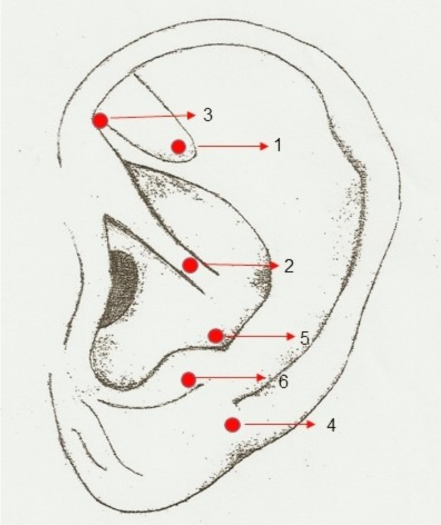

1) Shen Men
2) Zero Point
3) Autonomic (Sympathetic)
4) Lower jaw
5) Tootache 3
6) Sub córtex

Justificativa para a escolha dos pontos de Auriculo puntura

A World Federation of Acupuncture-Moxabustion Societies (WFAS), devido ao fato de haver inúmeras nomenclaturas e mapas de Auriculoterapia utilizados em diversos países, estabeleceu algumas normas para a estandardização dos pontos de acupuntura auricular (Wang L et al 2013) reunindo diversos especialistas que promoveram a denominada ISAAPs (International standart of auricular acupunture points). A nomenclatura e localização publicada foi baseada na subdivisão e modelos propostos por Oleson (OLESON,T – 1996).

Baseado nestes dados, os pontos a serem utilizados neste estudo serão os acima listados cujas funções são:

1) Shen Men -

Função principal: aliviar stress, dor, tensão, ansiedade, doenças inflamatórias.

2) Zero Point

Função principal – auxilia no equilíbrio da homeostasia, serve de suporte para a manutenção dos outros pontos

3) Autonomic (Chinese Sympathetic point)

Função principal – redução do desequilíbrio neuro-vegetativo

4) Lower jaw –

Função principal – Alivio da dor dos dentes inferiores

5) Tootache 3

Função principal – Alivio de dor de dentes em geral

6) Sub córtex

Função principal – Redução de dores cronicas

Após a marcação dos pontos padronizados, será realizada a aplicacação do Laser pelo auxiliar que obedecerá a escolha aleatória (se o paciente pertence ao Grupo 1 ou 2) já descrita, sem que haja contato do cirurgião nesta escolha.

Ao término na Cirurgia será realizada a avaliação prevista de pós operatório imediato (T_{PO1}).

Especificações do Laser de Baixa intensidade

Será utilizado o laser diodo vermelho (Therapy XT®), comprimento de onda de 660 nm (±10nm).

Medicações

- - Amoxilicilina cápsula 500mg Novocilin® Ache
- - Gluconato de Clorhexidina 0,12% colutório Periogard® Colgate
- - Paracetamol comprimido 750 mg Paracetamol® Janssen-Cilag
- - Mepivacaina 2% com adrenalina 1:100.000 Mepiadre® DFL

Avaliações pós-tratamento

Todos os pacientes receberão avaliações de dor pós operatória, edema, abertura bucal, linfadenopatia, disfagia, quantidade de analgésicos ingeridos, febre, presença local de infecção e qualidade de vida. Essas variáveis do estudo serão avaliadas no baseline (antes de iniciar o estudo), após 24 horas, após 36 horas e após 48 horas e então após 1 semana quando o paciente vier retirar a sutura. A segunda avaliação será feita da mesma maneira e na mesma ordem quando for realizada a segunda cirurgia.

Também serão realizadas coletas de sangue de todos os pacientes para avaliação das citocinas inflamatórias no soro no baseline e após 24 horas após a realização da cirurgia (segundo o item "Níveis plasmáticos de marcadores inflamatórios").

Variáveis clínicas

Para a avaliação da dor pós operatória serão analisados os sinais clínicos (variáveis do estudo) nos tempos imediatamente após o término da cirurgia (T_{PO1}), após 48 horas (T_{PO2}), no sétimo dia após a cirurgia no qual será feita a remoção da sutura(T_{PO7}). A segunda avaliação será feita da mesma maneira e na mesma ordem quando for realizada a segunda cirurgia. Serão analisados os principais sinais e sintomas que caracterizam o quadro infeccioso: presença de trismo, edema, dor pós-operatória, disfagia, febre, palpação de linfonodos e avaliação de qualquer quadro infeccioso.

Para a avaliação da presença de trismo será utilizada a medida inter-incisal com um paquímetro digital. Durante o exame clinico (realizado uma semana antes do experimento) será solicitado ao paciente que execute sua abertura bucal máxima, quando será medida em milímetros, a distância entre a borda incisal do incisivo central superior e inferior (Siddiqi, 2010). Serão admitidos somente os pacientes que possuírem os incisivos centrais superiores e inferiores. Essa medida será obtida no pré-operatório, considerada valor basal (T_{basal}) e nos períodos pós-operatórios já descritos

Para avaliar o edema, será utilizado o método de mensuração descrito por Neupert; Lee; Philput. (1992). As mensurações serão feitas através de distâncias lineares entre o ângulo da mandíbula e os seguintes pontos: ponta inferior do trágus, porção mais inferior da asa do nariz, canto externo do olho, comissura labial e mento (na região da linha média). A mensuração será feita com auxílio de uma régua flexível e as marcações

dos pontos serão realizadas com uma caneta demográfica, ficando permanente o ângulo da mandíbula desde o pré-operatório (valor basal) para que as medidas possam ser padronizadas.

A dor será avaliada através de aplicação de uma escala visual analógica (VAS), esta consiste em uma linha de 100 mm, numerada a cada centímetro, e com os dois extremos fechados. Um dos extremos apresenta a indicação "0" e no outro "100" que significa respectivamente sem dor e dor insuportável. As instruções sobre o preenchimento do prontuário de avaliação de dor serão dadas pelo pesquisador ao paciente, e serão dadas as explicações que auxiliarão o paciente no preenchimento. Cada paciente será orientado a marcar com um traço vertical o ponto que melhor corresponder a intensidade de dor no momento da avaliação (Mark, 1986). A dor será avaliada nos mesmos dias de pós operatório dos outros parâmetros avaliados por um mesmo profissional da área odontológica (dias marcados para o retorno) e pelo próprio paciente nos seguintes horários: 6 horas após a cirurgia, à noite (entre 22:00-23:00 hs) e pelos seguintes 6 dias - de manhã (entre 7:00-9:00 hs) e à noite (entre 22:00-23:00 hs)

I---I

0 100mm

A avaliação da disfagia será realizada através de uma escala numérica em que 0 pontos indicará ausência total de disfagia; 1 ponto, disfagia a alimentos sólidos somente e 2 pontos, disfagia a qualquer alimento líquido ou sólido.

Avaliação de marcadores inflamatórios

Coleta de plasma

As coletas de sangue serão obtidas por punção venosa, realizadas por um técnico treinado no baseline e 48 horas após as exodontias. As amostras serão armazenadas a -80ºC até sua utilização. Será realizada contagem de leucócitos e determinação de níveis plasmáticos dos marcadores inflamatórios fator de TNF-α, PCR, IL1-α e IL1-β, IL-6 e IL-8 por ELISA, usando kits comerciais (Peprotech Inc., Rocky Hill, NJ, EUA) conforme instruções do fabricante. Amostras com sangue serão desprezadas e o material será armazenado em tubo seco a -80ºC até a sua utilização.

Referências Bibliográficas

Adel Al-Asfour Postoperative Infection after Surgical Removal of Impacted Mandibular Third Molars:An Analysis of 110 Consecutive Procedures Med Princ Pract 2009;18:48–52

Amarillas-Escobar E D - Use of Therapeutic Laser After Surgical Removal of Impacted Lower Third Molars - American Association of Oral and Maxillofacial Surgeons J Oral Maxillofac Surg 68:319-324, 2010

Ataoglu H; Oz GY; Candirli C; Kiziloglu D.Routine antibiotic prophylaxis is not necessary during operations to remove third molars.British Journal of Oral and Maxillofacial Surg. 2008;46: 133–5.

Babatunde, O B et al Prospective, Randomized, Open-Label, Pilot
Bergdahl L. Hedstr¨om 2004 Metronidazole for the prevention of dry socket after removal of partially impacted mandibular third molar: a randomised controlled trial British Journal of Oral and Maxillofacial Surgery (2004) 42, 555—558

Cho S, Ki Y, Chu V, Chan J. Impaction of permanent Mandibular Second Molars in Ethnic Chinese schoolchildrenJCDA • www.cda-adc.ca/jcda • July/August 2008, Vol. 74, No. 6

Clauser B, Barone R, Briccoli L, Baleani A. Complications in surgical removal of mandibular third molars.Minerva Stomatol. 2009 Jul-Aug;58(7-8):359-66.

Clauser B, Barone R, Briccoli L, Baleani A.Complications in surgical removal of mandibular third molars. Minerva Stomatol. 2009 Jul-Aug;58(7-8):359-66.

Clinical Trial Comparing the Effects of Dexamethasone Coadministered with Diclofenac Potassium or Acetaminophen and Diclofenac Potassium Monotherapy After Third-Molar Extraction in Adults, currente Therapeutic Research, 2006 Jul/Aug 67(4)

Doeuk,C et all - Current indications for low level laser treatment inmaxillofacial surgery: a review- - British Journal of Oral and Maxillofacial Surgery 53 (2015) 309–315

Epstein JB, Chong S, Le ND. A survey of antibiotic use in dentistry. Journal of the American Dental Association 2000;131:1600-9.

Ferrante, M - Effect of low-level laser therapy after extraction of impacted lower third molars - Lasers Med Sci (2013) 28:845–849

Haas DA, Epstein JB, Eggert FM. Antimicrobial resistance: dentistry's role. Journal of the Canadian Dental Association 1998;64:496-502.

Han JB et a Postoperative gastrointestinal bleeding in orthognathic surgery patients: its estimated prevalence and possible association to known risk factors.J Oral Maxillofac Surg, 2014 Oct;72(10):2043-51. doi: 10.1016/j.joms.2014.02.039. Epub 2014 Mar 13.

He WL , Yu FY, Pan J, Zhuang R, Duam PJ - A systematic review and meta-analysis on the efficacy of low-level laser therapy in the management of complication after mandibular third molar surgery. 2014- Lasers Med Sci

Hersh EV. Adverse drug interactions in dental practice: interactions involving antibiotics. Journal of the American Dental Association 1999;130:236-51.

Huovinen P, Cars O Control of antimicrobial resistance: time for action BMJ 1998; 317 : 6131998

Jaafar N, Nor GM. The prevalence of post-extraction complications in an outpatient dental clinic in Kuala Lumpur Malaysia--a retrospective survey. Singapore Dent J. 2000 Feb;23(1):24-8.

Jensen M.P, Karoly P, Braver S. The measurement of clinical pain intensity: a comparison of six methods. Pain. 1986;27:117–126.

Kaczmarzyk T, Wichlinski J, Stypulkowska J , Zaleska M, Panas M, Woron J.Single-dose and multi-dose clindamycin therapy fails to demonstrate efficacy in preventing infectious and inflammatory complications in third molar surgery. Int. J.Oral Maxillofac. Surg. 2007; 36: 417–422.

Kim T W, Årtun J, Behbehani F, Artese F Prevalence of third molar impaction in orthodontic patients treated nonextraction and with extraction of 4 premolars American Journal of Orthodontics and Dentofacial Orthopedics Volume 123, Number 2

Lawler B, Sambrook PJ, Goss AN. Antibiotic prophylaxis for dentoalveolar surgery: is it indicated? Aust Dent J. 2005 Dec;50(4 Suppl 2):S54-9.

Lodi G, Sardella A, Bez C, Demarosi F, Carrassi A. Antibiotics to prevent complications following tooth extractions (Protocol for a Cochrane Review). In: The Cochrane Library, Issue 1, 2010.

Louise C. Sweeney1, Jayshree Dave1,2, Philip A. Chambers3 and John Heritage1* Antibiotic resistance in general dental practice—a cause for concern? Journal of Antimicrobial Chemotherapy (2004) 53, 567–576

Malamed SF. Handbook of local anesthesia. 5nd ed. St Louis: Mosby-Year Book; 2005

Mark P. Jensena, Paul Karoly, a and Sanford Bravera The measurement of clinical pain intensity: a comparison of six methods Pain Volume 27, Issue 1, October 1986, Pages 117-126

Markovic AB, Todorovic L , Postoperative analgesia after lower third molar surgery: contribution of the use of long-acting local anesthetics, low-power laser, and diclofenac. Oral Surg, Oral Med Oral Pathol Oral Radio Endod 2006 Nov;102(5):e4-8. Epub 2006 Aug 10.

Mehrabi M, Allen JM, Roser SM.Therapeutic agents in perioperative third molar surgical procedures. Oral Maxillofac Surg Clin North Am. 2007 Feb;19(1):69-84,

Monaco G, Tavernese L, Agostini R, Marchetti C. Evaluation of antibiotic prophylaxis in reducing postoperative infection after mandibular third molar extraction in young patients. J Oral Maxillofac Surg. 2009 Jul;67(7):1467-72.

Neupert EA, Lee JW, Philput CB. Evatuation of dexamethasone for reduction of postsurgical sequelae of third molar removal. J Oral Maxillofac Surg. 1992; 50:1177-83

Nogueira AS, Ponzoni D, Pasinato E, Ferrari LK, FariasRD. Principais Transtornos Ocasionados por Dentes Inclusos. Rev APCD. 1997; 51: 247-9.

Paschoal M A B et all - Therapeutic Effects of Low-Level Laser Therapy After Premolar Extraction in Adolescents: A Randomized Double-Blind Clinical Trial - Photomedicine and Laser Surgery Volume 30, Number 9, 2012

Paulo RSMF, Marta RP, Thiago SS, Silva LCF, Lélia BS Evaluation of prevalence of pathologic conditions in impacted wisdom Teeth Rev. Cir. Traumatol. Buco-Maxilo-fac., Camaragibe v.8, n.3, p. 41 - 48, jul./set. 2008

Pell GJ, Gregory BT. Impacted mandibular third molars: Classification and modified techniques for removal. Dent Digest 1933; 39:330-8.

Peterson LJ: Antibiotic prophylaxis against wound infections in oral and maxillofacial surgery. J Oral Maxillofac Surg. 1990; 48:617.

Peterson LJ: Princípios do tratamento de dentes impactados In: Peterson LJ; Ellis E; Hupp JR; Tucker M.R: Cirurgia oral e maxilofacial contemporânea. 4ª Ed. Rio de Janeiro: Mosby; 2005. p.197-226.

Peterson, L.J.; Ellis, E.; Hupp, J.R.; Tucker, M.R. - Cirurgia Oral e Maxilofacial

Poeschl PW, Eckel D, Poeschl E. Postoperative prophylactic antibiotic treatment in third molar surgery — a necessity? J Oral Maxillofac Surg 2004; 62:3–8.

Pouchain E.C., Comparative efficacy of nimesulide and ketoprofen on inflammatory events in third molar surgery: a Split-mouth, prospective, randomized, double-bilnd study. Int. J Oral maxillofac surg 2015 apr

Quek SL, Tay CK, Tay KH, Toh SL, Lim KC. Pattern of third molar impaction in a Singapore Chinese population: a retrospective radiographic survey. Int J Oral Maxillofac Surg. 2003 Oct;32(5):548-52.

Rabischong P, Terral C, MD Scientific Basis of Auriculotherapy: State of the Art – Medical Acupuncture Volume 26, Number 2, 2014

Ramin M, et al - Lower gastrointestinal adverse effects of NSAIDS: an extreme example of a common problem - BMJ Case Rep 2013. doi:10.1136/bcr-2012-008274

Saber K , Chiniforush L, Shahabi S - The effect of low level laser therapy on pain reduction after third molar surgery.; Minerva Stomatol 2012 Jul-Aug;61(7-8):319-22.

Sekhar C, Narayanan V, Baig M. Role of antimicrobials in third molar surgery: prospective, double blind, randomized, placebo-controlled clinical study. British Journal of Oral and Maxillofacial Surgery 2001;31:134–7

Shirin F et al - Biological Effects of Low Level Laser Therapy – 2014 – J Lasers Med Sci 5 (2) 58-62

SiddiqiA, Morkel JA, Zafar Z. Antibiotic prophylaxis in third molar surgery: A randomized double-blind placebo-controlled clinical trial using split-mouth techniqueA Int. J. Oral Maxillofac. Surg. 2010; 39: 107–114 .

Silveira HM, Ramos JR JWN, Pereira RA. Profilaxia antibiótica para remoção de terceiros molares. Rev Bras Odontolol . 2003;60(3):188-191.

Sweeney LC, Dave J, Chambers PA, Heritage J Antibiotic resistance in general dental practice—a cause for concern? Journal of Antimicrobial Chemotherapy (2004) 53, 567-576

Thomas DW, Hill CM. An audit of antibiotic prescribing in third molar surgery.Br J Oral Maxillofac Surg 1997; 35:126–128.

Wang L et all - Status and strategies analysis on international standardization of auricular acupuncture points- Tradit Chin Med 2013 June15;33(3):408-412

Wilcox CM, Alexander LN, Cotsonis GA, et al. Nonsteroidal anti-inflammatory drugs are associated with both upper and lower gastrointestinal bleeding. Dig Dis Sci 1997;42:990–7.

Zuniga Jr et all, Analgesic safety and efficacy of diclofenac sodium softgels onpostoperative third molar extraction pain – J Oral Maxillofac Surg 2004; Jul;62(7):806-15

Yamalik K, Bozkaya S. The predictivity of mandibular position as a risk indicatior for pericoronitis. Clin Oral Inv. 2007; in press.
