# Supplementary material for: Low-level laser treatment applied at auriculotherapy points to reduce postoperative pain in third molar surgery: A randomized, controlled, single-blinded study
Source: PLoS One. 2018 Jun 19;13(6):e0197989. doi: 10.1371/journal.pone.0197989 (PMC6007895; doi:10.1371/journal.pone.0197989)
Supplement: S7 File — (PDF) [file pone.0197989.s007.pdf]

Mixed effects models: RESULTS

Primary outcome: Pain – random effects at individual level

```
Performing EM optimization:

Performing gradient-based optimization:

Iteration 0:   log likelihood = -669.19313
Iteration 1:   log likelihood = -668.65463
Iteration 2:   log likelihood = -668.61853
Iteration 3:   log likelihood = -668.60388
Iteration 4:   log likelihood = -668.60276
Iteration 5:   log likelihood =  -668.6025
Iteration 6:   log likelihood = -668.60248
Iteration 7:   log likelihood = -668.60247

Computing standard errors:

Mixed-effects ML regression                     Number of obs     =        279
Group variable: ID                              Number of groups  =         70

                                                Obs per group: min =         3
                                                               avg =       4.0
                                                               max =         4


                                                Wald chi2(4)      =       6.29
Log likelihood = -668.60247                     Prob > chi2       =     0.1782

------------------------------------------------------------------------------
        pain |      Coef.   Std. Err.      z    P>|z|     [95% Conf. Interval]
-------------+----------------------------------------------------------------
       Group |  -.1647912   .3700948    -0.45   0.656    -.8901638    .5605813
timesurgery |   .0095654   .0134757     0.71   0.478    -.0168465    .0359773
         Sex |   .4230251   .3939264     1.07   0.283    -.3490565    1.195107
         med |   .1338811   .0688271     1.95   0.052    -.0010176    .2687798
       _cons |   1.438481   .7404546     1.94   0.052     -.012783    2.889746
------------------------------------------------------------------------------

------------------------------------------------------------------------------
  Random-effects Parameters  |   Estimate   Std. Err.     [95% Conf. Interval]
-----------------------------+------------------------------------------------
ID: Independent              |
                  sd(Group)  |   .0007594   .0018023      7.25e-06    .0795342
                  sd(_cons)  |   .8673374   .2470927      .4962405    1.515947
-----------------------------+------------------------------------------------
               sd(Residual)  |   2.532962   .1238486      2.301492    2.787713
------------------------------------------------------------------------------
LR test vs. linear regression:       chi2(2) =      4.11   Prob > chi2 = 0.1282

Note: LR test is conservative and provided only for reference.
```

Plot: fitted against residuals

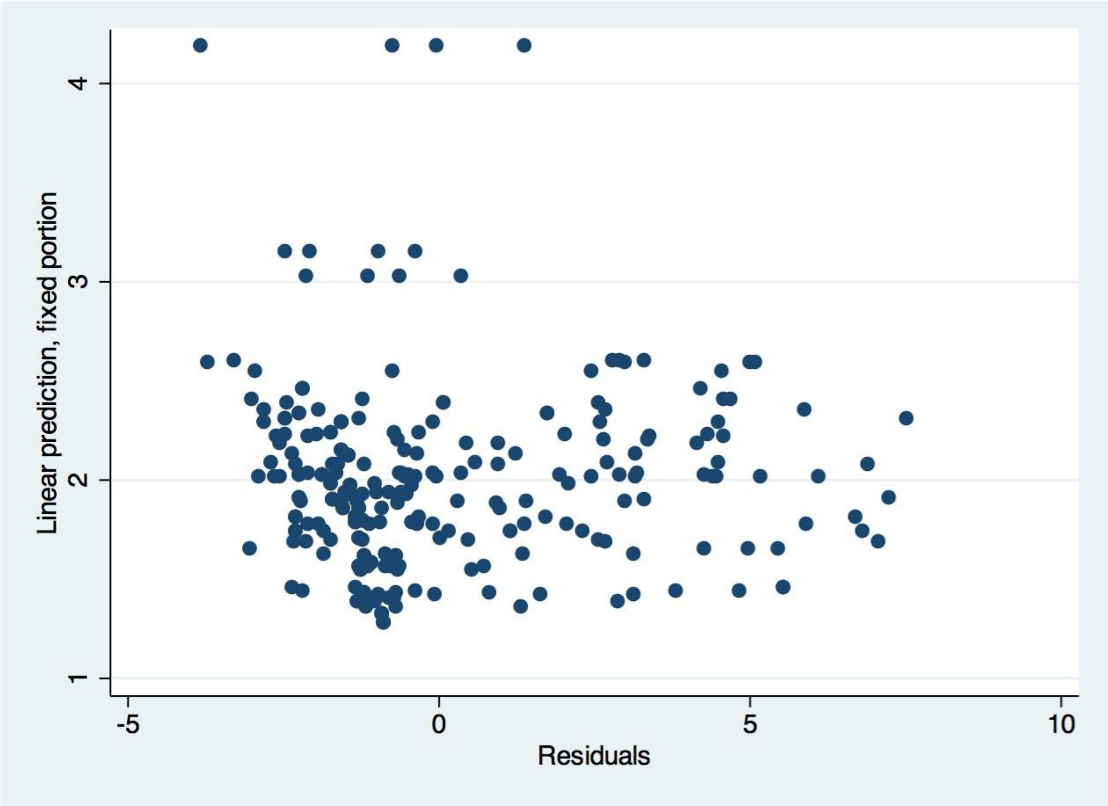

Secondary Outcomes:

Edema I

```
Performing EM optimization:

Performing gradient-based optimization:

Iteration 0:   log likelihood = -131.59785
Iteration 1:   log likelihood = -131.09984
Iteration 2:   log likelihood = -131.03564
Iteration 3:   log likelihood = -131.03099
Iteration 4:   log likelihood = -131.03087
Iteration 5:   log likelihood = -131.03087

Computing standard errors:

Mixed-effects ML regression                     Number of obs      =       274
Group variable: ID                              Number of groups   =        69

                                                Obs per group: min =         3
                                                               avg =       4.0
                                                               max =         4


                                                Wald chi2(4)       =     15.20
Log likelihood = -131.03087                     Prob > chi2        =    0.0043
```

EI	Coef.	Std. Err.	z	P>|z|	[95% Conf.	Interval]
Group	.0214975	.1583823	0.14	0.892	-.2889261	.3319212
timesurgery	.0117535	.0057251	2.05	0.040	.0005325	.0229745
Sex	-.5212109	.1697558	-3.07	0.002	-.8539261	-.1884956
med	.0491453	.0292664	1.68	0.093	-.0082157	.1065064
_cons	11.02672	.3202591	34.43	0.000	10.39902	11.65441

Random-effects Parameters	Estimate	Std. Err.	[95% Conf.	Interval]
ID: Independent				
sd(Group)	1.14e-09	3.36e-09	3.45e-12	3.74e-07
sd(_cons)	.6390626	.0566994	.5370596	.7604389
sd(Residual)	.2604199	.0128615	.2363934	.2868882

```
LR test vs. linear regression:       chi2(2) =    311.97   Prob > chi2 = 0.0000

Note: LR test is conservative and provided only for reference.
```

Plot: fitted against residuals

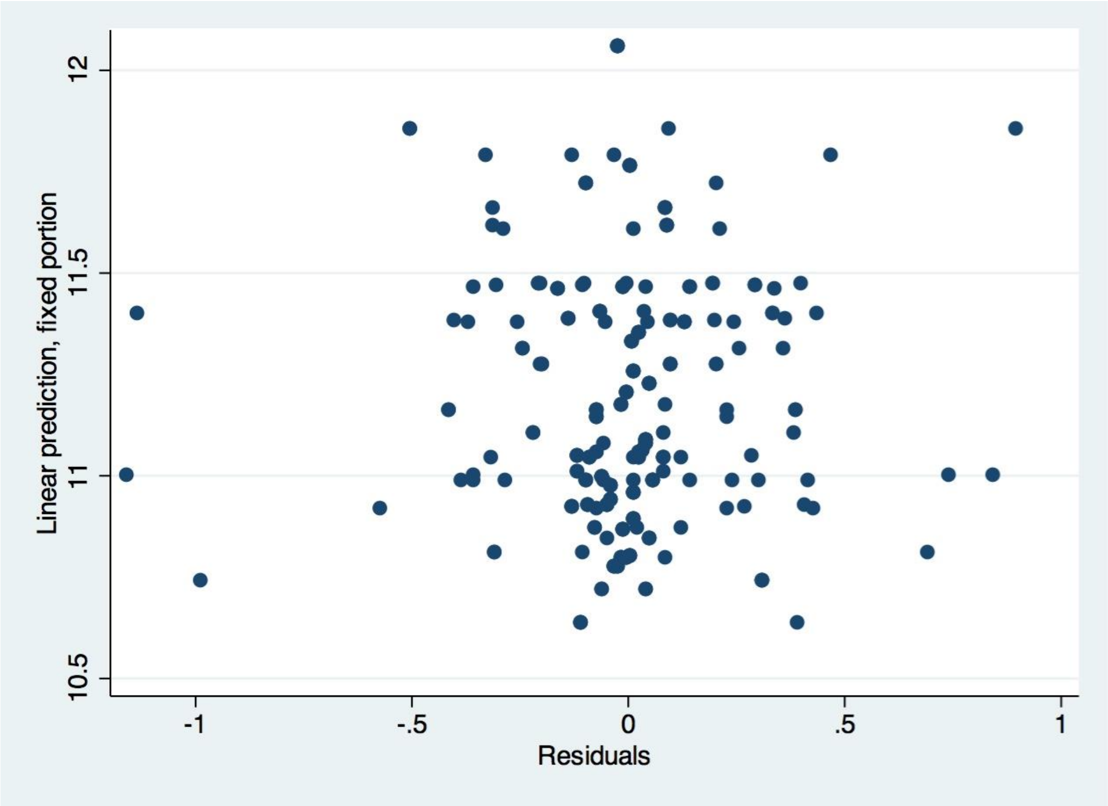

Edema II

```
Performing EM optimization:

Performing gradient-based optimization:

Iteration 0:   log likelihood = -203.21934
Iteration 1:   log likelihood = -203.21933

Computing standard errors:

Mixed-effects ML regression                     Number of obs     =        274
Group variable: ID                              Number of groups  =         69

                                                Obs per group: min =         3
                                                               avg =       4.0
                                                               max =         4


                                                Wald chi2(4)      =      13.55
Log likelihood = -203.21933                     Prob > chi2       =     0.0089
```

EII	Coef.	Std. Err.	z	P>|z|	[95% Conf.	Interval]
Group	.0062347	.1375756	0.05	0.964	-.2634085	.2758779
timesurgery	.0106073	.0049035	2.16	0.031	.0009966	.0202181
Sex	-.4338876	.1450163	-2.99	0.003	-.7181144	-.1496608
med	.0282751	.0242454	1.17	0.244	-.0192451	.0757953
_cons	11.37722	.2598666	43.78	0.000	10.86789	11.88655

Random-effects Parameters	Estimate	Std. Err.	[95% Conf.	Interval]
**ID:** Independent				
sd(Group)	.2428628	.0802972	.127037	.4642927
sd(_cons)	.3731662	.1160578	.2028483	.6864883
sd(Residual)	.3892602	.0192168	.3533607	.4288069

```
LR test vs. linear regression:       chi2(2) =    148.10   Prob > chi2 = 0.0000

Note: LR test is conservative and provided only for reference.
```

Plot: fitted against residuals

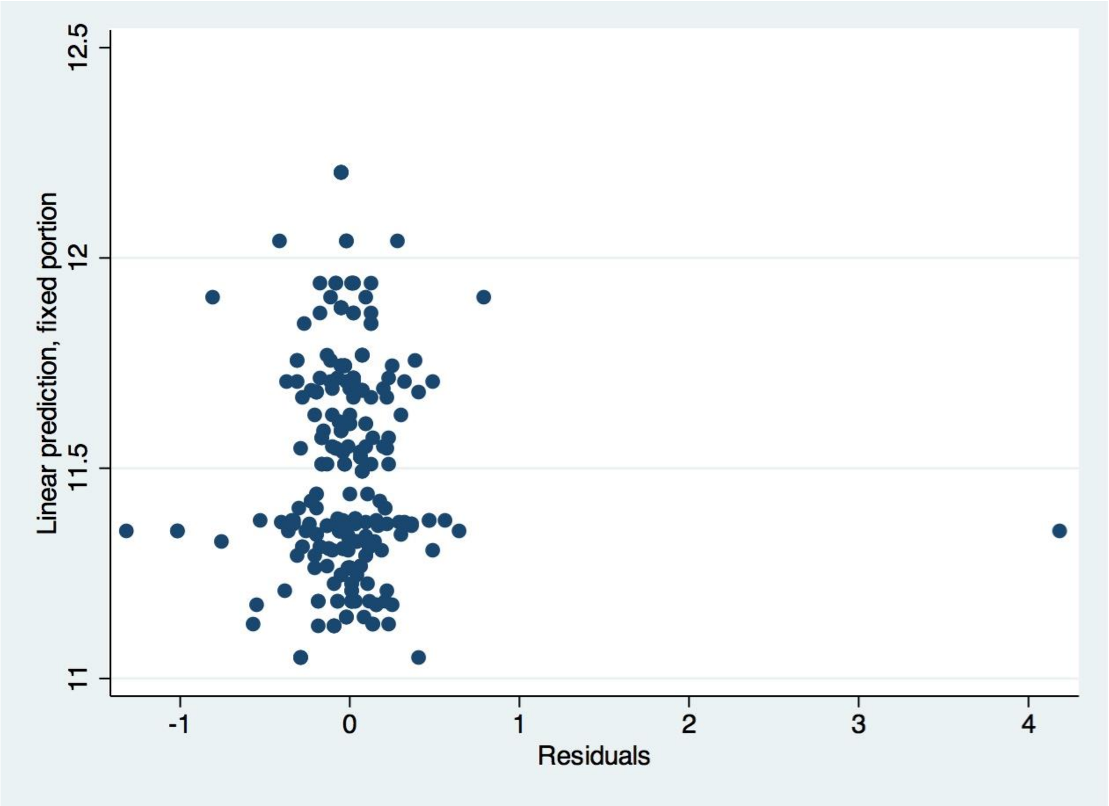

Edema III:

Performing EM optimization:

Performing gradient-based optimization:

Iteration 0: log likelihood = **-347.91976**
Iteration 1: log likelihood = **-347.91475**
Iteration 2: log likelihood = **-347.91475**

Computing standard errors:

Mixed-effects ML regression — Number of obs = **274**
Group variable: **ID** — Number of groups = **69**

Obs per group: min = **3**
avg = **4.0**
max = **4**

Wald chi2(**4**) = **19.09**
Log likelihood = **-347.91475** — Prob > chi2 = **0.0008**

EIII	Coef.	Std. Err.	z	P>|z|	[95% Conf.	Interval]
Group	-.0911212	.2349157	-0.39	0.698	-.5515476	.3693052
timesurgery	.0133311	.0083127	1.60	0.109	-.0029614	.0296237
Sex	-1.009465	.2457358	-4.11	0.000	-1.491098	-.5278316
med	.0521301	.040936	1.27	0.203	-.028103	.1323632
_cons	15.68423	.4387743	35.75	0.000	14.82425	16.54421

Random-effects Parameters	Estimate	Std. Err.	[95% Conf.	Interval]
**ID:** Independent				
sd(Group)	.4397295	.132083	.244066	.7922529
sd(_cons)	.5965783	.2072694	.3019494	1.178693
sd(Residual)	.6593231	.0325512	.5985136	.726311

LR test vs. linear regression: chi2(**2**) = **150.90** Prob > chi2 = **0.0000**

Note: LR test is conservative and provided only for reference.

Plot: fitted against residuals

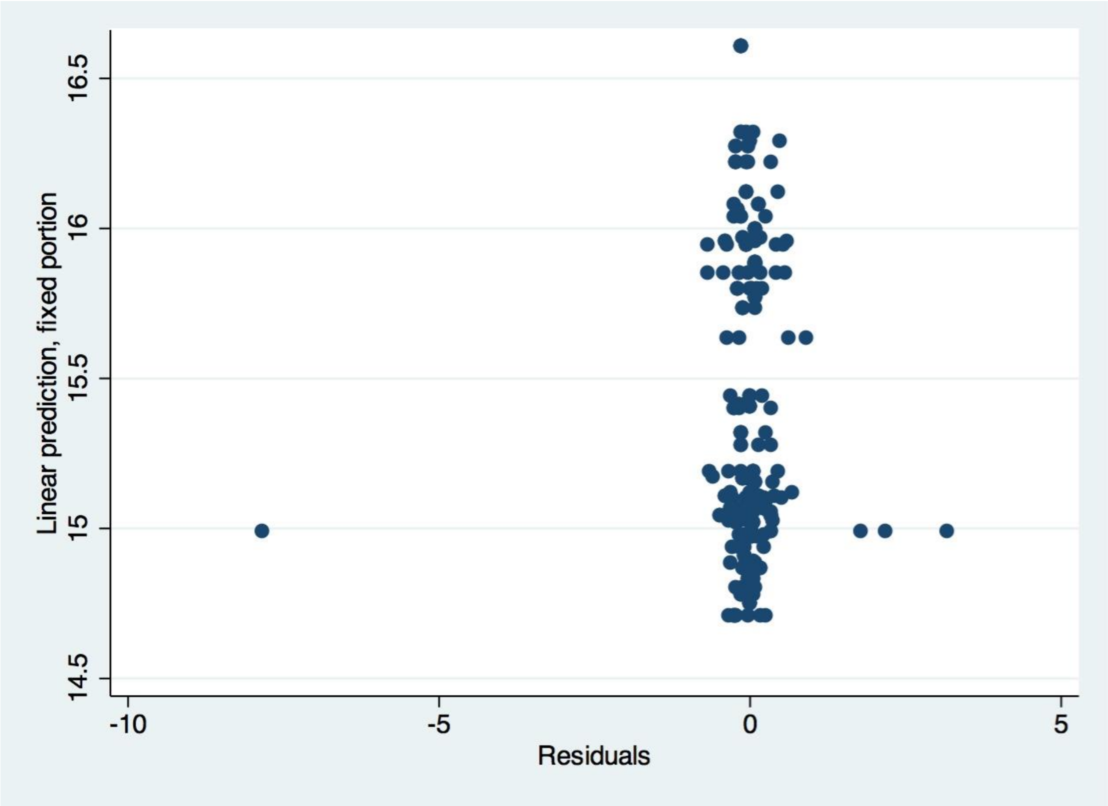

Mouth opening

```
Performing EM optimization:

Performing gradient-based optimization:

Iteration 0:   log likelihood = -1058.7689
Iteration 1:   log likelihood = -1057.7628
Iteration 2:   log likelihood = -1057.6697
Iteration 3:   log likelihood =  -1057.669
Iteration 4:   log likelihood =  -1057.669

Computing standard errors:

Mixed-effects ML regression                     Number of obs     =        275
Group variable: ID                              Number of groups  =         69

                                                Obs per group: min =         3
                                                               avg =       4.0
                                                               max =         4


                                                Wald chi2(4)      =      16.79
Log likelihood =  -1057.669                     Prob > chi2       =     0.0021

------------------------------------------------------------------------------
   mouthopen |      Coef.   Std. Err.      z    P>|z|     [95% Conf. Interval]
-------------+----------------------------------------------------------------
       Group |  -1.141423   1.948796    -0.59   0.558    -4.960993    2.678148
 timesurgery |   .0093853   .0704088     0.13   0.894    -.1286135     .147384
         Sex |  -7.339091   2.087973    -3.51   0.000    -11.43144    -3.24674
         med |   -.632364   .3599867    -1.76   0.079    -1.337925     .073197
       _cons |   45.59093   3.939712    11.57   0.000     37.86924    53.31262
------------------------------------------------------------------------------

------------------------------------------------------------------------------
  Random-effects Parameters  |   Estimate   Std. Err.     [95% Conf. Interval]
-----------------------------+------------------------------------------------
ID: Independent              |
                  sd(Group)  |   3.01e-07   9.54e-07      6.08e-10    .0001491
                  sd(_cons)  |   6.237473   .9005327      4.700205    8.277526
-----------------------------+------------------------------------------------
               sd(Residual)  |    10.0841   .4965672      9.156334    11.10586
------------------------------------------------------------------------------
LR test vs. linear regression:       chi2(2) =    25.34   Prob > chi2 = 0.0000

Note: LR test is conservative and provided only for reference.
```

Plot: fitted against residuals

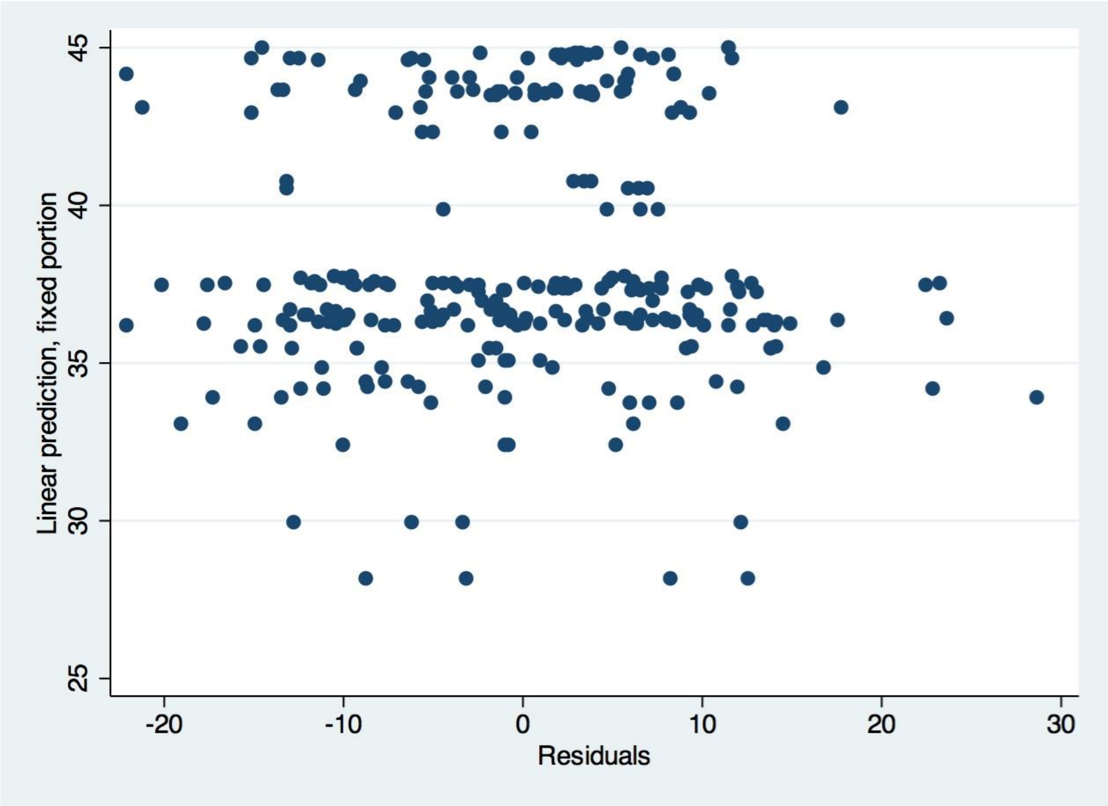

Temperature: operated side

```
Performing EM optimization:

Performing gradient-based optimization:

Iteration 0:   log likelihood = -267.10094
Iteration 1:   log likelihood = -266.82126
Iteration 2:   log likelihood = -266.79504
Iteration 3:   log likelihood = -266.79409
Iteration 4:   log likelihood = -266.79409

Computing standard errors:

Mixed-effects ML regression                     Number of obs      =       274
Group variable: ID                              Number of groups   =        69

                                                Obs per group: min =         3
                                                               avg =       4.0
                                                               max =         4


                                                Wald chi2(4)       =      2.58
Log likelihood = -266.79409                     Prob > chi2        =    0.6310
```

temp	Coef.	Std. Err.	z	P>|z|	[95% Conf.	Interval]
Group	.0803383	.1338169	0.60	0.548	-.181938	.3426147
timesurgery	-.0012583	.0048451	-0.26	0.795	-.0107545	.0082379
Sex	-.1970415	.1436324	-1.37	0.170	-.4785557	.0844728
med	.013663	.0246633	0.55	0.580	-.0346761	.0620021
_cons	36.51758	.2686016	135.95	0.000	35.99113	37.04403

Random-effects Parameters	Estimate	Std. Err.	[95% Conf.	Interval]
ID: Independent				
sd(Group)	.0857412	.2137317	.0006477	11.35079
sd(_cons)	.4624493	.1127114	.2868159	.7456328
sd(Residual)	.5342431	.0263678	.4849841	.5885053

```
LR test vs. linear regression:       chi2(2) =     64.40   Prob > chi2 = 0.0000

Note: LR test is conservative and provided only for reference.
```

Plot: fitted against residuals

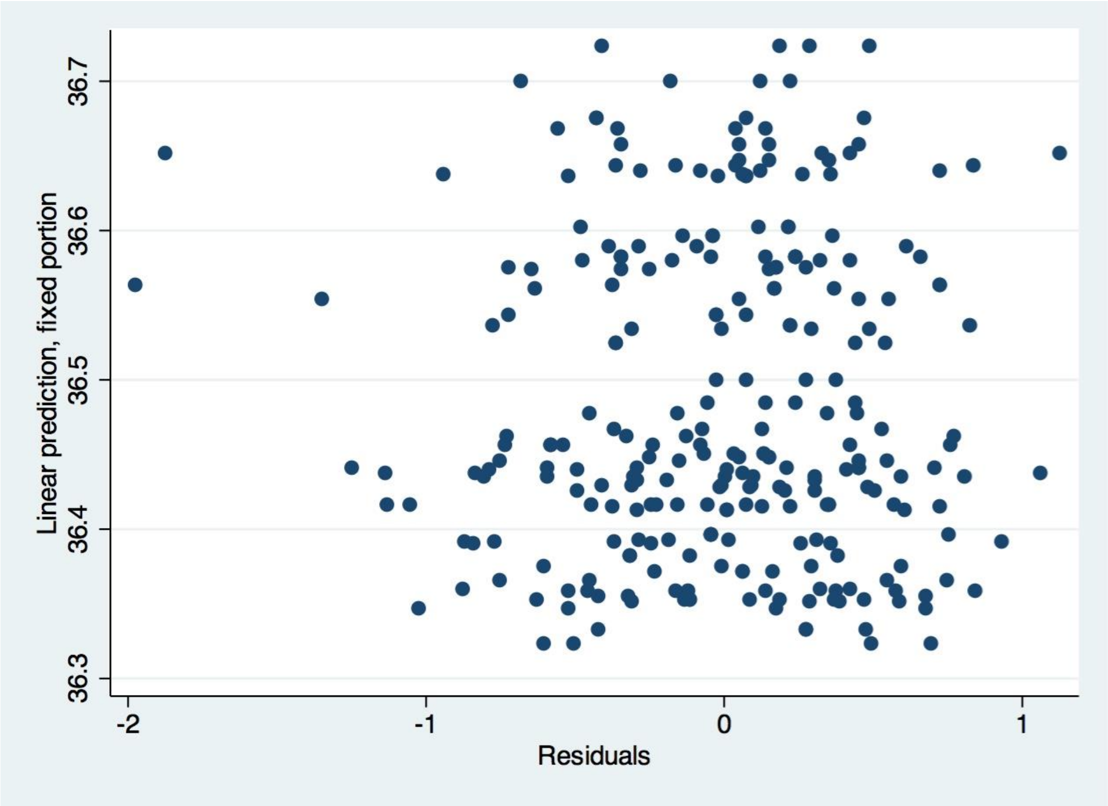

Temperature: opposite side

```
Performing EM optimization:

Performing gradient-based optimization:

Iteration 0:   log likelihood = -292.01417
Iteration 1:   log likelihood = -291.06437
Iteration 2:   log likelihood = -290.96616
Iteration 3:   log likelihood = -290.96413
Iteration 4:   log likelihood = -290.96413

Computing standard errors:

Mixed-effects ML regression                     Number of obs      =       274
Group variable: ID                              Number of groups   =        69

                                                Obs per group: min =         3
                                                               avg =       4.0
                                                               max =         4


                                                Wald chi2(4)       =      7.02
Log likelihood = -290.96413                     Prob > chi2        =    0.1347

------------------------------------------------------------------------------
        temp |      Coef.   Std. Err.      z    P>|z|     [95% Conf. Interval]
-------------+----------------------------------------------------------------
       Group |   .2474844   .1330155     1.86   0.063    -.0132211    .5081899
 timesurgery |  -.0056981   .0048031    -1.19   0.235     -.015112    .0037158
         Sex |  -.1997141   .1424685    -1.40   0.161    -.4789473    .0795191
         med |   .0150508   .0245619     0.61   0.540    -.0330896    .0631912
       _cons |   36.33478   .2688439   135.15   0.000     35.80786     36.8617
------------------------------------------------------------------------------

------------------------------------------------------------------------------
  Random-effects Parameters  |   Estimate   Std. Err.     [95% Conf. Interval]
-----------------------------+------------------------------------------------
ID: Independent              |
                  sd(Group)  |   1.35e-07   3.90e-07      4.83e-10     .000038
                  sd(_cons)  |   .4567243   .0567031      .3580767    .5825486
-----------------------------+------------------------------------------------
               sd(Residual)  |   .6025483   .0297411      .5469876    .6637527
------------------------------------------------------------------------------
LR test vs. linear regression:       chi2(2) =    42.72   Prob > chi2 = 0.0000

Note: LR test is conservative and provided only for reference.
```

Plot: fitted against residuals

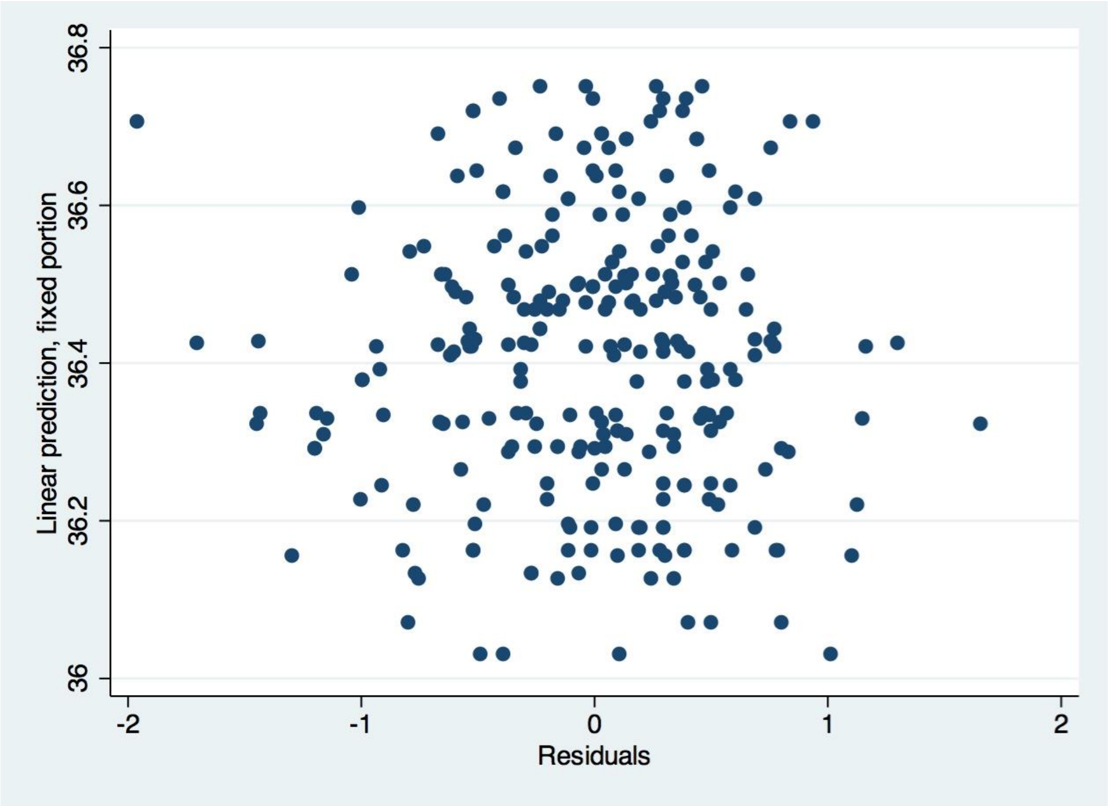

Systemic temperature

```
Performing EM optimization:

Performing gradient-based optimization:

Iteration 0:   log likelihood = -167.68578
Iteration 1:   log likelihood = -166.32266
Iteration 2:   log likelihood = -166.26049
Iteration 3:   log likelihood = -166.26036
Iteration 4:   log likelihood = -166.26036

Computing standard errors:

Mixed-effects ML regression                     Number of obs     =        274
Group variable: ID                              Number of groups  =         69

                                                Obs per group: min =         3
                                                               avg =       4.0
                                                               max =         4

                                                Wald chi2(4)      =       0.99
Log likelihood = -166.26036                     Prob > chi2       =     0.9106
```

temp	Coef.	Std. Err.	z	P>|z|	[95% Conf.	Interval]
Group	.0594662	.0790082	0.75	0.452	-.095387	.2143194
timesurgery	-.0013017	.0028523	-0.46	0.648	-.0068922	.0042887
Sex	-.0353813	.0846143	-0.42	0.676	-.2012222	.1304596
med	.0045001	.0145871	0.31	0.758	-.0240902	.0330904
_cons	36.88301	.1596758	230.99	0.000	36.57005	37.19597

Random-effects Parameters	Estimate	Std. Err.	[95% Conf.	Interval]
**ID: Independent**				
sd(Group)	3.32e-11	8.54e-11	2.17e-13	5.10e-09
sd(_cons)	.2595986	.0353688	.1987609	.3390577
sd(Residual)	.390793	.0192815	.3547718	.4304716

```
LR test vs. linear regression:        chi2(2) =     30.66   Prob > chi2 = 0.0000

Note: LR test is conservative and provided only for reference.
```

Plot: fitted against residuals

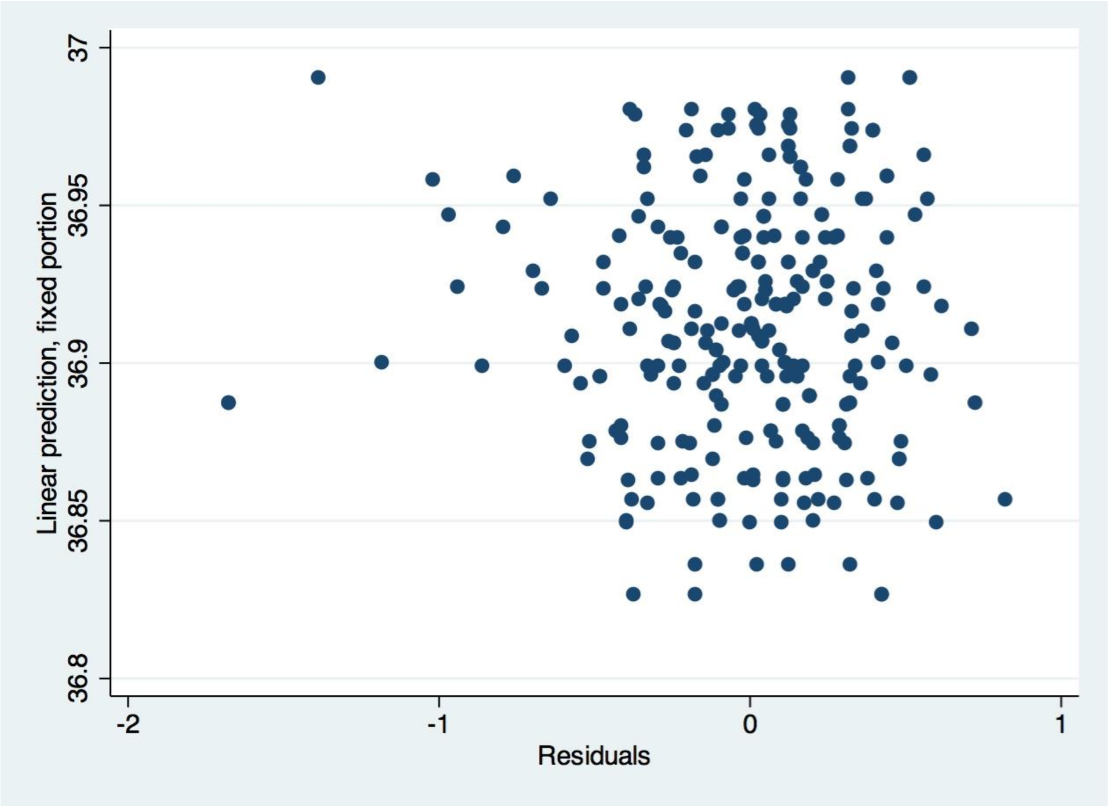

Lymph Nodes

```
Performing EM optimization:

Performing gradient-based optimization:

Iteration 0:   log likelihood =  287.84039
Iteration 1:   log likelihood =  290.07973
Iteration 2:   log likelihood =  290.09604
Iteration 3:   log likelihood =   290.0961
Iteration 4:   log likelihood =   290.0961

Computing standard errors:

Mixed-effects ML regression                     Number of obs      =       276
Group variable: ID                              Number of groups   =        69

                                                Obs per group: min =         4
                                                               avg =       4.0
                                                               max =         4


                                                Wald chi2(4)       =      1.52
Log likelihood =   290.0961                     Prob > chi2        =    0.8229
```

lymphnode	Coef.	Std. Err.	z	P>|z|	[95% Conf.	Interval]
Group	-.0013498	.0102588	-0.13	0.895	-.0214567	.0187572
timesurgery	.0003262	.0003713	0.88	0.380	-.0004016	.0010539
Sex	-.0054792	.0108935	-0.50	0.615	-.0268302	.0158717
med	-.0013908	.0019002	-0.73	0.464	-.0051151	.0023334
_cons	.0040795	.0204373	0.20	0.842	-.0359768	.0441358

Random-effects Parameters	Estimate	Std. Err.	[95% Conf.	Interval]
ID: Independent				
sd(Group)	1.29e-12	.	.	.
sd(_cons)	5.88e-12	.	.	.
sd(Residual)	.0845839	.	.	.

```
LR test vs. linear regression:       chi2(2) =      0.00    Prob > chi2 = 1.0000

Note: LR test is conservative and provided only for reference.
```

Not enough variation for random-effects parameters.

Random-effects GLS tested.

```
Random-effects GLS regression                   Number of obs      =       276
Group variable: ID                              Number of groups   =        69

R-sq:  within  =      .                         Obs per group: min =         4
       between = 0.0224                                        avg =       4.0
       overall = 0.0055                                        max =         4

                                                Wald chi2(4)       =      1.47
corr(u_i, X)   = 0 (assumed)                    Prob > chi2        =    0.8324
```

lymphnode	Coef.	Std. Err.	z	P>|z|	[95% Conf.	Interval]
Group	-.0013498	.0104447	-0.13	0.897	-.021821	.0191215
timesurgery	.0003262	.000378	0.86	0.388	-.0004148	.0010671
Sex	-.0054792	.0110909	-0.49	0.621	-.027217	.0162585
med	-.0013908	.0019346	-0.72	0.472	-.0051825	.0024009
_cons	.0040795	.0208075	0.20	0.845	-.0367025	.0448615
sigma_u	.00651205					
sigma_e	.08512565					
rho	.00581808	(fraction of variance due to u_i)				
