# Supplementary material for: Low-level laser treatment applied at auriculotherapy points to reduce postoperative pain in third molar surgery: A randomized, controlled, single-blinded study
Source: PLoS One. 2018 Jun 19;13(6):e0197989. doi: 10.1371/journal.pone.0197989 (PMC6007895; doi:10.1371/journal.pone.0197989)
Supplement: S8 File — (DOCX) [file pone.0197989.s008.docx]

Table 1. Secondary analysis: Mixed effect models to explain baseline variation – Pain

|  | **Model 1** |  |  | **Model 2** |  |  | **Model 3** |  |  | **Model4** |  |  |
| --- | --- | --- | --- | --- | --- | --- | --- | --- | --- | --- | --- | --- |
| **Fixed effects** | **Estimate** | **SE** | **p value** | **Estimate** | **SE** | **p value** | **Estimate** | **SE** | **p value** | **Estimate** | **SE** | **p value** |
| Intercept | 1.995 | 0.210 | <0.001 | 0.058 | 0.298 | 0.603 | 0.154 | 0.324 | 0.441 | -0.226 | 0.340 | 0.706 |
| 12 h |  |  |  | 4.034 | 0.347 | <.0001 | 4.033 | 0.347 | <.0001 | 4.033 | 0.337 | <.0001 |
| 24 h |  |  |  | 2.094 | 0.347 | <.0001 | 2.094 | 0.347 | <.0001 | 2.095 | 0.337 | <.0001 |
| 7 days |  |  |  | 1.594 | 0.351 | <.0001 | 1.594 | 0.351 | <.0001 | 1.584 | 0.341 | <.0001 |
| Group |  |  |  |  |  |  | -0.193 | 0.249 | 0.444 | -0.216 | 0.243 | 0.380 |
| Medication |  |  |  |  |  |  |  |  |  | 1.212 | 0.335 | <.0001 |
| **Random effects** | **Variance**  **Estimate** | **SD** | **P value** | **Variance**  **Estimate** | **SD** | **P value** | **Variance**  **Estimate** | **SD** | **P value** | **Variance**  **Estimate** | **SD** | **P value** |
| Subjects (intercept) | 0.808 | 0.899 | 0.021 | 1.105 | 1.051 | 0.002 | 1.119 | 1.058 | 0.003 | 1.285 | 1.134 | 0.002 |
| Residuals | 6.807 | 2.609 | <0.001 | 4.456 | 2.111 | <.0001 | 4.458 | 2.111 | <.0001 | 4.209 | 2.052 | <.0001 |
| AIC | 1418.452 |  |  | 1308.823 |  |  | 1309.162 |  |  | 1296.926 |  |  |
|  |  |  |  |  |  |  |  |  |  |  |  |  |


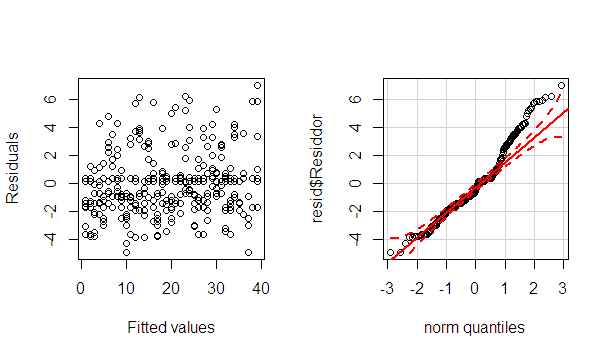


Figure 1. Pain fitted values versus residuals and normal probability plot.

Table 2. Secondary analysis: Mixed effect models to explain baseline variation – Mouth oppening.

|  | **Model 1** |  |  | **Model 2** |  |  | **Model 3** |  |  | **Model4** |  |  |
| --- | --- | --- | --- | --- | --- | --- | --- | --- | --- | --- | --- | --- |
| **Fixed effects** | **Estimate** | **SE** | **p value** | **Estimate** | **SE** | **p value** | **Estimate** | **SE** | **p value** | **Estimate** | **SE** | **p value** |
| Intercept | 38.197 | 1.317 | <0.001 | 46.422 | 1.520 | <0.001 | 47.008 | 1.597 | <0.001 | 47.953 | 1.633 | <0.001 |
| 12 h |  |  |  | -15.573 | 1.292 | <0.001 | -15.573 | 1.290 | <0.001 | -15.573 | 1.285 | <0.001 |
| 24 h |  |  |  | -13.070 | 1.292 | <0.001 | -13.070 | 1.290 | <0.001 | -13.070 | 1.285 | <0.001 |
| 7 days |  |  |  | -4.207 | 1.303 | 0.001 | -4.200 | 1.301 | 0.001 | -4.172 | 1.295 | 0.001 |
| Group |  |  |  |  |  |  | -1.148 | 0.953 | 0.202 | -1.092 | 0.949 | 0.230 |
| Medication |  |  |  |  |  |  |  |  |  | -2.998 | 1.385 | 0.040 |
| **Random effects** | **Variance**  **Estimate** | **SD** | **P value** | **Variance**  **Estimate** | **SD** | **P value** | **Variance**  **Estimate** | **SD** | **P value** | **Variance**  **Estimate** | **SD** | **P value** |
| Subjects (intercept) | 52.270 | 7.230 | <0.001 | 57.100 | 7.556 | <0.001 | 57.280 | 7.568 | <0.001 | 54.780 | 7.402 | <0.001 |
| Residuals | 105.151 | 10.250 | <0.001 | 59.270 | 7.699 | <0.001 | 59.130 | 7.690 | <0.001 | 58.610 | 7.656 | <0.001 |
|  |  |  |  |  |  |  |  |  |  |  |  |  |
| AIC | 2168.453 |  |  | 2015.5 |  |  | 2015.5 |  |  | 2008.3 |  |  |
|  |  |  |  |  |  |  |  |  |  |  |  |  |


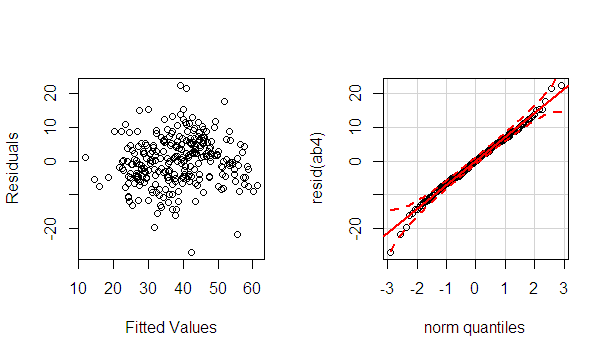


Figure 2. Mouth openned fitted values versus residuals and normal probability plot.

Table 3. Secondary analysis: Mixed effect models to explain baseline variation – Edema.

|  | **Model 1** |  |  | **Model 2** |  |  | **Model 3** |  |  | **Model4** |  |  |
| --- | --- | --- | --- | --- | --- | --- | --- | --- | --- | --- | --- | --- |
| **Fixed effects** | **Estimate** | **SE** | **p value** | **Estimate** | **SE** | **p value** | **Estimate** | **SE** | **p value** | **Estimate** | **SE** | **p value** |
| Intercept | 13.375 | 0.140 | <0.001 | 13.294 | 0.259 | <0.001 | 11.399 | 0.153 | <0.001 | 11.355 | 0.155 | <0.001 |
| 12 h |  |  |  | 0.188 | 0.356 | 0.597 | 0.188 | 0.090 | 0.038 | 0.188 | 0.090 | 0.038 |
| 24 h |  |  |  | 0.104 | 0.356 | 0.770 | 0.104 | 0.090 | 0.250 | 0.104 | 0.090 | 0.250 |
| 7 days |  |  |  | 0.027 | 0.361 | 0.939 | 0.053 | 0.092 | 0.564 | 0.053 | 0.092 | 0.564 |
| Group |  |  |  |  |  |  | 3.789 | 0.064 | <0.001 | 3.789 | 0.064 | <0.001 |
| Medication |  |  |  |  |  |  |  |  |  | 0.129 | 0.099 | 0.195 |
| **Random effects** | **Variance**  **Estimate** | **SD** | **P value** | **Variance**  **Estimate** | **SD** | **P value** | **Variance**  **Estimate** | **SD** | **P value** | **Variance**  **Estimate** | **SD** | **P value** |
| Subjects (intercept) | 0.130 | 0.361 | 0.225 | 0.123 | 0.351 | 0.2376 | 0.644 | 0.802 | <0.001 | 0.629 | 0.793 | <0.001 |
| Residuals | 4.395 | 2.096 | <0.001 | 4.444 | 2.108 | <0.001 | 0.286 | 0.535 | <0.001 | 0.286 | 0.535 | <0.001 |
|  |  |  |  |  |  |  |  |  |  |  |  |  |
| AIC | 1200.3 |  |  | 1201.3 |  |  | 551.0 |  |  | 552.1 |  |  |
|  |  |  |  |  |  |  |  |  |  |  |  |  |


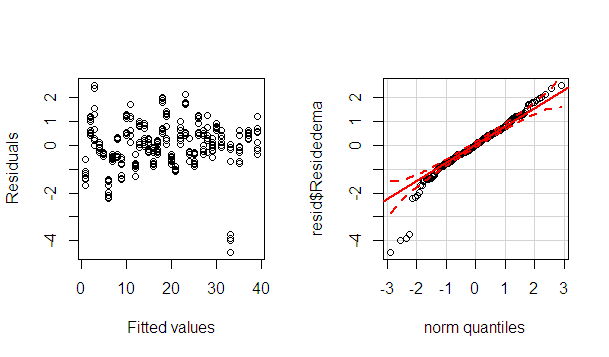


Figure 3. Edema fitted values versus residuals and normal probability plot.
