# Supplementary figures and images for: Low-level laser treatment applied at auriculotherapy points to reduce postoperative pain in third molar surgery: A randomized, controlled, single-blinded study
Source: PLoS One. 2018 Jun 19;13(6):e0197989. doi: 10.1371/journal.pone.0197989 (PMC6007895; doi:10.1371/journal.pone.0197989)

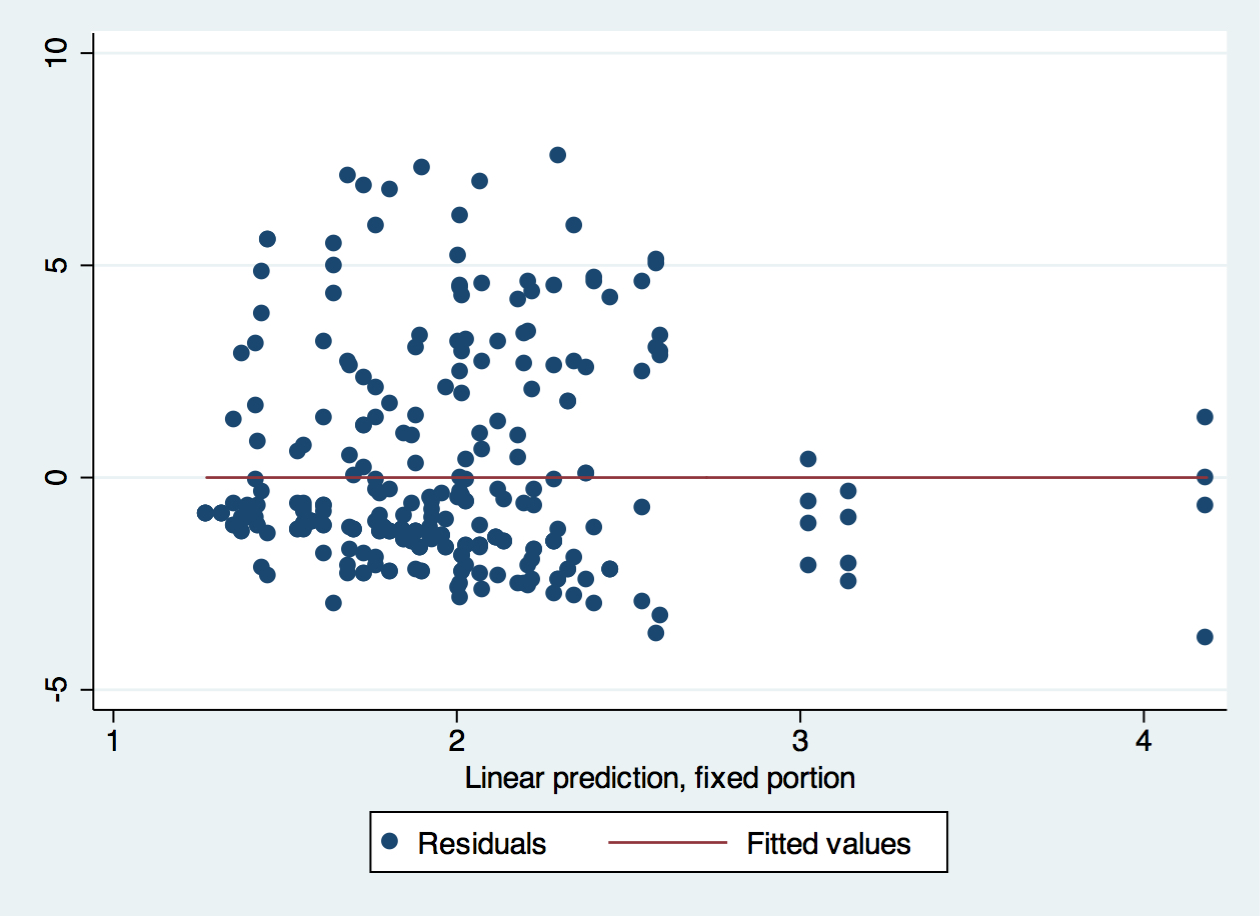

Supplement: S1 Fig — X axis—residuals, Y axis–predicted values. (JPG) [file pone.0197989.s009.jpg]

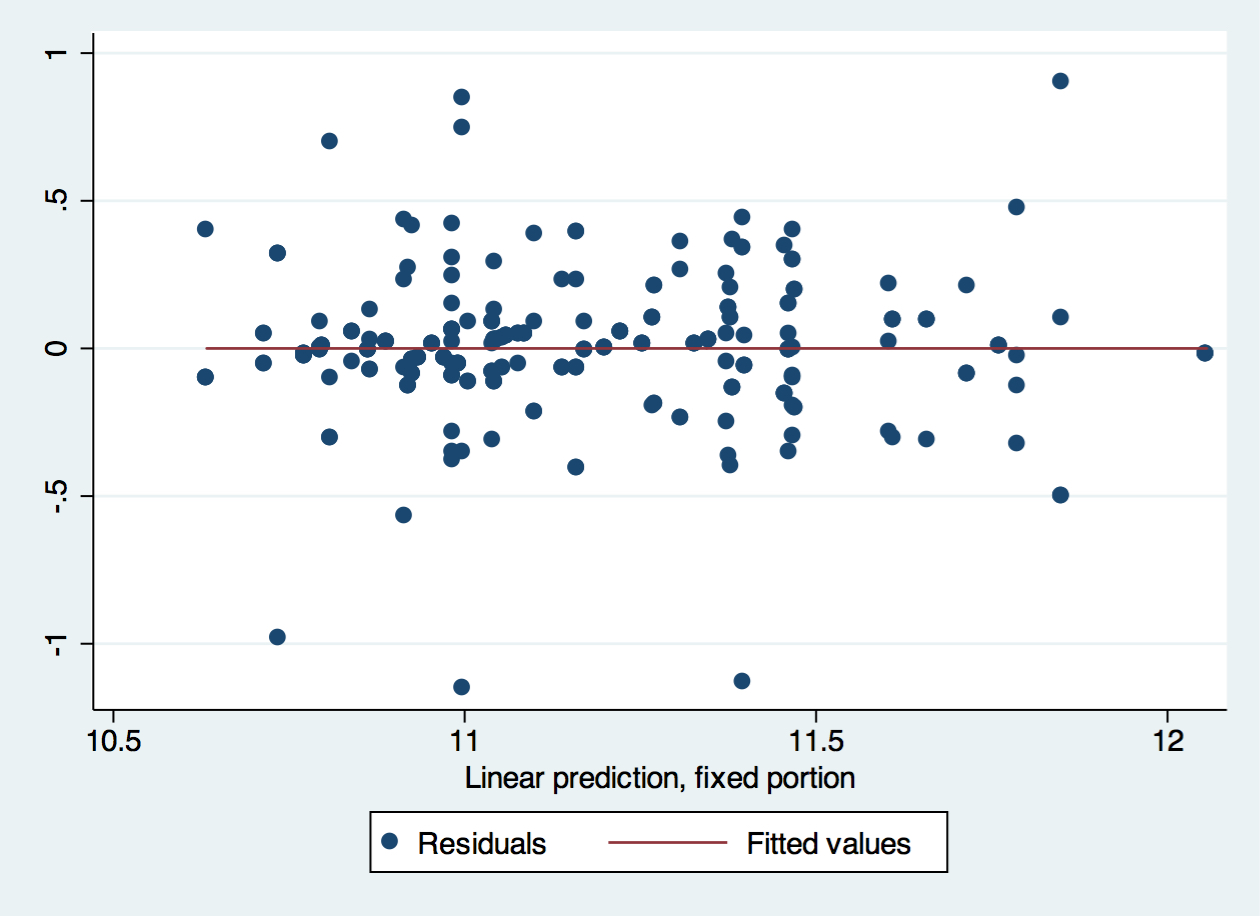

Supplement: S2 Fig — X axis—residuals, Y axis–predicted values. (JPG) [file pone.0197989.s010.jpg]

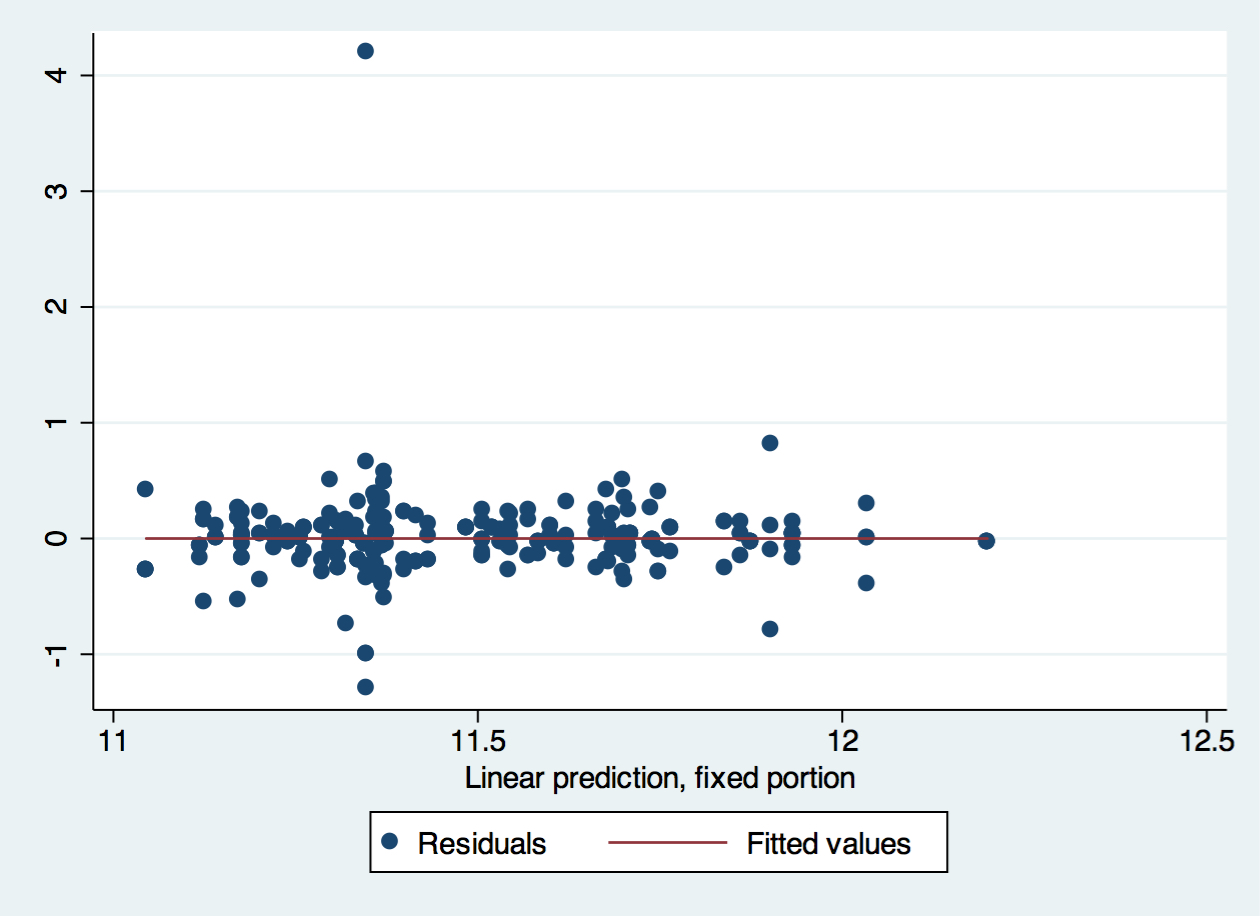

Supplement: S3 Fig — X axis—residuals, Y axis–predicted values. (JPG) [file pone.0197989.s011.jpg]

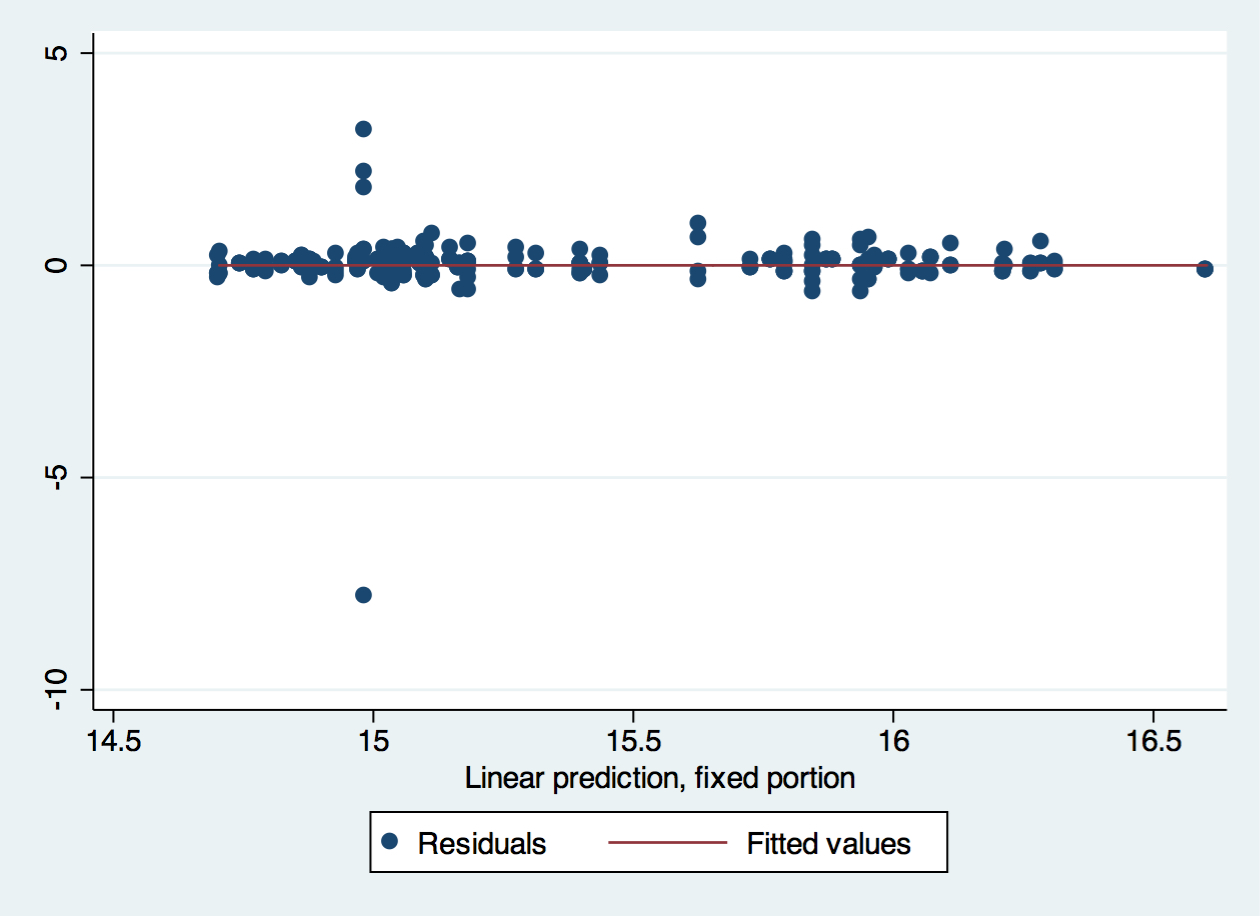

Supplement: S4 Fig — X axis—residuals, Y axis–predicted values. (JPG) [file pone.0197989.s012.jpg]

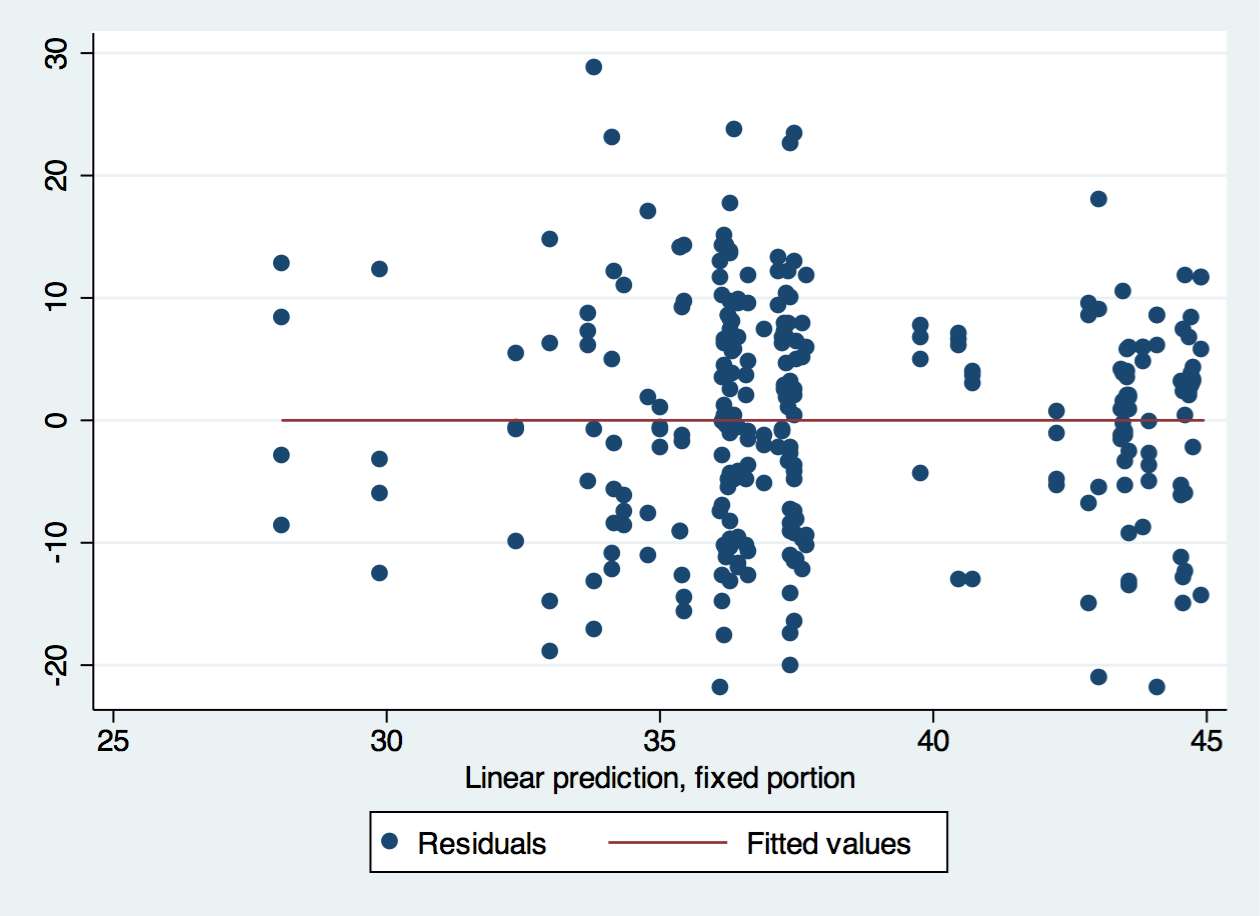

Supplement: S5 Fig — X axis—residuals, Y axis–predicted values. (JPG) [file pone.0197989.s013.jpg]

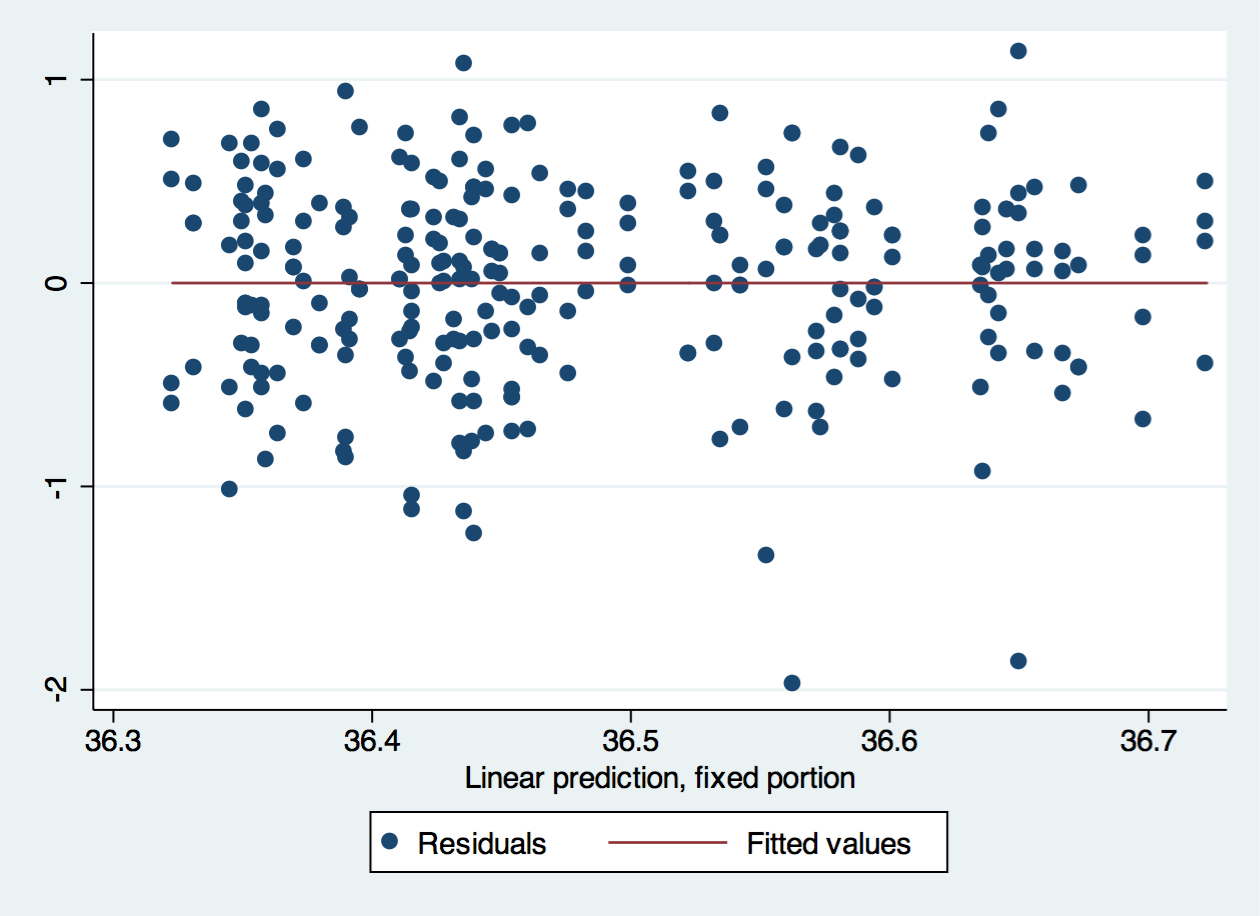

Supplement: S6 Fig — X axis—residuals, Y axis–predicted values. (JPG) [file pone.0197989.s014.jpg]

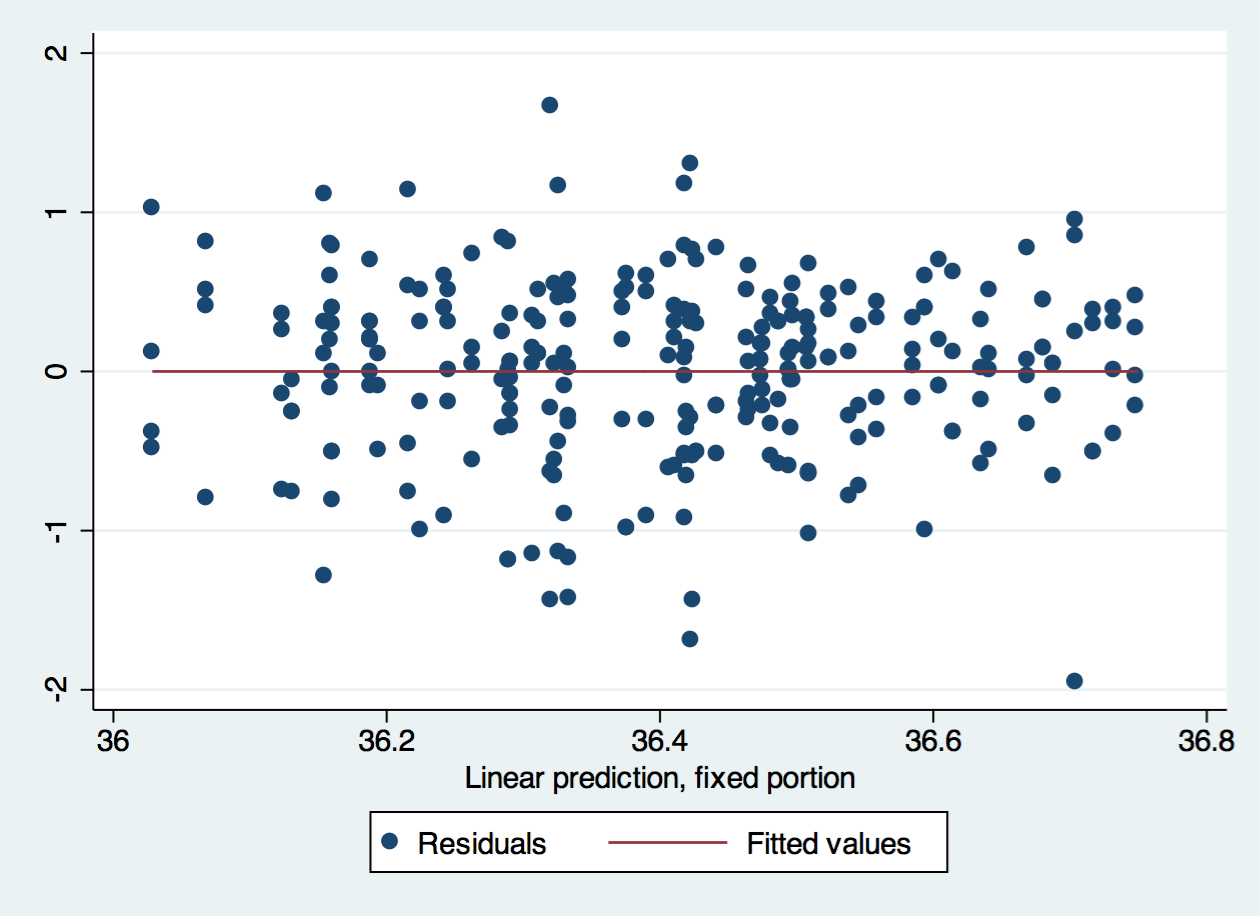

Supplement: S7 Fig — X axis—residuals, Y axis–predicted values. (JPG) [file pone.0197989.s015.jpg]

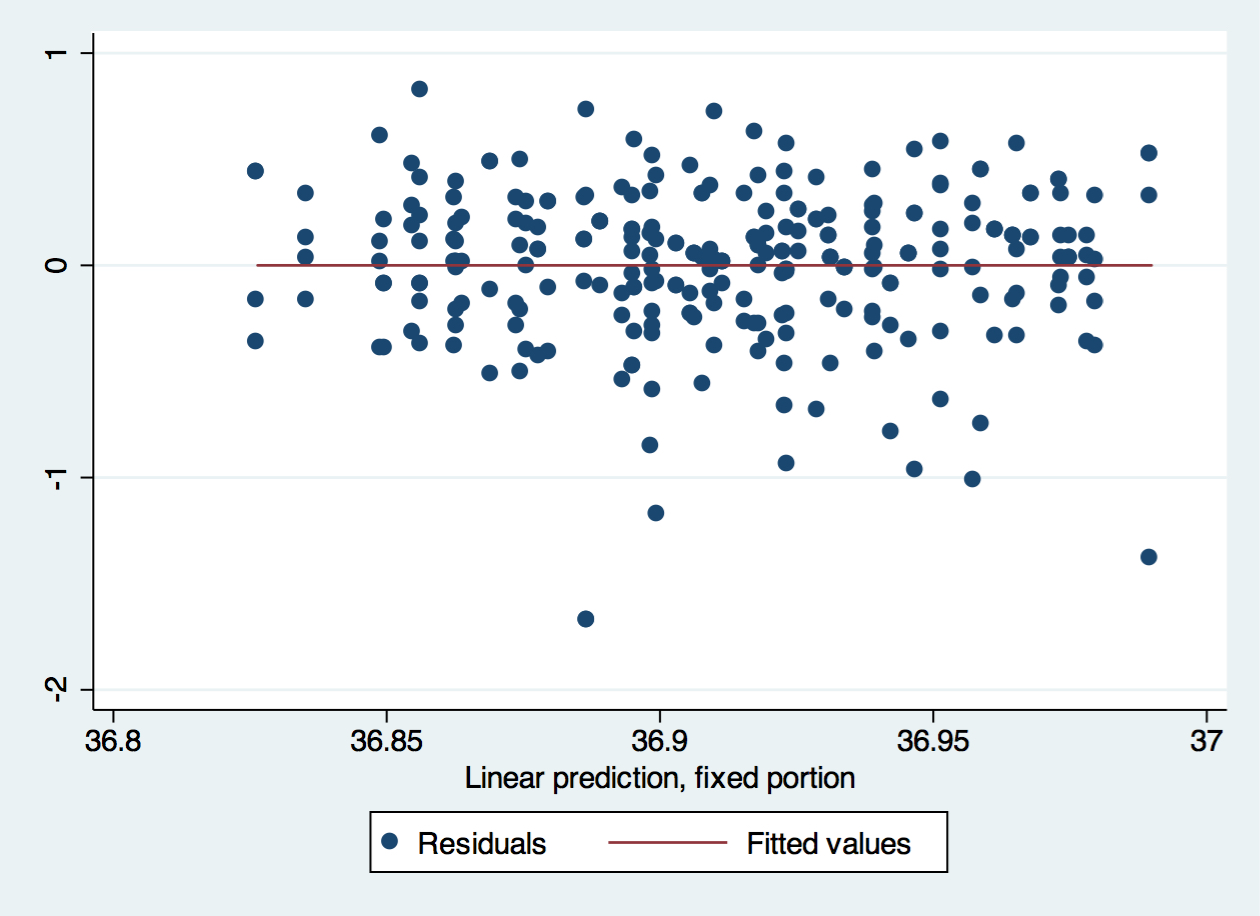

Supplement: S8 Fig — X axis—residuals, Y axis–predicted values. (JPG) [file pone.0197989.s016.jpg]

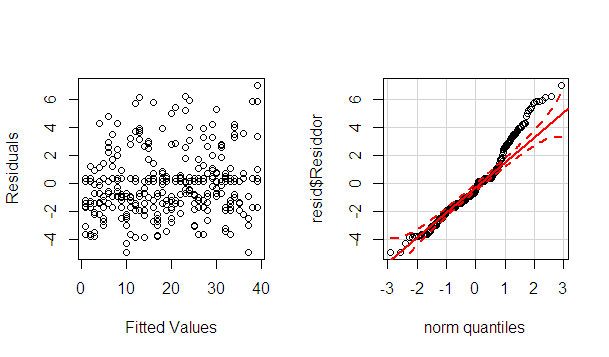

Supplement: S9 Fig — X axis—residuals, Y axis–Fitted values (TIFF) [file pone.0197989.s017.tiff]

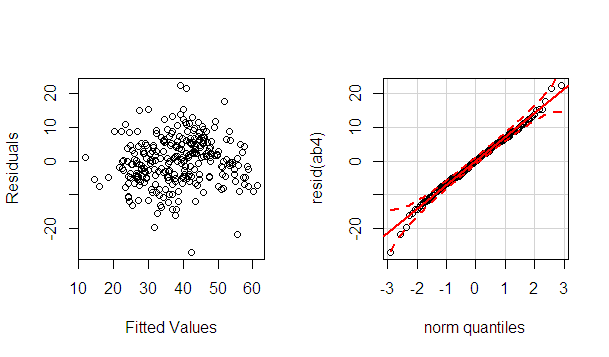

Supplement: S10 Fig — X axis—residuals, Y axis–Fitted values. (TIFF) [file pone.0197989.s018.tiff]

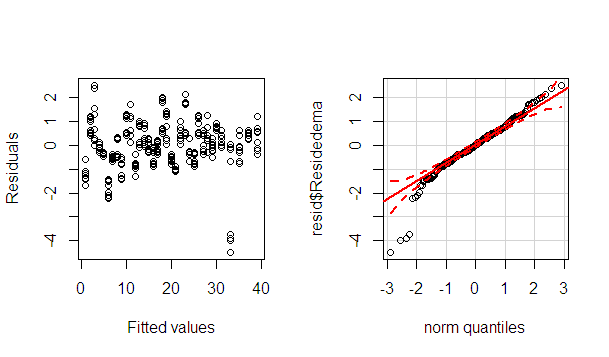

Supplement: S11 Fig — X axis—residuals, Y axis–Fitted values. (TIFF) [file pone.0197989.s019.tiff]
